# A chimeric RNA consisting of siRNA and aptamer for inhibiting dengue virus replication

**DOI:** 10.1093/narmme/ugae025

**Published:** 2024-12-25

**Authors:** Ryo Amano, Masaki Takahashi, Kazumi Haga, Mizuki Yamamoto, Kaku Goto, Akiko Ichinose, Michiaki Hamada, Jin Gohda, Jun-ichiro Inoue, Yasushi Kawaguchi, Meng Ling Moi, Yoshikazu Nakamura

**Affiliations:** Project Division of RNA Medical Science, The Institute of Medical Science, The University of Tokyo, 4-6-1, Shirokanedai, Minato-ku, Tokyo 108-8639, Japan; Project Division of RNA Medical Science, The Institute of Medical Science, The University of Tokyo, 4-6-1, Shirokanedai, Minato-ku, Tokyo 108-8639, Japan; RIBOMIC Inc., 3-16-13, Shirokanedai, Minato-ku, Tokyo 108-0071, Japan; Department of Developmental Medical Sciences, School of International Health, Graduate School of Medicine, The University of Tokyo, 7-3-1, Hongo, Bunkyo-ku, Tokyo 113-0033, Japan; Research Center for Asian Infectious Diseases, The Institute of Medical Science, The University of Tokyo, 4-6-1, Shirokanedai, Minato-ku, Tokyo 108-8639, Japan; Project Division of RNA Medical Science, The Institute of Medical Science, The University of Tokyo, 4-6-1, Shirokanedai, Minato-ku, Tokyo 108-8639, Japan; Graduate School of Advanced Science and Engineering, Waseda University, 3-4-1, Okubo, Shinjuku-ku, Tokyo 169-8555, Japan; Graduate School of Advanced Science and Engineering, Waseda University, 3-4-1, Okubo, Shinjuku-ku, Tokyo 169-8555, Japan; Research Center for Asian Infectious Diseases, The Institute of Medical Science, The University of Tokyo, 4-6-1, Shirokanedai, Minato-ku, Tokyo 108-8639, Japan; The University of Tokyo Pandemic Preparedness, Infection and Advanced Research Center (UTOPIA), 4-6-1, Shirokanedai, Minato-ku, Tokyo 108-8639, Japan; Research Center for Asian Infectious Diseases, The Institute of Medical Science, The University of Tokyo, 4-6-1, Shirokanedai, Minato-ku, Tokyo 108-8639, Japan; The University of Tokyo Pandemic Preparedness, Infection and Advanced Research Center (UTOPIA), 4-6-1, Shirokanedai, Minato-ku, Tokyo 108-8639, Japan; Division of Molecular Virology, Department of Microbiology and Immunology, The Institute of Medical Science, The University of Tokyo, 4-6-1, Shirokanedai, Minato-ku, Tokyo 108-8639, Japan; Department of Developmental Medical Sciences, School of International Health, Graduate School of Medicine, The University of Tokyo, 7-3-1, Hongo, Bunkyo-ku, Tokyo 113-0033, Japan; The University of Tokyo Pandemic Preparedness, Infection and Advanced Research Center (UTOPIA), 4-6-1, Shirokanedai, Minato-ku, Tokyo 108-8639, Japan; Project Division of RNA Medical Science, The Institute of Medical Science, The University of Tokyo, 4-6-1, Shirokanedai, Minato-ku, Tokyo 108-8639, Japan; RIBOMIC Inc., 3-16-13, Shirokanedai, Minato-ku, Tokyo 108-0071, Japan

## Abstract

Silencing viruses by chimeric RNAs, wherein small interfering RNAs (siRNAs) targeting viral RNAs are conjugated with RNA aptamers specific to viral envelope proteins, is a promising treatment for viral infection diseases; however, practical evaluations are apparently lacking. Here, we present a chimeric RNA comprises siRNA and RNA aptamer, both of which target all four serotypes of dengue virus (DENV), for suppressing DENV replication. The siRNA targeting consensus sequences in the 3′-UTR of all four DENV serotypes suppressed the expression of a reporter gene carrying the siRNA-targeted sequence of DENV-1 by ∼70%. The RNA aptamer generated by VLP-SELEX using DENV-1-VLPs as baits showed an affinity for all four DENV-VLP serotypes, presumably without affecting the fusion process. After conjugation of each modality, the chimeric RNA significantly suppressed authentic DENV-1 and DENV-2 production *in vitro*. Our study provides evidence that chimeric RNA is a potentially effective antiviral agent.

## Graphical Abstract

**Graphical Abstract:**
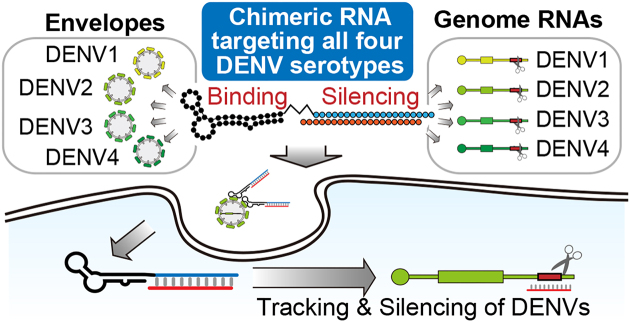

## Introduction

Dengue viruses (DENVs), four serotypes of which (DENV-1 to -4) have been identified (1), are mosquito-borne viruses and cause dengue fever, more severe dengue hemorrhagic fever, and dengue shock syndrome, as a potentially lethal disease (2). The World Health Organization (WHO) warned that cases of dengue fever could reach close to record highs in 2023 (https://www.who.int/emergencies/disease-outbreak-news/item/2023-DON498). Although >80 years have passed since the first isolation of the virus in Nagasaki (Japan) in 1943 (3), the development of therapeutic agents for DENV disease has stagnated while vaccine development such QDENGA® (4) has gradually progressed. One of the reasons for the stagnation of therapeutic approaches is that conventional antibody-based treatment has a risk to potentially lead to antibody-dependent enhancement (ADE), which induces severe symptoms, while neutralizing antibodies cannot sufficiently neutralize other DENV serotypes or closely related viruses (5). To accelerate drug discovery for DENV, a modality shift from conventional antibodies may be crucial for developing DENV treatments without adverse effects such as ADE.

RNA aptamers are single-stranded oligonucleotides specifically binding to the target with a high shape complementary (6) and have been marketed as approved drugs (7). Aptamers have been anticipated to be effective virus inhibitors with limited adverse effects, including toxicity, antigenicity, viral drug resistance and ADE, due to a non-antibody modality (8–10). In the context of ease of use, aptamers targeting viral envelope proteins have fewer drug components as aptamers do not need delivery systems. Recently, we established and reported an advanced Systematic Evolution of Ligands by EXponential enrichment (SELEX) system using virus-like particles (VLPs) as baits for the discovery of human cell surface proteins, named VLP-SELEX (11). This system is expected to serve as a robust platform technology to generate virus neutralizing aptamers. At present, however, while aptamers with a broad cross-binding ability to cognate targeted-viruses such as DENV all serotypes can be developed, achieving a balanced and potent cross-neutralizing-activity across DENV all serotypes can be challenging. Together with aptamers, small interfering RNAs (siRNAs) are also known as a promising candidate of anti-virus agent for degrading virus genome itself and/or its RNA derivatives (12), due to their specificity and efficiency (13–15). By targeting conserved RNA sequences in the target viruses, siRNAs may easily achieve a high specificity with wide cross-reactivity compared with aptamers. However, siRNA-based therapeutics (16) could present a different set of challenges in efficacy, due to the need for optional delivery to target organs (16).

In this context, to address the shortcomings of the two oligonucleotide modalities and to leverage on the advantages of both modalities, a chimeric RNA consisting of an RNA aptamer-conjugated siRNA has been generated and tested as an advanced oligonucleotide agent for the treatment of various diseases, such as cancer and viral infection (17). For viral infection, however, practical examples are currently limited to studies on HIV-1 by a certain research group (18–22), and thus the effectiveness of chimeric RNA remains unclear due to an insufficient number of practical evaluations.

In the present study, we generated siRNAs against DENV genome RNA and RNA aptamers against DENV envelope protein, both of which target all four serotypes in DENV, and subsequently, the effectiveness of an integrated single molecule, an anti-DENV chimeric RNA was evaluated. Our anti-DENV chimeric RNA inhibited the *in vitro* replication of DENV-2 by ∼90% and significantly suppress DENV-1 and DENV-2 production, suggesting the broad utility of chimeric RNAs as targeted therapy for various viral diseases.

## Materials and methods

### DNA and RNA oligonucleotides

DNAs for primer sets, siRNAs and an artificial gene synthesis for a portion of the DENV-1 genome were synthesized by FASMAC Co., Ltd. (Atsugi, Kanagawa, Japan). The sequences of siRNAs are shown in Supplementary Table S1. Anti-DENV chimeric RNAs were synthesized and purchased by Gene Design Inc. (Ibaraki, Osaka, Japan).

### VLP-SELEX

A slightly modified version of VLP-SELEX was performed (11) and detailed conditions were described in Figure 2A. Briefly, the Lb03 double-stranded (ds) DNA library was constructed by primer extension by primer extension with a forward primer containing a T7 promotor and ExTaq DNA polymerase (TaKaRa Bio, Shiga, Japan). The library and primer sequences are as follows: Lb03, 5′-GAT GTG AGT GTG TGA CGA GT [N40] CAC AGA GAA GAA ACA AGA CCC-3′ (where N40 stands for 40-nucleotide random sequence); Lb03 forward primer, 5′-TAA TAC GAC TCA CTA TAG GGT CTT GTT TCT TCT CTG TG-3′; Lb03 reverse primer, 5′-GAT GTG AGT GTG TGA CGA GT-3′ (T7 promoter sequence is underlined). The library and primers were purchased from GeneDesign, Inc. (Osaka, Japan) and Fasmac Co., Ltd. (Kanagawa, Japan), respectively. The resultant dsDNAs were subjected to *in vitro* transcription using 2′-fluoro-CTP, 2′-fluoro-UTP, ATP, and GTP (at a final concentration of 2.5 mM each) and Y639F mutant T7 RNA polymerase (at a final concentration of 40 ng/μL). After overnight incubation at 37 °C, transcribed products were treated with DNase I (Roche, Basel, Switzerland) at 37 °C for 30 min, and then purified by phenol/chloroform (NACALAI TESQUE, INC.) extraction for protein removal and ultrafiltration with Amicon Ultra 0.5 ml (molecular weight cutoff (MWCO) 30 kDa [Merck Millipore, MA, USA]) for NTPs removal. Before selection, the purified single-stranded RNA (ssRNA) pools were denatured at 95 °C for 5 min in DNase/RNase-free distilled water, refolded by being snap-cooled on ice for 5 min, and incubated in SELEX buffer (20 mM Tris–HCl pH 7.6, 145 mM NaCl, 5.4 mM KCl, 0.8 mM MgCl_2_ and 1.8 mM CaCl_2_) containing Tween80 at final concentration of 0.01% at 25 °C for 10 min in the presence of low-molecular-weight heparin (∼5000 Da) (Dalteparin sodium; Pfizer, Inc., NY, USA) at final concentration of 2 mg/ml and yeast tRNA (Roche) at final concentration of 0.05 mg/ml. Heparin and yeast tRNA were added in each round at the same final concentration despite changes of incubation volume as competitors. The refolded RNA pool and the DENV1-VLPs (The Native Antigen Company Ltd, Oxford, UK) were mixed and incubated for 30–60 min with a rotator. To remove unbound and weakly bound RNAs to VLPs, an ultrafiltration column, Vivaspin 500 with 100 K MWCO polyether sulfone (PES) (Sartorius AG, Goettingen, Germany), was used for the separation by centrifugation at 14,000 × *g* for 5 min. After discarding flow through, 500 μl of SELEX buffer containing 0.01% Tween80 was added, followed by centrifugation several times as a washing process (Figure 2A). Retained solution-containing RNA–VLP complexes in the column were recovered and added to phenol/chloroform for extraction of bound RNAs. After ethanol precipitation with Dr GenTLE Precipitation Carrier (TaKaRa Bio), all amount of the collected RNAs were subjected to reverse transcription with SuperScript IV Reverse Transcriptase (Thermo Fisher Scientific, MA, USA) according to the manufacturer’s protocols, and then the ssDNAs were subjected to polymerase chain reaction (PCR) amplification with ExTaq DNA polymerase until appropriate PCR cycles. The amplified dsDNAs were transcribed with Y639F mutant T7 RNA polymerase and modified NTPs described above. After the first round, to reduce undesired nonspecific adsorption of RNAs to the ultrafiltration column, refolded RNA pools were passed through Vivaspin 500 column (MWCO 100 K) at 14,000 × *g* for 10 min several times before selection with VLPs. After the ninth round, the enriched pool was subjected to high-throughput sequencing (HTS) and *in silico* analysis.

### Sequencing and *in silico* analysis

The HTS procedure was carried out using the Ion PGM system with an Ion 318 chip according to the manufacturer’s protocols (Thermo Fisher Scientific). The number of sequencing reads was 71 448. Sequencing data were analyzed with FASTX-Toolkit (http://hannonlab.cshl.edu/fastx_toolkit) and FASTAptamer (23) as described (11). Briefly, after trimming the accessory sequences such as a barcode, adaptor and T7 promoter sequence, sequences coding aptamers were analyzed. Furthermore, sequences of less than four reads were excluded from this analysis. Subsequently, cluster analysis was carried out with an edit distance set to six; thus, sequences possessing fewer than six base differences were assigned to an identical cluster. Subsequently, the sequences with the highest read number or read per million (RPM) in each cluster were extracted as a representative sequence in each cluster (Supplementary Table S2).

Sequencing data were also analyzed with RaptRanker (24). Alterations of RNA library in each round were examined.

### Surface plasmon resonance analysis

Surface plasmon resonance (SPR) analyses were carried out as previously described using a BIAcore 2000 instrument (Cytiva, MA, USA) with minor modifications. To examine the binding ability of the aptamers to DENV-VLPs, the chemically synthesized 5′-biotinylated oligo d(T)_16_ (Figures 3A, C and 4B) and the chemically synthesized 3′-biotinylated aptamers (Figure 3B and D) were immobilized on a streptavidin (SA) sensor chip (Cytiva, MA, USA) at ∼1000 resonance units (RU) and at ∼1500 RU, respectively. In the case of indirect immobilization using the 5′-biotinylated oligo d(T)_16_, the chemically synthesized 5′-biotinylated oligo d(T)_16_ was immobilized to flow cells 1–4 on the surface of an SA sensor chip at ∼1000 RU, and then at ∼1000 RU of an enzymatically synthesized initial RNA pool on flow cell 1 and the aptamers with poly(A)_16_ tail at the 3′ end on flow cells 2–4 were immobilized mediating hybridization between the oligo d(T)_16_ and the poly A tailed aptamers. SELEX buffer containing 0.01% Tween80 was employed as a running buffer. After immobilization of an initial pool and aptamers, DENV-VLPs at a final concentration of 20 ng/μl in SELEX buffer were injected into flow cells 1–4 at a flow rate of 10 μl/min for 120 s and dissociated for 300 s. In the case of measuring the dissociation constant (*K*_D_ value) of the interaction between aptamer and DENV-1-VLPs, ∼300 RUs of an initial pool and aptamer were immobilized on flow cells 1 and 2, respectively. After immobilization of an initial pool and aptamer, DENV-1-VLPs at final concentrations of 0.0625–2 nM in SELEX buffer were injected into flow cells 1 and 2 at a flow rate of 40 μl/min for 120 s and dissociated for 600 s. The molar concentration of DENV-1-VLP was estimated based on the molecular weight of the DENV-1 particle (PDB ID: 4CCT, ∼11.2 kDa). To regenerate sensor chips, a solution consisting of 20 mM Tris–HCl pH 7.6, 4 M urea and 5 mM EDTA was injected for 1min in the regeneration process. To examine the inhibitory effect of aptamers on antibody–VLP interaction, the recombinant Protein A (BioVision, MA USA) was immobilized to flow cells 1 and 2 on the surface of a CM5 sensor chip (Cytiva) at ∼6000 RU by amino coupling. Then, a mouse monoclonal anti-flavivirus envelope protein antibody 4G2 (The Native Antigen Company Ltd.) at final concentration of 100 nM was injected into flow cell 2 at a flow rate of 2 μl/min for 600 s, resulting in immobilization at ∼3000 RU. In the regeneration process, a 10 mM glycine-HCl (pH 1.5) solution was injected for 1 min. The signal of flow cell 1 was subtracted from that of flow cells 2, 3 and 4 to eliminate nonspecific interactions. The *K*_D_ value was estimated using a Langmuir (1:1) binding model by BIAevaluation software (Cytiva).

### Aptamer truncation

The sequences of the lead aptamer and shortened aptamers are as follows: DENV-Apt, 5′-GGG UCU UGU UUC UUC UCU GUG UGU AAU UUG UUU UAC UGG GGG GUU AAC CUA ACA AGU GCA CAC UCG UCA CAC ACU CAC AUC-3′, DENV-Apt_41, 5′-GGG UAA UUU GUU UUA CUG GGG GGU UAA CCU AAC AAG UGC CC-3′ and DENV-Apt_28, 5′-GGG UUU UAC UGG GGG GUU AAC CUA ACC C-3′, respectively. The secondary structures of aptamers were predicted using the Mfold (http://www.unafold.org). Based on their predicted structures, underlined sequences were truncated and a stem (5′-GG/CC-3′) was added to each aptamer for effective transcription by T7 RNA polymerase and for stabilization of the terminal stem structure, which resulted in a length of 41 and 28 nt without losing the binding activity.

### Construction of plasmid vector and luciferase assay for knockdown efficiency of siRNAs

Knockdown potency of the siRNAs targeting consensus sequences in all four DENV serotypes was examined by a psiCHECK2 vector (Promega, WI, USA) as previously described (25,26). Briefly, the partial DENV-1 sequence targeted by the siRNAs was prepared using PCR and inserted into the 3′-UTR of the *Renilla luciferase* gene onto a psiCHECK2 vector, which was digested with *Not* I enzyme. Partial DENV-1 genome sequences were synthesized by FASMAC Inc. in an artificial gene synthesis service. The synthetic artificial gene sequences and the sequences targeted by siRNAs were shown in Note S1 (DENV-1 genome sequence as NCBI Ref Seq). The ligation reaction was carried out using InFusion HD Cloning Kit (TaKaRa Bio).

The sequences of the PCR primers are as follows:

The ancC primer sets to amplify anchored capsid protein ancC region in DENV-1, DENV-1-Anc-Fwd; 5′-AAA CCT AGA GCG GCC ATG AAC AAC CAA CGG AAA AAG ACG-3′, DENV1-Anc-Rev; 5′-TTG CGG CCA GCG GCC CGC CAG GGC TGT GGG CAG CAG C-3′. The 3′-UTR primer sets to amplify entire 3′-UTR region in DENV1, DENV1-3′-UTR-Fwd; 5′-AAA CCT AGA GCG GCC GCC AAC TCA TTC ACA AAA TAA AGG-3′, DENV1-3′-UTR-Rev; 5′-TTG CGG CCA GCG GCC AGA ACC TGT TGA TTC AAC AGC ACC-3′.

The constructed reporter plasmid and test siRNAs were co-transfected into HEK293 cells using Lipofectamine 2000 transfection reagent (Thermo Fisher Scientific) according to the manufacturer’s instructions. One day after transfection, luciferase activities of the *Renilla* and *Photinus luciferase* were measured by a Dual-Luciferase® Reporter Assay System with a luminometer, Centro XS3 LB 960 (Berthold Technologies, Bad Wildbad, Germany).

### Cell culture

HEK293T cells were obtained from the American Type Culture Collection (ATCC) (Manassas, VA, USA) and were grown at 37°C in DMEM (NACALAI TESQUE, INC., Kyoto, Japan) supplemented with 10% FBS (Invitrogen), 100 U/ml penicillin and 100 μg/ml streptomycin (NACALAI TESQUE, INC.). C6C36 cells were purchased from ATCC (Manassas, VA, USA) and were maintained in Eagle Minimum Essential Medium (EMEM; Wako Pure Chemical Co., Osaka, Japan) containing 10% FBS at 28 °C for C6/36 cells. BHK cells were obtained from Japan Health Science Research Resources Bank, Japan, and were maintained in EMEM (Wako Pure Chemical Co., Osaka, Japan) containing 10% FBS at 37°C.

### Dual split reporter protein (DSP) assay in the 384-well format

Dual split reporter protein (DSP) assay was carried out as described previously (27). Briefly, 2 days before the DSP assay, C6/36 cells were seeded in 24-well culture plates (2 × 10^5^ cells/250 μl). One day before the assay, cells were transfected as follows. For the 24-well culture plates, 1 μg pUb-prME and 2 μg pUb-DSP1-7 or pUb-DSP8-11 plasmid were mixed with 2 μl FlyFectin in 50 μl Opti-MEM (Thermo Fisher Scientific, Waltham, MA, USA). After a 20-min incubation at room temperature, cells were transfected with the plasmid mixture to express prME along with DSP1-7 or DSP8-11. At 2 h before the DSP assay, cells were treated with 6 μM EnduRen (Promega, Madison, WI, USA), which allows esterases inside of the cell to convert EnduRen to coelenterazine, which is an actual substrate for Renilla luciferase (RL). Effector cells expressing prME and DSP1-7 and target cells expressing prME and DSP8-11 were detached from the culture plate by pipetting and mixed on ice. After centrifugation (440 × *g* for 5 min at 4 °C), the supernatant was replaced with 600 μl of EMEM (pH 7.4 or 6.5). Antibodies or aptamers dissolved in PBS were added to the 384-well plates (Greiner Bioscience, Frickenhausen, Germany). Subsequently, the mixture of effector and target cells (100 μl; pH 7.4 or 6.5) was added to the 384-well plates. After incubation at 28°C for 1 h, RL activity was measured using a CentroXS3 microplate reader (Berthold Technologies, Oak Ridge, TN, USA).

### Dengue virus-infection assay

Dengue virus type 2 (DENV-2) D2/Hu/OPD030NIID/2005 strain (GenBank accession no. AB219135) and Dengue virus type-1 (DENV-1) 01-44-1HuNIID strain (GenBank accession no. AB111070) were used in this study. BHK cells, a hamster kidney cell line (Japan Health Science Research Resources Bank, Japan), were cultured in EMEM (Wako, Japan), supplemented with heat inactivated 10% fetal bovine serum (FBS, Invitrogen) at 37°C in 5% CO_2_. A total of 10E^5^ BHK cells was seeded in one well of 12-well plate and left to incubate overnight at 37°C in 5% CO_2_. Cell culture supernatant was then collected and stored at −80 °C until further experiments.

Micro-neutralization assay was performed according to a previous protocol (28). Briefly, BHK cells were seeded at a 96-well plate. At each time point, the cells were treated with chimera RNA prior to virus infection at a 1:1 volume ratio. For siRNA transfection, the cells were treated with siRNA-aptamer in complex with transfection reagent (Lipofectamine RNAiMAX, Thermo Fisher Scientific) and further incubated for either 24-h or 5-min prior to virus infection. After a 1-h virus adsorption, the plates were incubated up to 7 days and analyzed at each specific time-points. The cells were then fixed and stained with methyl alcohol (Wako Pure Chemical Industries) and 0.1% crystal violet. Excess dye was then removed and 1% sodium dodecyl sulfate (SDS) were added to dissolve the cells and release the dye. Absorbance was detected at OD 595 nm to determine viable cells against the percentage of dead cells between the control and test group.

### PCR for detecting the DENV genome

Viral RNA was extracted using a Zymo virus RNA extraction kit (Zymo Research, Irvine, CA, USA), and DENV serotype 2 was determined using serotype-specific reverse transcriptase polymerase chain reaction (RT-PCR) (29,30).

### Multiple sequence alignment

In Supplementary Figure S4, sequence alignment was performed using the Clustal Omega with default parameters (https://www.uniprot.org/align). The sequences of the ectodomain of DENV E protein from DENV-1 (strain uerto_Rico/US/BID-V853/1998), DENV-2 (strain Thailand/16681/84), DENV-3 (strain Sri_Lanka_D3/H/IMTSSA-SRI/2000/1266) and DENV-4 (strain Dominica/814669/1981) were used. Aligned sequences with secondary structure information was generated by ESPript 3.0 (31) and edited using Adobe Illustrator. The tertiary structure of the ectodomain of the DENV-2 E protein (PDB ID: 3C6E) is visualized by UCSF Chimera X (32).

### *S*tatistical analysis

**Figure 1.**
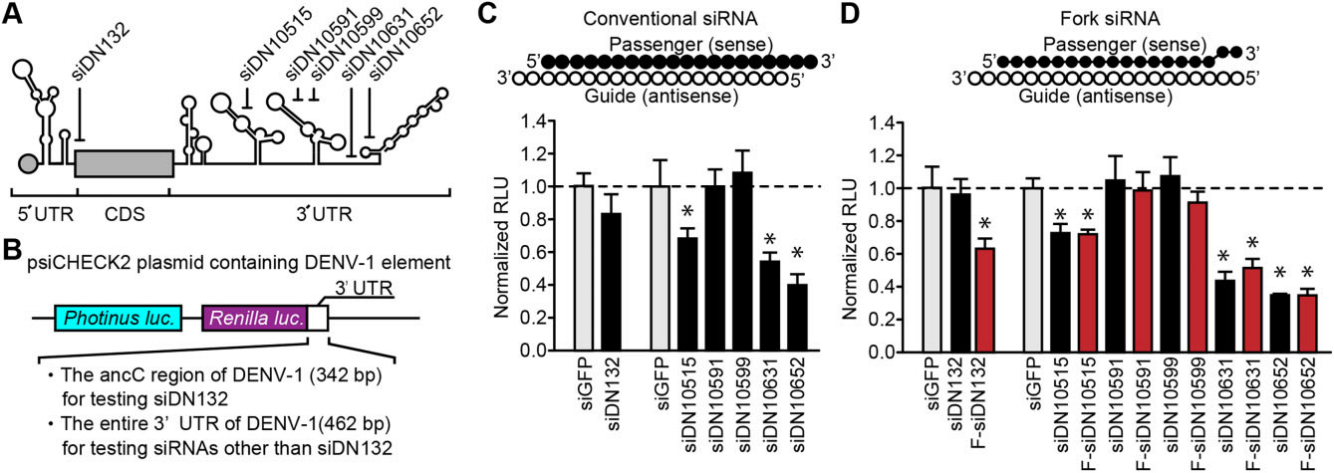
Assessment of siRNAs targeting DENV genome/derivative RNAs. (**A**) Schematic drawing of the DENV-1 genomic RNA and six siRNAs designed against it. The predicted secondary structure of the DENV-1 genomic RNA and corresponding positions of the six siRNAs are shown. (**B**) Construction of plasmid DNA for the reporter assay. The sequences of the ancC region or entire 3′-UTR in DENV-1 were inserted into the 3′-UTR of the *Renilla luciferase* gene of the psiCHECK2 vector for conventional dual reporter assay. (**C**) Assessment of the RNAi activity of the conventional siRNAs by a conventional reporter assay. Designed siRNAs were examined for their RNAi activity by the conventional reporter assay. An siRNA against green fluorescent protein (GFP), named siGFP, was used as a negative control. The target (*Renilla*) luciferase activity was normalized to control (*Photinus*) luciferase activity and further normalized to the data obtained from HEK293 cells co-transfected with each reporter plasmid and siGFP. Data represent the mean ± SD (*n* = 6). (**D**) Assessment of the RNAi activity of fork-siRNAs. The reporter assay was conducted as in (C). Data represent the mean ± SD (*n* = 6). *P<0.05 versus siGFP control.

The bar graphs are presented as means ± standard deviations (SDs). Statistical differences between each control and treatment group were examined using a one-way analysis of variance (ANOVA), followed by Dunnett’s tests for multiple comparisons in the data for Figures 1, 3, 4C and D, and by Tukey-Kramer test in the data for Figure 4E and F, respectively.

## Results

### Generation of siRNAs targeting all four DENV serotypes

To achieve DENV RNA genome silencing of all four serotypes by a single siRNA, consensus sequences were analyzed by multiple alignments. Conserved sequences of at least 18 consecutive nucleotides (nts) were identified at the beginning region of the open reading frame (ORF) and in the 3′-untranslated region (3′-UTR) of DENVs (Figure 1A). Six siRNAs were designed according to the sequences and examined their knockdown efficiency by a conventional reporter assay using the luciferase gene carrying 462 nt corresponding to the 3′-UTR of DENV-1 into the 3′-UTR of the reporter gene (Figure 1B). As shown in Figure 1C and Supplementary Table S1, siDN10652 exhibited the highest knockdown potency among designed siRNAs. Although fork-type siRNAs were designed for improving knockdown efficiency, no significant changes were observed (Figure 1D). Thus, siDN10652 was selected as the siRNA to silence the DENV genome RNA.

### SELEX using DENV-1-VLPs

**Figure 2.**
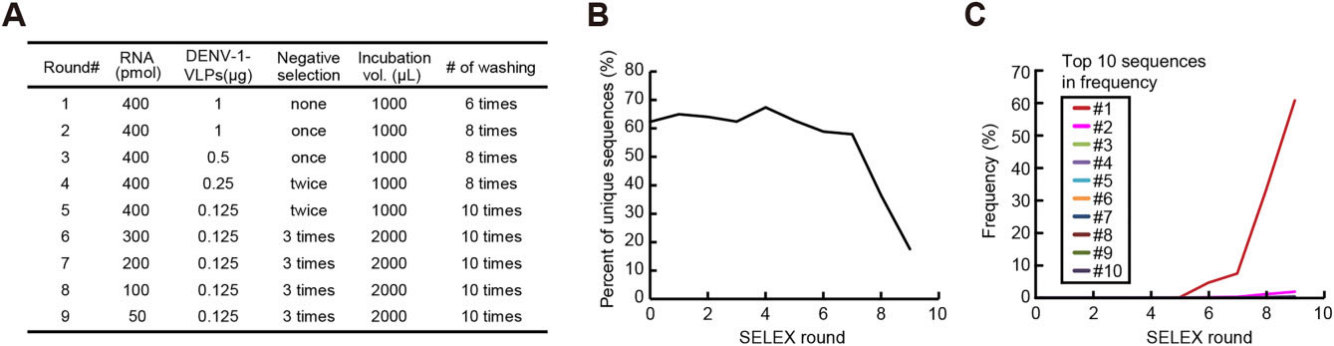
Library enrichment by VLP-SELEX. (**A**) SELEX condition. Conditions of VLP-SELEX in each round are shown. Negative selection was performed against an ultrafiltration column to remove non-specific molecules. (**B**) Library enrichment. Transition of library enrichment is shown by the percentage of number of unique sequences in every single SELEX round. (**C**) Sequence enrichment is shown as the frequency in percentage of the top 10 sequences in every single SELEX round.

Based on the conventional SELEX method, a SELEX system using VLPs (VLP-SELEX) was previously reported (11) and used here for identifying RNA aptamers against DENV-1-VLPs. After nine rounds of VLP-SELEX with DENV-1-VLPs as baits (Figure 2A), high-throughput sequencing and bioinformatics using RaptRanker (24) and FASTApatmer (23) revealed that library enrichment gradually started after round five and rapidly progressed after round seven (Figure 2B and Supplementary Table S2). The top 10 sequences in frequency (#1–10) differed by only two bases (insertions, deletions and substitutions), and the most frequent sequence accounted for over 60% of the library (Figure 2C), thereby indicating that only a single sequence and its derivatives were enriched in the library.

### Generation of RNA aptamers using DENV-VLPs

**Figure 3.**
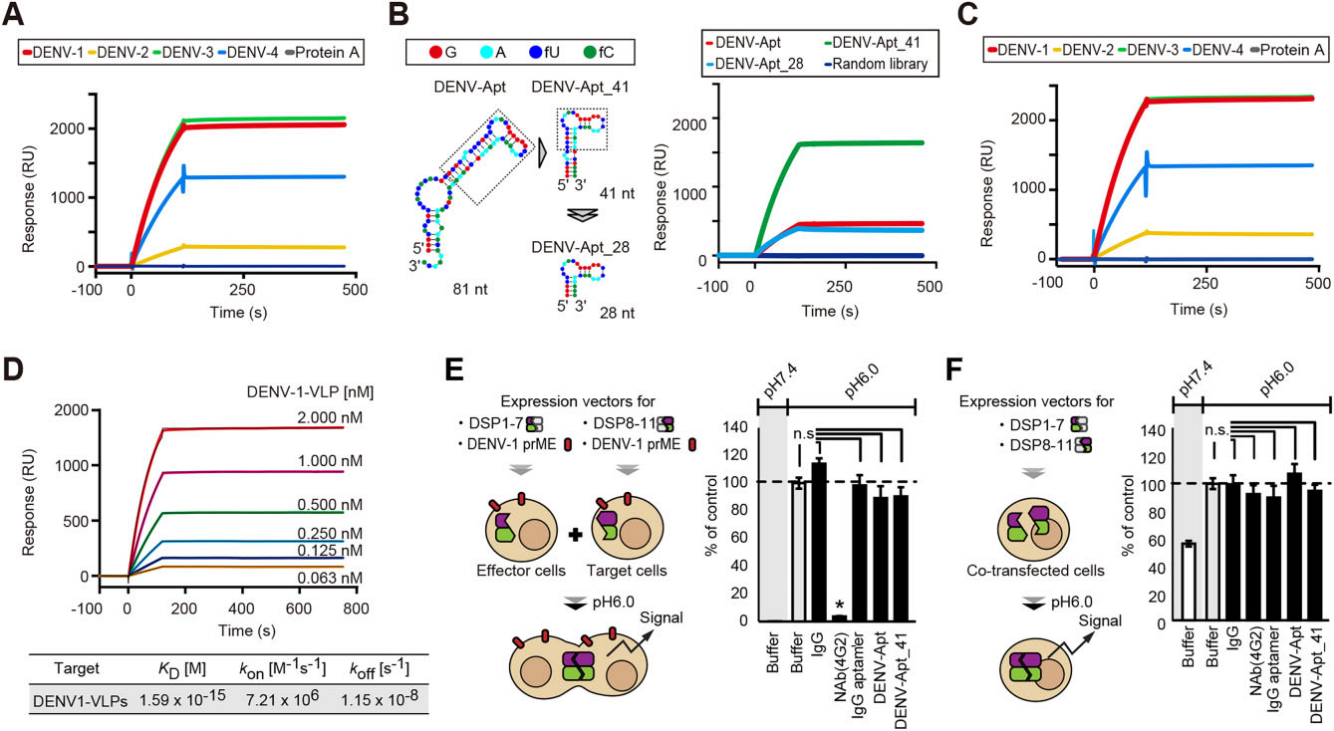
Characterization of an aptamer targeting DENV-VLPs. (**A**) SPR analysis of the affinity of an aptamer for each DENV-VLP serotype. Protein A was used as a negative control. All sensorgrams were normalized by subtraction of the response to random library immobilized in another flow cell. (**B**) Truncation of the aptamer. In the left panel, the predicted secondary structures of the original aptamer in 81 nt (DENV-Apt) and its truncated forms in 41 nt (DENV-Apt_41) and 28 nt (DENV-Apt_28) are shown. In the right panel, their binding ability to DENV-1-VLPs was examined by SPR analysis as in (A). (**C**) SPR analysis examining the affinity of DENV-Apt_41 to all DENV-VLP serotypes. (**D**) SPR analysis estimating the affinity parameters (*k*_on_, *k*_off_ and *K*_D_) of DENV-Apt_41. DENV-1-VLPs were injected at the indicated concentrations. (**E**) Membrane-fusion assay using a dual split protein reporter to estimate the effect of DENV-Apt_41 on viral membrane fusion. The assay was carried out at the indicated pH conditions. The luminescence derived from the *Renilla luciferase* reporter was measured, and the values were normalized to that of the cells without treatment (buffer) at pH 6.0, which was set as 100%. The 4G2 neutralizing antibody (Nab) clone and IgG were used as the positive control and its corresponding negative control, respectively. An IgG aptamer specific to IgG was used as a negative control of the aptamer. Data represent the mean ± SD (*n* = 3). (**F**) Specificity of the fusion assay. To evaluate the direct effect of the tested agents on the reporter activity, the agents examined in (E) were added to C6/36 cells co-expressing the split reporters, DSP1-7 and DSP8-11. Data represent the mean ± SD (*n* = 3). *P<0.05 versus buffer control at pH6.0; n.s, no significant difference between indicated groups.

SPR analysis revealed that the most frequent sequence had a high affinity for DENV-1-VLPs (Figure 3A and Table 1). Furthermore, this RNA aptamer showed high affinity not only to the DENV-1-VLPs used in VLP-SELEX but also to the other serotypes of DENV-VLPs, indicating that the aptamer had a broad binding ability for DENV-VLPs (Figure 3A). Moreover, when this aptamer, named DENV-Apt, was shortened to 41 nt (DENV-Apt_41) and 28 nt (DENV-Apt_28) to identify the core sequences conferring affinity to the target, DENV-Apt_41 showed higher binding response to DENV-1-VLPs than the full-length aptamer DENV-Apt (Figure 3B) and retained a broad affinity to all DENV-VLP serotypes (Figure 3C). Although accurate affinity parameters of DENV-Apt_41 for VLPs were not calculated because of unknown valency and number of binding sites in the SPR analysis, the dissociation constant was roughly estimated and is shown as a reference value (Figure 3D).

**Table 1.** Sequences of DENV-Apt and its truncated aptamers targeting DENVs

Name	Length (nt)	Sequence (5' to 3')*
DENV-Apt	81	GGGUCUUGUUUCUUCUCUGUGUGUAAUUUGUUUUACUGGGGGGUUAACCUAACAAGUGCACACUCGUCACACACUCACAUC
DENV-Apt_41	41	GGGUAAUUUGUUUUACUGGGGGGUUAACCUAACAAGUGCCC
DENV-Apt_28	28	GGGUUUUACUGGGGGGUUAACCUAACCC

*These aptamers were transcriptionally generated using 2′-fluoro-CTP, 2′-fluoro-UTP, ATP and GTP.

To estimate the effect of DENV-Apt_41 on DENV infection, a membrane-fusion assay system involving DENV E proteins based on the DSP was carried out as previously reported (27). As shown in Figure 3E, the well-known 4G2 neutralizing antibody clone against DENVs, which was used as a positive control, significantly suppressed luciferase signals induced by membrane fusion. In contrast, DENV-Apt_41 and its parent sequence did not suppress cell–cell membrane fusion, even at concentrations as high as 1000 nM. In contrast, a control assessment in DSP expressing cells by co-transfection of each DSP showed no changes in the signals relative to the buffer control, suggesting a specific membrane fusion inhibition effect of 4G2 NAb (Figure 3F). In addition, competitive binding analyses with 4G2 clone targeting the fusion loop in the E protein of DENVs were carried out in the SPR assay, and consequently competitiveness between DENV-Apt_41 and 4G2 NAb was not observed (Supplementary Figure S1). These results suggested that DENV-Apt_41 can bind to all four serotypes of DENVs with high affinity, but does not affect DENV fusion. Thus, DENV-Apt_41 was considered useful as a delivery tool in chimeric RNA for evaluating the effectiveness of the RNA interference (RNAi) strategy.

### Conjugation of siDN10652 and DENV-Apt_41 as a chimera

**Figure 4.**
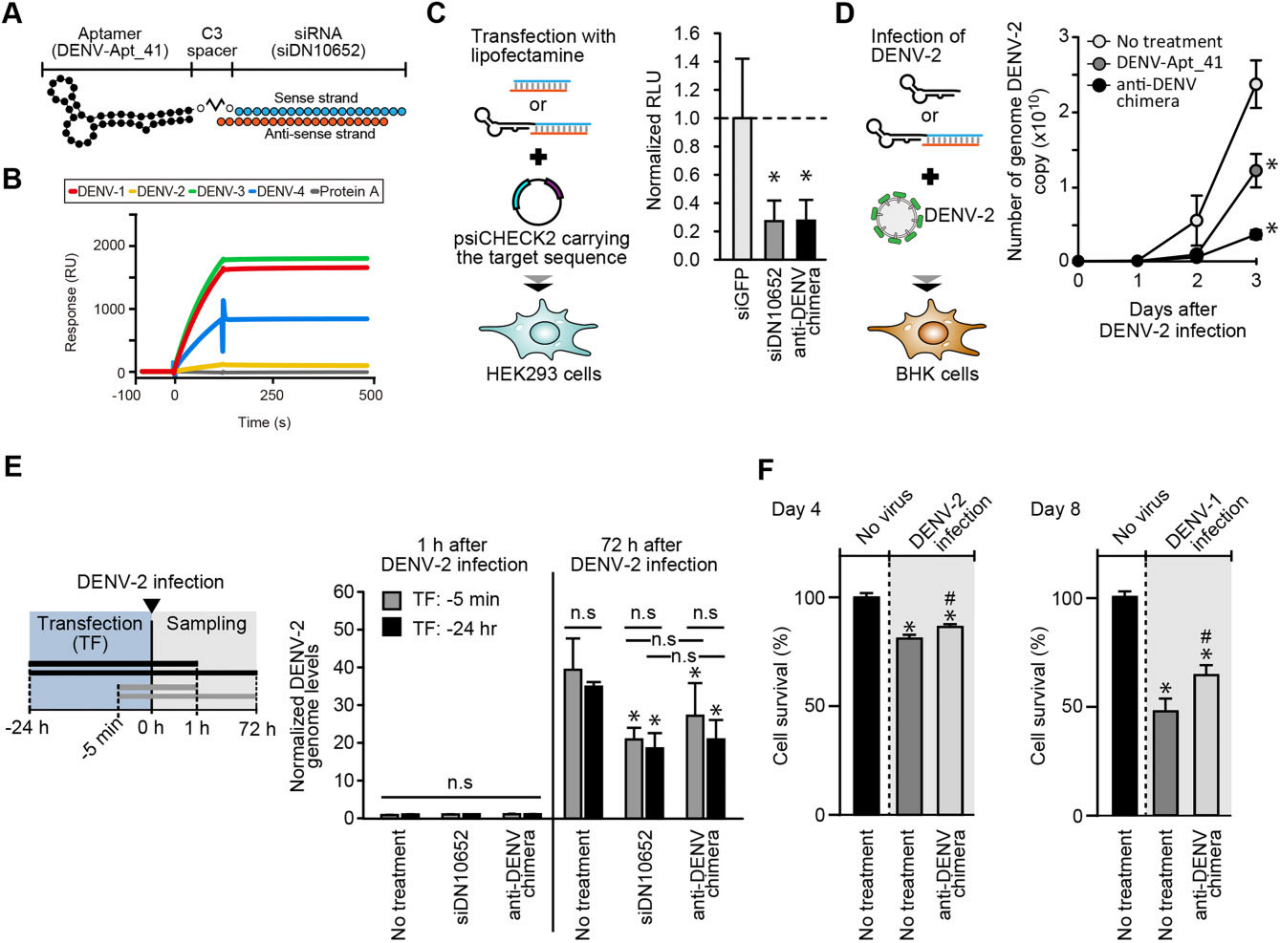
Anti-DENV chimeric RNA. (**A**) Schematic drawing of the anti-DENV chimeric RNA. DENV-Apt_41 was conjugated with the sense strand of siDN10652 via a C3 spacer. (**B**) Binding ability of anti-DENV Chimeric RNA to DENV-VLPs. The affinity of the chimera to all four serotypes of DENV-VLPs was examined. The chimera was labeled with biotin at the 5′-end of the aptamer part and immobilized on an SA sensor chip. (**C**) RNAi activity of the chimera. The knockdown efficiency of the chimeric RNA was examined by co-transfection with the reporter plasmid DNA and the chimera in HEK293 cells. The siGFP and siDN10652 were used as negative and positive controls, respectively. Data represent the mean ± SD (*n* = 8). *P<0.05 versus siGFP-transfected control. (**D**) Effect of anti-DENV chimeric RNA in DENV-2 infection assay. DENV-2 was mixed with the chimera or DENV-Apt_41, and BHK cells were then exposed to the mixtures. The effect of the chimera and DENV-Apt_41 at a final concentration of 500 nM on DENV-2 genome replication was examined by PCR at the indicated time points. Data represent the mean ± SD (*n* = 2). *P<0.05 versus no treatment group at day 3. (**E**) Effect of lipofection-induced siDN10652 and anti-DENV chimeric RNA on DENV-2 infection. Experimental procedures were shown in left panel and inhibition effects of lipofection-induced siDN10652 and DENV-Apt_41 at a final concentration of 500 nM on DENV-2 genome replication was examined by PCR as in (D) (right panel). Data represent the mean ± SD (*n* = 3). *P<0.05 versus no treatment group at 1 or 72 h, respectively. No significant differences (n.s) were shown between indicated pairs. (**F**) Effect of anti-DENV chimeric RNA in MNT with DENV-1 and DENV-2. MNT was performed at day 4 in DENV-2 (left) and at day 8 in DENV-1 (right) at a final concentration of 500 nM of anti-DENV chimeric RNA. Data represent the mean ± SD (*n* = 3). *P<0.05 versus no treatment group without virus. #*P*<0.05 versus no treatment group with virus.

Based on the results for siRNAs and RNA aptamers, a chimeric RNA was generated by the conjugation of siDN10652 and DENV-Apt_41. As shown in Figure 4A, DENV-Apt_41 was covalently conjugated to the 5′ end of the passenger (sense) strand of siDN10652 via a C3 spacer so as not to affect RNAi activity because the guide (antisense) is loaded into RNA-mediated silencing complex (RISC). This complex, named anti-DENV chimeric RNA, was firstly subjected to SPR analysis to examine active function as the aptamer part (Figure 4B). This result clearly demonstrated that anti-DENV chimeric RNA retained the binding ability of DENV-Apt_41 to all DENV-VLP serotypes even in the conjugated form. In contrast, to examine RNAi activity in the reporter assay described above, anti-DENV chimeric RNA was co-transfected with the reporter into HEK293 cells by lipofection. The RNAi knockdown efficiency of the chimera was equivalent to that of siDN10652 (Figure 4C). These results indicated that the anti-DENV chimeric RNA possessed the ability of each functional RNA modality without losing activity.

### Effect of the anti-DENV chimeric RNA on DENV-2 challenge *in vitro*

After evaluation of each function in anti-DENV chimeric RNA, the chimera was lastly assessed in a genuine DENV-2 challenge *in vitro*. DENV-2 was mixed with the chimera or DENV-Apt_41 (negative control), and BHK cells were then exposed to the mixtures. PCR analysis was conducted to examine the changes in the copy number of the DENV genomic RNA (29,30). In the time-course analysis, DENV-Apt_41 unexpectedly reduced DENV-2 replication by ∼50% on day 3, whereas the anti-DENV chimeric RNA suppressed it by ∼90% (Figure 4D). This result suggested that DENV-Apt_41 inhibits DENV-2 replication by affecting infection processes other than membrane fusion. Even accounting for this, however, the anti-DENV chimeric RNA was more effective than the aptamer alone. Further, other oligonucleotides, such as siGFP and a random sequence as a negative control for aptamer, were affected efficiency of DENV-2 replication and/or infection, but no significant differences with the virus-treated-alone control group were observed, except for DENV-Apt_41 and anti-DENV chimeric RNA (Supplementary Figure S3). In addition, as a representative delivery method for oligonucleotides, a transfection strategy using liposome was also effective for suppression of DENV-2 replication, but its efficiency was 50% or less, even in a prophylactic treatment of oligonucleotides 24 h prior to DENV-2 infection (Figure 4E).

Finally, to confirm effect of anti-DENV chimeric RNA other than a criterion of RNA levels of DENV genome, micro-neutralization test (MNT) based on the cell survival rate was performed using not only DENV-2 but also another serotype DENV-1 (28). These results showed that the treatment of anti-DENV chimeric RNA significantly improved viability of DENV-2- and DENV-1-infected BHK cells (Figure 4F). A series of results indicated that anti-DENV chimeric RNA has neutralizing activity against multiple serotypes of DENV, at least DENV-1 and DENV-2, without the aid of any other delivery strategies such as liposome.

## Discussion

Conventional therapeutic approaches against DENV have severe limitations. Therefore, new therapeutic modalities must be investigated. A chimeric RNA consisting of an RNA aptamer-conjugated siRNA has previously been tested as an antiviral agent against HIV-1. However, the effectiveness of chimeric RNA remains unclear owing to an insufficient number of practical evaluations. Here, we investigated whether chimeric RNA could be used as a treatment modality against DENV. We generated siRNAs and aptamers targeting all four DENV serotypes to degrade DENV-derived RNAs and bind to DENV particles, respectively. Furthermore, their conjugated form, an anti-DENV chimeric RNA, suppressed the *in vitro* production of DENV-1 and DENV-2, suggesting the effectiveness of aptamer-based intracellular siRNA delivery, which was presumably achieved by co-entry with the virus.

Our results indicated that DENV-Apt_41 inhibits DENV-2 replication by 50%. DENV-Apt_41 did not affect the fusion process (Figure 3E, F, and Supplementary Figure S1) but inhibited DENV-2 replication, possibly owing to the inhibition of viral attachment. Thus, this aptamer may potentially be used as a DENV inhibitor, although its inhibitory effect appears to be weak in practical applications. As for the affinity of DENV-Apt_41 to DENV-VLPs, several affinity indices (*k*_on_, *k*_off_ and *K*_D_) of the aptamer were estimated using SPR analysis, resulting in femto-molar *K*_D_ value in the univalent fitting model. However, this value should be re-evaluated in a multivalent fitting model, which is difficult as it seems unfeasible to reveal the actual binding mode of aptamers and DENV-VLPs on the sensor chip by SPR analysis. To accurately estimate the affinity parameters of the aptamer, SPR analysis was performed using recombinant E proteins derived from DENV-3 (Supplementary Figure S2). However, an interaction between them was not observed, possibly because of the difference in the three-dimensional structure of epitopes between recombinant proteins and VLPs. Despite several challenges in the characterization of aptamers targeting virus particles, the suppressive effect of anti-DENV chimeric RNA on DENV replications is clearly superiors to that of DENV-Apt_41 alone; thus, DENV-Apt_41 appears to play a key role in chimeric RNA as a delivery platform. Further, there was a certain difference in the affinity of DENV-Apt_41 (or anti-DENV chimeric RNA) to each serotype of DENV-VLPs. DENV-Apt_41 showed a high affinity to DENV-1- and DENV-3-VLPs, a moderate affinity to DENV-4-VLPs, and a weak affinity to DENV-2-VLPs. The high affinity of DENV-Apt_41 to DENV-1-VLPs appear to be reasonable because VLP-SELEX was carried out using DENV-1-VLPs as a screening bait. Moreover, an alignment analysis among DENV four serotypes showed a similarity between DENV-1 and DENV-3, and hence the high affinity of DENV-Apt_41 to DENV-3 appears to make sense. However, regarding the other serotypes, there was no correlation between the affinity of the aptamer and a similarity of the primary amino acid sequences of E protein in all four serotypes (Supplementary Figure S4). Therefore, one hypothesis is that this aptamer may have a high affinity for amino acid sequences that differs only in DNEV-2 and is common for the other three serotypes of DENVs (Supplementary Figure S4B and C). Taken together, the affinity of DENV-Apt_41 to each DENV serotype may not to simply rely on the homology of envelope proteins among the serotypes, and hence further analyses including structural analysis are needed to understand difference of the affinity of DENV-Apt_41 to each serotype.

Previous reports for oligonucleotide therapies targeting viruses have mentioned the potential of chimeric RNA molecules consisting of siRNA/antisense and aptamers (17). However, the target virus of chimeric RNAs was limited to HIV-1 (17), and thus the effectiveness of the strategy against other viruses was yet to be evaluated. In this study, it was suggested that chimeric RNA strategy is equivalent or superior to a lipofection strategy in the siRNA delivery method as a novel viral therapy (Figure 4E). In addition, our study provides another practical example targeting a different virus, and the results indicate the effectiveness of the chimeric RNA strategy, in which viral RNAs are degraded by siRNAs that are presumably internalized into the cells via aptamer-mediated delivery. To acerate evaluation of therapeutic strategy using chimeric RNAs against viruses, generation of aptamers against viral particles may be a key bottleneck. In recent years, several antiviral aptamers have been created using recombinant proteins, particularly spike protein of SARS-CoV-2 (33), but their number remains limited. The generation of aptamers targeting viral particles may be an important bottleneck. To improve such a situation, we have been focusing on membrane proteins as an aptamer target (34,35) and established SELEX methods such as Icell-SELEX with isogenic cultured cells (26) and VLP-SELEX with VLPs (11). The current study provides insights into the usefulness of VLP-SELEX again for generating chimeric RNA. In addition to our SELEX methods, an establishment of advanced methods to generate aptamers against cell-surface proteins may accelerate the generation and assessment of chimeric RNA and other modalities that can target different contexts such as viral infections or cancer.

As a limitation of this study, the effect of dual inhibition by the siRNA and aptamer has not been investigated. To address this, we are currently committed to generating RNA aptamers with a high-neutralizing activity as stand-alone inhibitors and/or an essential part of chimeric RNA conferring dual inhibition. The work will be reported in another study in the future. Regarding gene silencing, we designed and used siRNAs targeting consensus sequences in all four DENV serotypes for the knockdown of every DENV serotype, but the silencing efficiency of the siRNA was only ∼60–80% in the reporter assay. Other gene silencing modalities, such as antisense, may achieve potent knockdown of DENV genome RNAs through silencing mechanisms different from those of siRNAs, although these modalities target the same limited consensus sequences. Further, the silencing effects of the anti-DENV chimeric RNA should be confirmed against other serotypes of authentic DENV other than DENV-2 and DENV-1. These approaches should be addressed in future studies to assess and understand the potential of chimeric RNA. In contrast, similar to previous studies of RNA chimera targeting HIV-1 (21,22), the mechanism of endosome escape of the chimeric RNA remains unknown. Studies for uncovering these mechanisms are essential for developing more efficient delivery systems for various drug modalities. Furthermore, *in vivo* studies are essential to assess whether chimeric RNAs are suitable for prophylactic or therapeutic treatment.

In conclusion, we demonstrated that our anti-DENV chimeric RNA is an effective strategy for the inhibition of DENV-1 and DENV-2 production *in vitro*, suggesting the broad utility of chimeric RNA-based viral treatment. The virus- and cell-specific aptamer-based delivery of oligonucleotide/other drug modalities may potentially be used as targeted therapy for various diseases.

## Supplementary Material

ugae025_Supplemental_File

## Data Availability

All relevant data are presented here or in Supplementary Data.
