# Supplementary material for: A chimeric RNA consisting of siRNA and aptamer for inhibiting dengue virus replication
Source: NAR Mol Med. 2024 Dec 25;1(4):ugae025. doi: 10.1093/narmme/ugae025 (PMC12429950; doi:10.1093/narmme/ugae025)
Supplement: ugae025_Supplemental_File [file ugae025_Supplemental_File.pdf]

Supplementary Information for

A chimeric RNA consisting of siRNA and aptamer for inhibiting dengue virus replication.

Ryo Amano, Masaki Takahashi, Kazumi Haga, Mizuki Yamamoto, Kaku Goto, Ichinose Akiko, Michiaki Hamada, Jin Gohda, Jun-ichiro Inoue, Yasushi Kawaguchi, Meng Ling Moi, Yoshikazu Nakamura

Correspondence: Masaki Takahashi, Project Division of RNA Medical Science, The Institute of Medical Science, The University of Tokyo, Minato-ku, Tokyo 108-8639, Japan. Phone, +81-3-5449-5324.
E-mail, tmasaki@ims.u-tokyo.ac.jp

Supplementary data includes:

- Figures S1 to S4
- Tables S1 to S2
- Note S1

Supplementary Figures

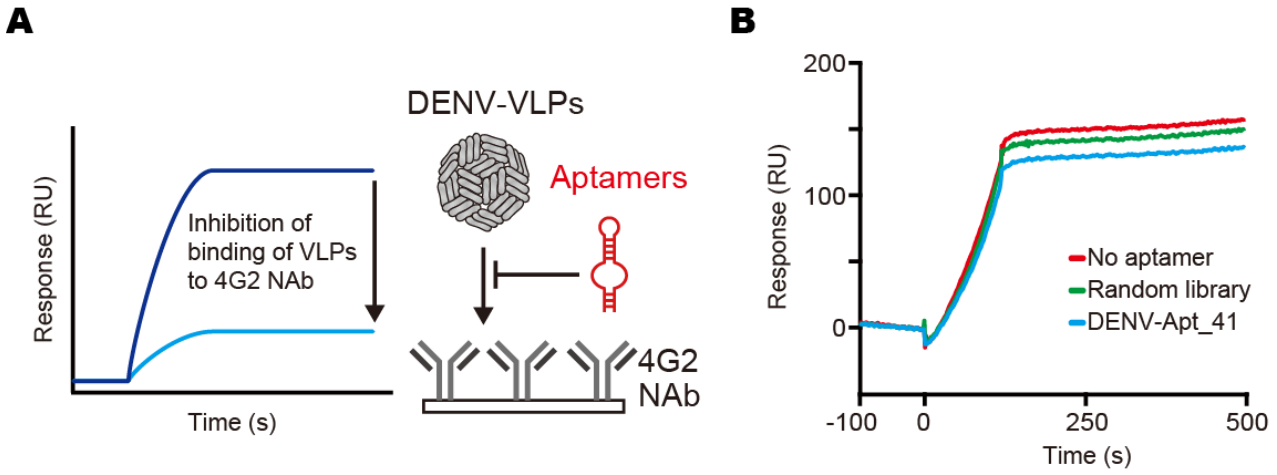

Figure S1. SPR analysis for competitive activity of the aptamer to neutralizing antibody 4G2. (**A**) Schematic drawing of a competition assay in SPR analysis. To estimate effect of DENV-Apt_41 on fusion process of DENVs, 4G2 neutralizing antibody (4G2 Nab) was immobilized onto a CM5 sensor chip mediating a protein A, and then mixture of DENV-1-VLPs and tested sequences was injected. (**B**) Sensorgrams of competition assay in SRP analysis. DENV-1-VLPs were mixed with DENV-Apt_41, a random library and buffer (no aptamer) as negative controls, and the mixtures was examined in SPR analysis using 4G2 Nab immobilized sensor chip.

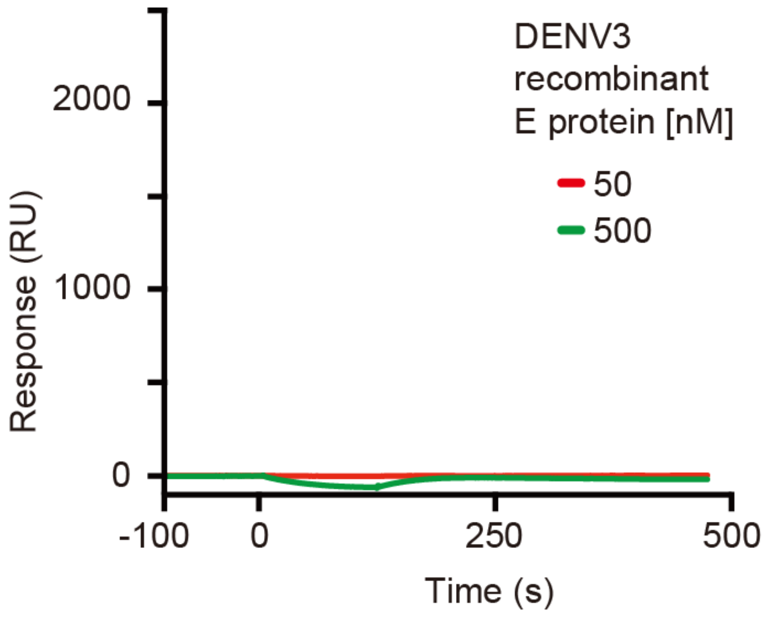

Figure S2. Interaction of DENV-Apt_41 with recombinant E protein of DENV-3 in SPR analysis. To estimate accurate affinity parameters (K_{on}, K_{off}, and K_D values) of DENV-Apt_41 to DENV relevant proteins, recombinant envelope protein of DENV-3 was subjected to SPR analysis using DENV-Apt_41 immobilized sensor chip. Even E proteins at concentrations of as high as 500 nM did not show interaction with DENV-Apt_41.

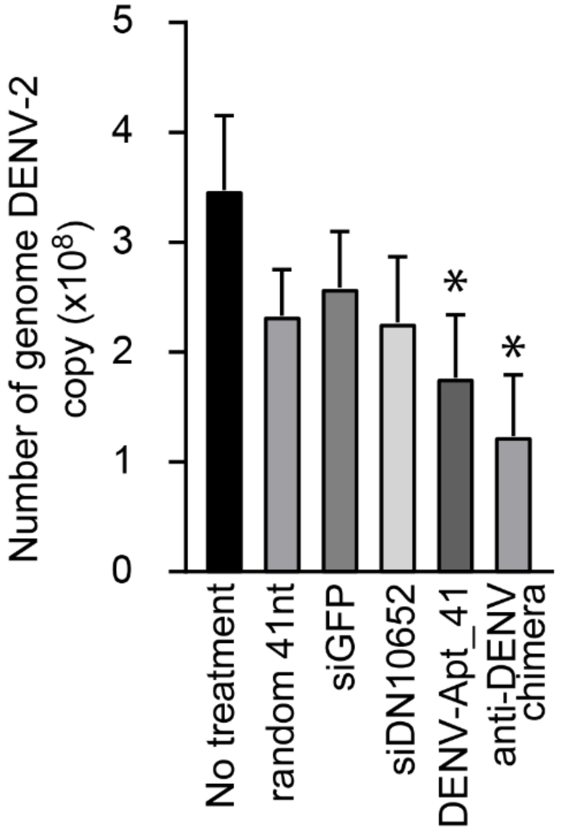

Figure S3. Effect of anti-DENV chimeric RNA and the other oligonucleotides on DENV-2 infection assay. DENV-2 was mixed with indicated oligonucleotides including the chimera and DENV-Apt_41, and BHK cells were then exposed to the mixtures as in figure 3. The effect of the oligonucleotides at a final concentration of 500 nM on DENV-2 genome replication was examined by PCR at day 3. Data represent the mean ± SD (n = 3). * $p<0.05$ versus no treatment group, which was exposed to only DENV-2.

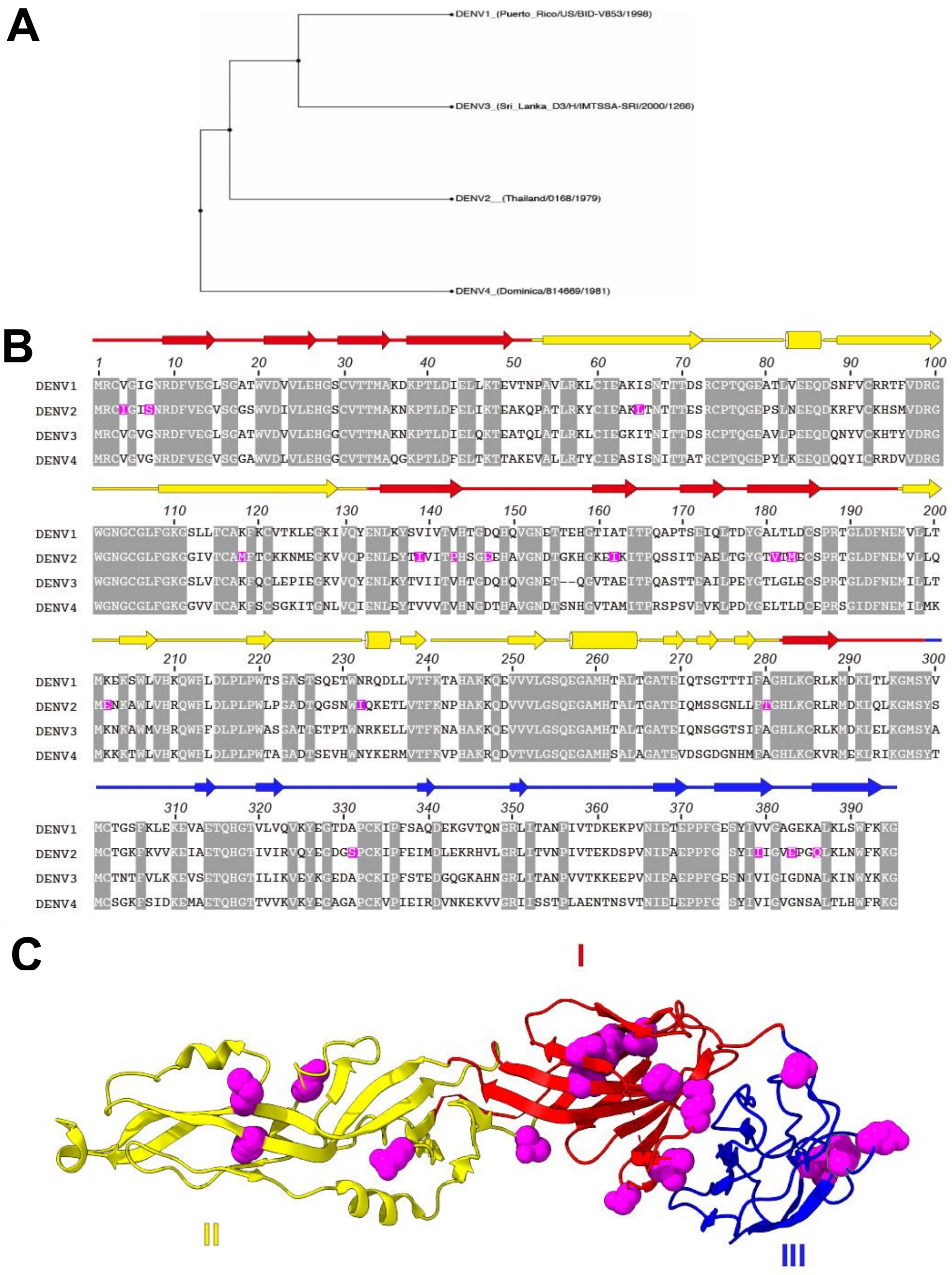

Figure S4. Sequence alignment. (**A**) Hierarchical tree. Similarity and distance of amino acid sequences of DENV-1 to -4 were expressed as a hierarchical tree. (**B**) Multiple sequence alignment of the ectodomain (1 to 395 residues) of the E protein from four serotypes of DENV. Residue numbers correspond to the complete DENV E protein. Domains I, II and. III are indicated by red, yellow and blue bar, respectively. Secondary structures are shown above the sequences. Arrows and cylinders indicate β-strands and helices, respectively. Fully conserved amino acid residues are highlighted with gray boxes. Amino acid residues that are not conserved in only DENV-2 are highlighted with magenta boxes. (**C**) Mapping of the magenta-colored residues in (B) to the tertiary structure of the ectodomain of the DENV-2 E protein (PDB ID: 3C6E). The tertiary structure is color-coded to the corresponding domains on the secondary structure in (B).

Supplementary Tables

Table S1. Synthetic siRNA sequences targeting DENVs

Name	Sense stand (passenger)	Antisense strand (guide)	Feature of targeted sequence
siGFP	CAGCACGACUUCUUCAAGUU	CUUGAAGAAGUCGUGCUGUU	GFP
siDN132	UCAAUAUGCUGAAACGCGUU	CGCGUUUCAGCAUAUUGAUU	Anchored capsid protein ancC
siDN10515	GGUUAGAGGAGACCCCUUU	AGGGGUCUCCUCUAACCUU	xrRNA3(DBI)
siDN10591	GGACUAGAGGUUACAGGAUU	UCCUCUAACCUCUAGUCCUU	xrRNA4(DBII)
siDN10599	GGUUAGAGGAGACCCCCCUU	GGGGGGUCUCCUCUAACCUU	xrRNA4(DBII)
siDN10631	GCAUAUUGACGCUGGGAUU	UCCCAGCGUCAAUAUGCUU	Cyclization sequence
siDN10652	GACCAGAGAUCCUGCUGUUU	ACAGCAGGAUCUCUGGUCUU	Hairpin1
F-siDN132	UCAAUAUGCUGAAACGAU	CGCGUUUCAGCAUAUUGAUU	Anchored capsid protein ancC
F-siDN10591	GGACUAGAGGUUAGAGUU	UCCUCUAACCUCUAGUCCUU	xrRNA3(DBI)
F-siDN10599	GGUUAGAGGAGACCCCAA	GGGGGGUCUCCUCUAACCUU	xrRNA4(DBII)
F-siDN10515	GGUUAGAGGAGACCCAA	AGGGGUCUCCUCUAACCUU	xrRNA4(DBII)
F-siDN10631	GCAUAUUGACGCUGGUU	UCCCAGCGUCAAUAUGCUU	Cyclization sequence
F-siDN10652	GACCAGAGAUCCUGCUUA	ACAGCAGGAUCUCUGGUCUU	Hairpin1

Table S2. Sequence data analyzed with FASTAptamer (1/5)

Name[*1]	Sequence (5' to 3')	Length[*2]	Rank[*3]	Reads[*4]	RPM[*5]	Cluster[*6]	Rank in Cluster[*7]	Edit Distance[*8]
DENV-Apt	GGGTCTTGTTTCTTCTCTGTGTGTAATTTGTTTTACTGGGGGGTTAACCTAACAAGTGCACACTCGTCACACACTCACATC	81	1	40586	568049.49	1	1	0
	GGGTCTTGTTTCTTCTCTGTGTGTAATTTGTTTTACTGGGGGGTTAACCTAACTCGTCACACACTCACATC	71	46	39	545.85	2	1	0
	GGCGACTATAACCTTAAGTTTATGAAATTTATCAAGATCAATTTCTGCTGTGGGTTCCATGTCATTGGA	69	54	32	447.88	3	1	0
	GGGTCTTGTTTCTTCTCTGTGAATAGCCAACATGAATACGTACTGACAAACTGTGGATACGACTCGTCACACACTCACATC	81	70	23	321.91	4	1	0
	GGGTCTTGTTTCTTCTCTGTGCTAATTACGATCAACTAGGATACGCTCCAGATGTGAAAAACTCGTCACACACTCACATC	80	76	20	279.92	5	1	0
	GGGTCTTGTTTCTTCTCTGTGGCAACTAATACGACAAACAAACGACGACAGTATACGATTGACTCGTCACACACTCACATC	81	79	19	265.93	6	1	0
	GGCGACTATAACCTTTTCCATTATATCAAGAACTAGTGGGATTAGTGTTGGGCGTTCCATGTCATTGGA	69	79	19	265.93	7	1	0
	GGGTCTTGTTTCTTCTCTGTGTTAGAAACCTTGCGACTGACTGTTAATGACTGATACGGACTACTCGTCACACACTCACATC	82	82	18	251.93	8	1	0
	GGACTCACAGCTCAGGGTACAGGCCACGACTGTCCGCCGCTCTAAGCGTGGGGGAACCCCATGCGAGTGCAGGTG	75	90	16	223.94	9	1	0
	GGGTCTTGTTTCTTCTCTGTGCTCGATAAAAGAAAAAGTTCCCCACGAAAGGTAAAAGGCTAACTCGTCACACACTCACATC	82	90	16	223.94	10	1	0
	GGGTCTTGTTTCTTCTCTGTGTTCTCTAATACAACTCACTGGATCCGCGGCTTGCATAAATACTCGTCACACACTCACATC	81	96	15	209.94	11	1	0
	GGGTCTCGTTCTTCTC	16	102	14	195.95	12	1	0
	GGCGACTATAACCTCATGTTAAAACGACTAGCAAGAAATCGTTTATGCGTGTGGTTCCATGTCATTGGA	69	102	14	195.95	13	1	0
	GGGTCTTGTTTCTTCTCTGTGTAGGTTAATACGACACTATATCCTGCTTGCTGGGGAAACTCGTCACACACTCACATC	78	106	13	181.95	14	1	0
	GAGTCTTGTTTCTTCTCTGTGCGTCAACTAATATGACTAACTATAAAGTTATGCGCTTCGACACTCGTCACACACTCACATC	82	114	12	167.95	15	1	0
	GGGTCTTGTTTCTTCTCTGTGGTAAGGACTAATTGGTAACGCCACTATCGTTACTAATACCACTCGTCACACACTCACATC	81	114	12	167.95	16	1	0
	GGGTCTTGTTTCTTCTCTGTGGGAGTGCGACTCACTATCGATGATCACTATATAAACATGACTCGTCACACACTCACATC	80	114	12	167.95	17	1	0
	GGCGACTATAACCTAGCAAATTCTCATATTAAGAACTGGGCATTTGTGGTGGTTCCATGTCATTGGA	67	114	12	167.95	18	1	0
	GGCGACTATAACCTCAGTCCAAGTTGTATTAAGATTCATTTGCTTGTTTTGCGGTTCCATGTCATTGGA	69	123	11	153.96	19	1	0
	GGGTCTTGTTTCTTCTCTGTGTGTACTTAATCATCGACTTACCTTACAACTCACCGATCTCACTCGTCACACACTCACATC	81	123	11	153.96	20	1	0
	GGACTCACAGCTCAGGGATGCGTATAGAGATTGAAAGAAAACGGTATCGGGAACCCCCCCATGCGAGTGCAGGTG	75	123	11	153.96	21	1	0
	GGGTCTTGTTTCTTCTCTGTGCCGGGATATGACTCACAATTTTACAACTTTCCTTCACGTTACTCGTCACACACTCACATC	81	123	11	153.96	22	1	0
	GGGTCTTGTTTCTTCTCTGTGCAAAACCCGGTTGTAATGCGAACTCACTGTAGATGCGAACTCGTCACACACTCACATC	79	123	11	153.96	23	1	0
	GGCGACTATAACCTTGGCGGCTTTAAGATAATCAAGAAAATCTTGCGGCTGGGGTTCCATGTCATTGGA	69	123	11	153.96	24	1	0
	GGCGACTATAACCTACGAAATCCGTTACTATCAAGAATTAACGGTATTGTGCGGTTCCATGTCATTGGA	69	134	10	139.96	25	1	0
	GGCGACTATAACCTTTATTTGCACTTTAAAAGTAGGGAGTGCATTGTTTTGGCATTCCATGTCATTGGA	69	134	10	139.96	26	1	0
	GGCGACTATAACCTTTGATCCTATTCTTAAAAGTAGTGGAATTGGGATACGGGGTTCCATGTCATTGGA	69	134	10	139.96	27	1	0
	GGGTCTTGTTTCTTCTCTGTGTGCAAGTTTATACCCATCCCTGATCTCACTAACAAATACGACTCGTCACACACTCACATC	81	134	10	139.96	28	1	0
	GGGTCTTGTTTCTTCTCTGTGAAGAGCACATACGGCGACTATGACATACAACTCACTGAAAACTCGTCACACACTCACATC	81	134	10	139.96	29	1	0
	GGGTCTTGTTTCTTCTCTGTGGCTATATACGACACGACTGACTATCTAAAGCTGCTCACGAACTCGTCACACACTCACATC	81	134	10	139.96	30	1	0
	GGGTCTTGTTTCTTCTCTGTGATGCTAATACGAACTATATCGACTCGCTAATGCGATACTCACTCGTCACACACTCACATC	81	134	10	139.96	31	1	0
	GGGTCTTGTTTCTTCTCTGTGTAATGAACGACTCACAACTAGGAACTCGACTCAGATGTGAACTCGTCACACACTCACATC	81	134	10	139.96	32	1	0
	GGCGACTATAACCTCGCAAGAATTGTTGCTATAAAGATGTACTTTCATGCGGGGTTCCATGTCATTGGA	69	152	9	125.97	33	1	0
	GGCGACTATAACCTAACGTCTGCTATAAAGATTGCGAGATGGGTATATAGTGCGTTCCATGTCATTGGA	69	152	9	125.97	34	1	0
	GGGTCTTGTTTCTTCTCTGTGGGCTTAATACGGCTTGCAATACATACTCACTTAATACACTACTCGTCACACACTCACATC	81	152	9	125.97	35	1	0
	GGGTCTTGTTTCTTCTCTGTGGCTAATCGACTTAACTAGGCATACGCGACCATACTATGACACTCGTCACACACTCACATC	81	152	9	125.97	36	1	0
	GGGTCTTGTTTCTTCTCTGTGTGTCACTAGTGACCCTTCCTTGCTGGTGTATACTGATACGACTCGTCACACACTCACATC	81	152	9	125.97	37	1	0
	GGGTCTTGTTTCTTCTCTGTGAATATTACTCACTCCTTACGCCGATTACCTTGAGTCAAAACTCGTCACACACTCACATC	80	152	9	125.97	38	1	0
	GGGTCTTGTTTCTTCTCTGTGTCTTCAAGACTAATATCGACATTCTCACTCATGACTCACTACTCGTCACACACTCACATC	81	152	9	125.97	39	1	0
	GGCGACTATAACCTTTAAACACGGTTTTATAAAGAACAAACTGAGTTATGTGGGTTCCATGTCATTGGA	69	173	8	111.97	40	1	0
	GGGTCTTGTTTCTTCTCTGTGCACACAATATTACTCACTTCGCGACGCACATGCATACTCGTCACACACTCACATC	76	173	8	111.97	41	1	0
	GGGTCTTGTTTCTTCTCTGTGATCCACTATATCATGCATGTACGATGCCCTAAAGGTTACGACTCGTCACACACTCACATC	81	173	8	111.97	42	1	0
	GGGTCTTGTTTCTTCTCTGTGTGTCAAGCCTCCTTTTAACTGCGACTTACTGATCTGGCTCACTCGTCACACACTCACATC	81	173	8	111.97	43	1	0
	GGCGACTATAACCTGCTATTAAGAACCAGGTGCTTAGTCGGTGCGTGGAGTGGGTTCCATGTCATTGGA	69	173	8	111.97	44	1	0
	GGGTCTTGTTTCTTCTCTGTGTCGCTGCCGTTAGTGGAGTCGAATCTCGAATGGGACATTAACTCGTCACACACTCACATC	81	173	8	111.97	45	1	0
	GGGTCTTGTTTCTTCTCTGTGCCATCATCGTAGCGACTCATAATACGACAATCTCATCTCACTCGTCACACACTCACATC	80	173	8	111.97	46	1	0
	GGGTCTTGTTTCTTCTCTGTGCTACATATGACTCAAAACGTCTCACGAAAGGTATATCGACACTCGTCACACACTCACATC	81	173	8	111.97	47	1	0
	GGGTCTTGTTTCTTCTCTGTGTTTGGAATAGACCTCGAAGACCGAATTTCACTCTTACAAAACTCGTCACACACTCACATC	81	173	8	111.97	48	1	0
	GGCGACTATAACCTAGTGTGCCAGGAACAATGCGGCGTCAAGTAGCTTGCACGGTTCCATGTCATTGGA	69	173	8	111.97	49	1	0
	GGGTCTTGTTTCTTCTCTGTGTATAACTGAAATACGACCCACGGTAATAACGACTCTAACGACTCGTCACACACTCACATC	81	173	8	111.97	50	1	0
	GGGTCTTGTTTCTTCTCTGTGCAATACGACCAAACAAAGCTATATGACTTGAAGCCCGGAAACTCGTCACACACTCACATC	81	173	8	111.97	51	1	0
	GGGTCTTGTTTCTTCTCTGTGTCTCACTCAACGAATATACGATTCATTAAAGCGATGTGAACTCGTCACACACTCACATC	80	204	7	97.97	52	1	0
	GGGTCTTGTTTCTTCTCTGTGCGTAGACGTATCACTAAACATTTCCGTCCGATTCACTTCAACTCGTCACACACTCACATC	81	204	7	97.97	53	1	0
	GGGTCTTGTTTCTTCTCTGTGGACTATGCGGACTCACTGTCAACTGCTTAAGACGGCTCAAACTCGTCACACACTCACATC	81	204	7	97.97	54	1	0
	GGACTCACAGCTCAGGGCGCCTACAACCTAGCTCGTAGCATCTGCCATGCAAAGCGTCCCATGCGAGTGCAGGTG	75	204	7	97.97	55	1	0
	GGGTCTTGTTTCTTCTCTGTGATAATACGAATCCCTCTAATATGCACTCACCCTGATGTGAACTCGTCACACACTCACATC	81	204	7	97.97	56	1	0
	GGGTCTTGTTTCTTCTCTGTGTGTAATTTGTTTTACT	37	204	7	97.97	57	1	0
	GGCGACTATAACCTCAATATACGATAGCTAGCGATTTAATAAGGTCAATTGCGGTTCCATGTCATTGGA	69	204	7	97.97	58	1	0
	GGACTCACAGCTCAGGGTATTTGCTCCTTTATAACATCCCCCCTGCGCACCTTGCGCCCCATGCGAGTGCAGGTG	75	204	7	97.97	59	1	0
	GGCGACTATAACCTATAGCGAAACGTATAATCAAGTAATACGTCTTTGTATGGGTTCCATGTCATTGGA	69	204	7	97.97	60	1	0
	GGGTCTTGTTTCTTCTCTGTGTCGTCCACTATACTAAGCCATTGCGGCTCGCTTTTAACGAACTCGTCACACACTCACATC	81	204	7	97.97	61	1	0
	GGGTCTTGTTTCTTCTCTGTGTCCCACTAAATACGAACTATTAATACGACGACTAAGCCTGACTCGTCACACACTCACATC	81	204	7	97.97	62	1	0
	GGCGACTATAACCTGCAAAACAATATTAAGAGCTTGGTTTGCGTATTTGGGCGTTCCATGTCATTGGA	68	204	7	97.97	63	1	0
	GGCGACTATAACCTATTAAATAGCTAAAAAGAACCTATTTATTTGTGGTTGGCGTTCCATGTCATTGGA	69	204	7	97.97	64	1	0
	GGGTCTTGTTTCTTCTCTGTGATGCCACTCATTATTAACCCACGATTCGACTCGGTAGACTACTCGTCACACACTCACATC	81	204	7	97.97	65	1	0
	GGCGACTGTAACCTAACAAATTAATCCTTATCAAGAACAGGATACTCAGTTGGGTTCCATGTCATTGGA	69	204	7	97.97	66	1	0
	GGGTCTTGTTTCTTCTCTGTGATATACGATTGCCTATGGATACGGCCACTATAGCTCTCAAACTCGTCACACACTCACATC	81	204	7	97.97	67	1	0
	GGGTCTTGTTTCTTCTCTGTGCGATCTCTAAAATCTGACTCTCTATAAACGGACTCATTTAACTCGTCACACACTCACATC	81	204	7	97.97	68	1	0
	GGGTCTTGTTTCTTCTCTGTGCTTTCTTCGGCAATTAGACTCACACATACGGATTCACACGACTCGTCACACACTCACATC	81	204	7	97.97	69	1	0
	GGCGACTATAACCTTAACAGTAGCAGGTATAGAAACGTATGGAATTGTTTTGGCGTTCCATGTCATTGGA	70	204	7	97.97	70	1	0
	GGACTCACAGCTCAGGGTAGTGCTGACTACTGGCCCCGACCGGATCCCCCACTAGTCCCCATGCGAGTGCAGGTG	75	242	6	83.98	71	1	0
	GGGTCTTGTTTCTTCTCTGTGCAAAGATCCAATCCCATAATACGGCTTATCCACGCAATAAACTCGTCACACACTCACATC	81	242	6	83.98	72	1	0
	GGGTCTTGTTTCTTCTCTGTGTCCGTCAAGTATTGTCCAACCTTGTGAGCTTCTTTGCCTTACTCGTCACACACTCACATC	81	242	6	83.98	73	1	0
	GGGTCTTGTTTCTTCTCTGTGGGTTAATACGCACACTAATGACATCACTAAACATTACGCCACTCGTCACACACTCACATC	81	242	6	83.98	74	1	0
	GGGTCTTGTTTCTTCTCTGTGCGCACTATACTACTAACGACAAAACTGTGATTGCGAACTCACTCGTCACACACTCACATC	81	242	6	83.98	75	1	0
	GGGTCTTGTTTCTTCTCTGTGATACGACGACTATAACATACGAAATTCACGGACTCGTCACACACTCACATC	72	242	6	83.98	76	1	0
	GGGTCTTGTTTCTTCTCTGTGATCTCCTTACCGTTTAATACGACTCGCAGTTCGTTCTCTCACTCGTCACACACTCACATC	81	242	6	83.98	77	1	0
	GGGTCTTGTTTCTTCTCTGTGGGCTACTCACTGGACGCGATATATTAATACGGACGCCTAAACTCGTCACACACTCACATC	81	242	6	83.98	78	1	0
	GGGTCTTGTTTCTTTCTGTGGGATGCTCGACATAATACGATTAACTCCGTCATACGACTTACGACTCGTCACACACTCACATC	83	242	6	83.98	79	1	0
	GGGTCTTGTTTCTTCTCTGTGCAACATTCCTTATGCAATATCCTACCTGCTGAACTCTGGAAACTCGTCACACACTCACATC	82	242	6	83.98	80	1	0
	GGCGACTATAACCTGGTGAAGTATTATAAAGAGCATATTTCAGGGTGGGTGCGGTTCCATGTCATTGGA	69	242	6	83.98	81	1	0
	GGGTCTTGTTTCTTCTCTGTGCTTTTTTATAATGACTTCTATAAATACGACCACCTGGCACGACTCGTCACACACTCACATC	82	242	6	83.98	82	1	0
	GGGTCTTGTTTCTTCTCTGTGCTCTTCCTTCACATTAATGTCGACTCACACGTTTTCTAGACACTCGTCACACACTCACATC	82	242	6	83.98	83	1	0
	GGGTCTTGTTTCTTCTCTGTGGCATAACCTCAGACTGCCACAATAACTACCCTTAGTATGACTCGTCACACACTCACATC	80	242	6	83.98	84	1	0
	GGGTCTTGTTTCTTCTCTGTGATCAAGCTCACTATAAGTCACTGTCCTCGCTGTACTAACGACTCGTCACACACTCACATC	81	242	6	83.98	85	1	0
	GGGTCTTGTTTCTTCTCTGTGAACGACTCTGAATATGACTTGGCTATATCGCAGGTACTGAACTCGTCACACACTCACATC	81	242	6	83.98	86	1	0
	GGGTCTTGTTTCTTCTCTGTGTTGATACGACATGCATCTGGCGACTTACTTTATATGGCTCACTCGTCACACACTCACATC	81	242	6	83.98	87	1	0
	GGGTCTTGTTTCTTCTCTGTGTACGTGCTTTGCCTAAATTGTTACTCCCTATATACTACTCACTCGTCACACACTCACATC	81	242	6	83.98	88	1	0
	GGGTCTTGTTTCTTCTCTGTGTGATGAAACTATGGTAGTAATACGACTGGCGATAAGGGCAACTCGTCACACACTCACATC	81	242	6	83.98	89	1	0
	GGCGACTATAACCTAACTATCAAGAAATTAGGAAAACCGAAAAGGTACACGGCATTCCATGTCATTGGA	69	242	6	83.98	90	1	0
	GGGTCTTGTTTCTTCTCTGTGCTGCTGAGGACCCCCATACGATCGAACTATAATACGTGTGACTCGTCACACACTCACATC	81	242	6	83.98	91	1	0
	GGGTCTTGTTTCTTCTCTGTGGCTTACTATTCATTATACTTCCTTCCACTAATCCCTGGTCACTCGTCACACACTCACATC	81	242	6	83.98	92	1	0
	GGGTCTTGTTTCTTCTCTGTGGATGGCGACAAGAAAAAAGGAGGGATTAATATGACTCACAAAACTCGTCACACACTCACATC	83	242	6	83.98	93	1	0
	GGGTCTTGTTTCTTCTCTGTGTTAATTCTTTTCCTTTATCCCATGATGTATCCTCCACTCGTCACACACTCACATC	76	242	6	83.98	94	1	0
	GGGTCTTGTTTCTTCTCTGTGTGCATACGACTTACTCACTAATAGACCTGGTAATACGACACTCGTCACACACTCACATC	80	242	6	83.98	95	1	0
	GGGTCTTGTTTCTTCTCTGTGTGGAATCACTAACTAGTACGGCAACTAATGGACCAATGGTACTCGTCACACACTCACATC	81	242	6	83.98	96	1	0
	GGCGACTATAACCTACACAAAATTTGTTTACTATTTTACTGTCATTGTGGGCGGTTCCATGTCATTGGA	69	242	6	83.98	97	1	0
	GGGTCTTGTTTCTTCTCTGTGTTGATACGCTCACTGGCTGCAATTTGTACTCGCTGCTTATACTCGTCACACACTCACATC	81	242	6	83.98	98	1	0
	GGGTCTTGTTTCTTCTCTGTGACTCATAAAATTCGACTATTACTTATACCGACGCTAACGCACTCGTCACACACTCACATC	81	242	6	83.98	99	1	0
	GGGTCTTGTTTCTTCTCTGTGACGGCACCATAAACTTTCCTACGTTTGCATAATACGACGAACTCGTCACACACTCACATC	81	242	6	83.98	100	1	0
	GGGTCTTGTTTCTTCTCTGTGCAGCTCACTTAAATACGATACATTATAGTATAATATGCCCACTCGTCACACACTCACATC	81	242	6	83.98	101	1	0
	GGGTCTTGTTTCTTCTCTGTGTTGCGCGTGATACCGCTTACTATATCGAGCGTCCCATTCCACTCGTCACACACTCACATC	81	242	6	83.98	102	1	0
	GGGTCTTGTTTCTTCTCTGTGTACGACGCATAAAAATGCTCATTTATCCCCATACGACCTAGACTCGTCACACACTCACATC	82	242	6	83.98	103	1	0
	GGGTCTTGTTTCTTCTCTGTGAACGTTCACCATACCTAGATTAATACGATACTTAATACGAACTCGTCACACACTCACATC	81	242	6	83.98	104	1	0
	GGGTCTTGTTTCTTCTCTGTGGTTTCCAACACGATGAACTATATCAAACATACGCGGACTAACTCGTCACACACTCACATC	81	242	6	83.98	105	1	0
	GGGTCTTGTTCTTCTCTGTGTG	22	242	6	83.98	106	1	0
	GGGTCTTGTTTCTTCTCTGTGAATTAGTACTGGCTCAACTGCGATTCACTATACTGATGAACTCGTCACACACTCACATC	80	242	6	83.98	107	1	0
	GGGTCTTGTTTCTTCTCTGTGAGAATTAGCAACGAGGCTTATTCCCAATCCTCACAGCATGACTCGTCACACACTCACATC	81	242	6	83.98	108	1	0
	GGGTCTTGTTTCTTCTCTGTGTAAGAAACACGGACTGAGACTCACGCGACTAACACTATAGACTCGTCACACACTCACATC	81	242	6	83.98	109	1	0
	GGACTCACAGCTCAGGGGATAATGGATAATGCATATAGTTGCCAAGAAATTACGCGGCCCATGCGAGTGCAGGTG	75	242	6	83.98	110	1	0
	GGGTCTTGTTTCTTCTCTGTGATACTCACTATAGGTTCTCGATTCGACTCAGCTATATTATACTCGTCACACACTCACATC	81	242	6	83.98	111	1	0
	GGGTCTTGTTTCTTCTCTGTGACTCACTCCGCTGATCGGCAATTTCACTATATTCCTTCCAACTCGTCACACACTCACATC	81	242	6	83.98	112	1	0
	GGCGACTATAACCTAACCTGTACAGTTCTAAAAGTAGTGAATTGCGCGGTGTGGTTCCATGTCATTGGA	69	242	6	83.98	113	1	0
	GGGTCTTGTTTCTTCTCTGTGGATGACCAATTACGACTTTCACCCGGACGCACTATAAACGACTCGTCACACACTCACATC	81	242	6	83.98	114	1	0
	GGGTCTTGTTTCTTCTCTGTGCTCACTATTTAATACGAACTCGCACTAGCACGATTAAACCACTCGTCACACACTCACATC	81	242	6	83.98	115	1	0
	GGGTCTTGTTTCTTCTCTGTGGTTAATTACGACAAACTACGATGTGTTAATACGAACTTCTACTCGTCACACACTCACATC	81	317	5	69.98	116	1	0
	GGGTCTTGTTTCTTCTCTGTGAATGCGGCCACGATATGACTCTGAGCGCCGCTATGAACTTACTCGTCACACACTCACATC	81	317	5	69.98	117	1	0
	GGGTCTTGTTTCTTCTCTGTGTTCGACCACTAGAAATACCTCACGCGGCTAATAACACTAAACTCGTCACACACTCACATC	81	317	5	69.98	118	1	0
	GGGTCTTGTTTCTTCTCTGTGAATACCATCTCACAAATGAATACCACTTACTACAAATATGACTCGTCACACACTCACATC	81	317	5	69.98	119	1	0
	GGACTCACAGCTCAGGGGCCTAGCTAAGACGACAAGACCATAACTACAGCAGCCAGACCCATGCGAGTGCAGGTG	75	317	5	69.98	120	1	0
	GGGTCTTGTTTCTTCTCTGTGATACGAAGAGCTAATAGGATTCACTAACTCACTAAATATGACTCGTCACACACTCACATC	81	317	5	69.98	121	1	0

Table S2. Sequence data analyzed with FASTAptamer (2/5)

Name[*1]	Sequence (5' to 3')	Length[*2]	Rank[*3]	Reads[*4]	RPM[*5]	Cluster[*6]	Rank in Cluster[*7]	Edit Distance[*8]
	GGGTCTTGTTTCTTCTCTGTGCATTACGACCTCAGGCTCACGTATATGACTAACTAAACTTACTCGTCACACACTCACATC	81	317	5	69.98	122	1	0
	GGGTCTTGTTTCTTCTCTGTGTATAAATAATAGGCACTCAACGTATGGACACACTATGGCTACTCGTCACACACTCACATC	81	317	5	69.98	123	1	0
	GGGTCTTGTTTCTTCTCTGTGTTGGAAGAGACCATAACGCAACCATTCGACTCCTTTGAAAACTCGTCACACACTCACATC	81	317	5	69.98	124	1	0
	GGACTCACAGCTCAGGGCAAAGTGGCATTCCTCTCATTGACTTCGGTCCAGGCAGCGCCCATGCGAGTGCAGGTG	75	317	5	69.98	125	1	0
	AGGTCTTGTTTCTTCTCTGTGATACGACTTCTAACATGCAACCACCTAATATCGGCTCATAACTCGTCACACACTCACATC	81	317	5	69.98	126	1	0
	GGGTCTTGTTTCTTCTCTGTGATCCCTCTACTCACTATATCGACTATACATGCGACATACCACTCGTCACACACTCACATC	81	317	5	69.98	127	1	0
	GGGTCTTGTTTCTTCTCTGTGCTTCCTTTGCATGTCTACTGCCGAAATCTATCCTTCTCCGACTCGTCACACACTCACATC	81	317	5	69.98	128	1	0
	GGGTCTTGTTTCTTCTCTGTGACGCGCTCACTGAAACTCCGAACTCACAATGACGACTCACACTCGTCACACACTCACATC	81	317	5	69.98	129	1	0
	GGGTCTTGTTTCTTCTCTGTGATGCGACTACACTTTGTACGATTCTATGAAGGCTCACTGCACTCGTCACACACTCACATC	81	317	5	69.98	130	1	0
	GGGTCTTGTTTCTTCTCTGTGGAATACGACCACACGATTCGCTCACTGTAATTAATGCATCACTCGTCACACACTCACATC	81	317	5	69.98	131	1	0
	GGGTCTTGTTTCTTCTCTGTGCTATGTACGATTCACTAAGAGGCCAACGTCACACTATCACACTCGTCACACACTCACATC	81	317	5	69.98	132	1	0
	GGGTCTTGTTTCTTCTCTGTGACGCAGCACTCACTATAAGTAGACTGAAAAGGAAAAATTCACTCGTCACACACTCACATC	81	317	5	69.98	133	1	0
	GGACTCACAGCTCAGGGGTAATTCGTCTGAAAGATCTCCAACCAACATGGAGCCTCCCCCATGCGAGTGCAGGTG	75	317	5	69.98	134	1	0
	GGGTCTTGTTTCTTCTCTGTGTAAGACTTAATATGTTCCTATGTAACCCTCTCTGCGACTTACTCGTCACACACTCACATC	81	317	5	69.98	135	1	0
	GGGTCTTGTTTCTTCTCTGTGATAGATAACGACACAGCCTCGACCCAGAAGCTCTCATACGACTCGTCACACACTCACATC	81	317	5	69.98	136	1	0
	GGGTCTTGTTTCTTCTCTGTGATACGACACACATCTTATGACTCCTCGATGCAGACTCACAACTCGTCACACACTCACATC	81	317	5	69.98	137	1	0
	GGGTCTTGTTTCTTCTCTGTGTTATGTCCATGCGACTTCGTCGCTTTACTCCCGCGTTCCACTCGTCACACACTCACATC	80	317	5	69.98	138	1	0
	GGCGACTATAACCTCTCGTAATGCATTCATATAAAGAACTGGATGCGGGTGGGGTTCCATGTCATTGGA	69	317	5	69.98	139	1	0
	GGGTCTTGTTTCTTCTCTGTGTTGTGAATAATAAGGGTGGGTACGACTCTAATACGGCTTAACTCGTCACACACTCACATC	81	317	5	69.98	140	1	0
	GGGTCTTGTTTCTTCTCTGTGCAGGAAGTTAATGCGACTAATATAGTAGCGAAAGGATAGCACTCGTCACACACTCACATC	81	317	5	69.98	141	1	0
	GGGTCTTGTTTCTTCTCTGTGCTTACTATCACTTACTATATTAACATGTCACTGTCTCGAGACTCGTCACACACTCACATC	81	317	5	69.98	142	1	0
	GGGTCTTGTTTCTTCTCTGTGATACGAAAACTAGCGACGCACTAGCTAAACTGACTCACTACTCGTCACACACTCACATC	80	317	5	69.98	143	1	0
	GGGTCTTGTTTCTTCTCTGTGGCGATGATAGAAAGAATATGACTACTCCTGATTGATACGACTCGTCACACACTCACATC	80	317	5	69.98	144	1	0
	GGGTCTTGTTTCTTCTCTGTGTGATACGATTCGCTAACGACTCACTATTTGTACGTCTCACACTCGTCACACACTCACATC	81	317	5	69.98	145	1	0
	GGGTCTTGTTTCTTCTCTGTGAAATGGTCTTAATACGACTTACCAAGATGTGAATCGTGACACTCGTCACACACTCACATC	81	317	5	69.98	146	1	0
	GGGTCTTGCTTCTTCTCTGTGACAAAACTAATACGCTCACGACTAAATACGACTAGGCCTGACTCGTCACACACTCACATC	81	317	5	69.98	147	1	0
	GGGTCTTGTTTCTTCTCTGTGGTATGACTCACTTGATACGCGCTATAATATAGATCCGACGACTCGTCACACACTCACATC	81	317	5	69.98	148	1	0
	GGGTCTTGTTTCTTCTCTGTGATACGACAATGCAAGACTGATACTACAACGCTATAAAACACTCGTCACACACTCACATC	80	317	5	69.98	149	1	0
	GGGTCTTGTTTCTTCTCTGTGGAAGTACCAAAGGCGCAACACTATCTAGTACATCTTCTCACTCGTCACACACTCACATC	80	317	5	69.98	150	1	0
	GGGTCTTGTTTCTTCTCTGTGACCCAATACAATACGACTATAATACGATTGGAACTAGAAACTCGTCACACACTCACATC	80	317	5	69.98	151	1	0
	GGGTCTTGTTTCTTCTCTGTGATACGACTAAATACGCTCACCTAGTATATGCCAGTTTTCACTCGTCACACACTCACATC	80	317	5	69.98	152	1	0
	GGGTCTTGTTTCTTCTCTGTGATCGTACTCAGTATTAATACCACCACTAAATAATCCAATCACTCGTCACACACTCACATC	81	317	5	69.98	153	1	0
	GGGTCTTGTTTCTTCTCTGTGGTATACGACTAGCTATGATACTTCGCAACACAATTGATACTCGTCACACACTCACATC	79	317	5	69.98	154	1	0
	GGGTCTTGTTTCTTCTCTGTGCGAACTCACCAAAGAACTGCCGCTCACTACGACTCTTATAGGTACACTCGTCCACACTCACATC	85	317	5	69.98	155	1	0
	GGGTCTTGTTTCTTCTCTGTGAAAATGAGTTAATGATACCACACTACCTACTCGCTGATACACTCGTCACACACTCACATC	81	317	5	69.98	156	1	0
	GGGTCTTGTTTCTTCTCTGTGAAAACGCAACTCATTATAAACGACGAACAATAATACTTCACACTCGTCACACACTCACATC	82	317	5	69.98	157	1	0
	GGGTCTTGTTTCTTCTCTGTGTGTTTTGAATGCCGCAAGTAATACGATTCATAGGTCACAACTCGTCACACACTCACATC	80	317	5	69.98	158	1	0
	GGGTCTTGTTTCTTCTCTGTGTATCCCGACAAGACCTGGAGTATCGATCCCACTAAGTGTTACTCGTCACACACTCACATC	81	317	5	69.98	159	1	0
	GGGTCTTGTTTCTTCTCTGTGCTTGCCCCATAAGACTGACTATTCTTTCTCCGTAATCTACACTCGTCACACACTCACATC	81	317	5	69.98	160	1	0
	GGGTCTTGTTTCTTCTCTGTGCAATCTAAGAACCTAATTCGCTCACTAGCACCGCTCACTAACTCGTCACACACTCACATC	81	317	5	69.98	161	1	0
	GGGTCTTGTTTCTTCTCTGTGACATCACTATTCCACAAGGCTCATTATGGACCAAACAAAACTCGTCACACACTCACATC	80	317	5	69.98	162	1	0
	GGGTCTTGTTTCTTCTCTGTGATGCGGCTCACTAGAAAGGGCAATTCGTTACGCATAGATTACTCGTCACACACTCACATC	81	317	5	69.98	163	1	0
	GGACTCACAGCTCAGGGCATCTGTGACAAAACCAGCAGACCGAAAACAGTCCAAGTGCCCATGCGAGTGCAGGTG	75	317	5	69.98	164	1	0
	GGGTCTTGTTTCTTCTCTGTGCACTACCGACTCATTACTACGACTATAGACACATCTTCCCACTCGTCACACACTCACATC	81	317	5	69.98	165	1	0
	GGGTCTTGTTTCTTCTCTGTGTGGCCACGATACTGATCTCTACAACGACTTAATTTTACGACTCGTCACACACTCACATC	80	317	5	69.98	166	1	0
	GGGTCTTGTTTCTTCTCTGTGGCTCACTAACCGTTCACTTAATGCCGGCTTAAGTGGATATACTCGTCACACACTCACATC	81	317	5	69.98	167	1	0
	GGGTCTTGTTTCTTCTCTGTGCCCAAGATATAATACGCCTGCCTCACGTAAAATGATGGTTACTCGTCACACACTCACATC	81	317	5	69.98	168	1	0
	GGGTCTTGTTTCTTCTCTGTGAACTTACTATAAATACGGCTGTTAATATTGACTCAATATACTCGTCACACACTCACATC	80	317	5	69.98	169	1	0
	GGGTCTTGTTTCTTCTCTGTGCTTTCGACTCACATACGAACACCACTATATCTTGTAGGAAACTCGTCACACACTCACATC	81	317	5	69.98	170	1	0
	GGGTCTTGTTTCTTCTCTGTGGTATAAAGGGGTAAGTAGACTCACTTAATACGAAAAACGCACTCGTCACACACTCACATC	81	317	5	69.98	171	1	0
	GGGTCTTGTTTCTTCTCTGTGGCAGACGACTTCACTACAAAAACGACAAGCTATGCGAATCACTCGTCACACACTCACATC	81	317	5	69.98	172	1	0
	GGGTCTTGTTTCTTCTCTGTGACTTACCGGTAATATGTCTCACTACAATGGCAGATAGACGACTCGTCACACACTCACATC	81	317	5	69.98	173	1	0
	GGGTCTTGTTTCTTCTCTGTGACCTCGCATACTACTCACTATAGTCATGTAAGCTATTCCGACTCGTCACACACTCACATC	81	317	5	69.98	174	1	0
	GGGTCTTGTTTCTTCTCTGTGTGATTCACTATAAATACGTGCACTATTACTACGGCTACTAACTCGTCACACACTCACATC	81	317	5	69.98	175	1	0
	GGGTCTTGTTTCTTCTCTGTGCACGGGACCTACGACTTCTCGAATATCGGCTCCGTATATGACTCGTCACACACTCACATC	81	317	5	69.98	176	1	0
	GGGTCTTGTTTCTTCTCTGTGCTCCATGATTCACGAAATCTCTCTATACGTATAACTCACAACTCGTCACACACTCACATC	81	317	5	69.98	177	1	0
	GGGTCTTGTTTCTTCTCTGTGACTCCTAATACTATTCGACTCTAATGCACTTACTAAATACACTCGTCACACACTCACATC	81	317	5	69.98	178	1	0
	GGACTCACAGCTCAGGGCTCTACAGTTCTTGACGTATTTGGCGGGTCGACGCACGTCACCCATGCGAGTGCAGGTG	76	317	5	69.98	179	1	0
	GGGTCTTGTTTCTTCTCTGTGGTAAGACGATCACTATAATATATGAACACTAGACTGACACTCGTCACACACTCACATC	79	419	4	55.98	180	1	0
	GGGTCTTGTTTCTTCTCTGTGTATGGCTCCTACACCTTACCGACTCGCCTATAATACGACTACTCGTCACACACTCACATC	81	419	4	55.98	181	1	0
	GGGTCTTGTTTCTTCTCTGTGCCACGATACGACAAATACTATAGCGAGCCACCAAACAAAACTCGTCACACACTCACATC	80	419	4	55.98	182	1	0
	GGGTCTTGTTTCTTCTCTGTGTACGACATTCACCTATTCCTTTCGCTCCACGTCGACTAACTCGTCACACACTCACATC	79	419	4	55.98	183	1	0
	GGGTCTTGTTTCTTCTCTGTGTCACTCACTATAATAAGCGGGAACGACTATATCGACTTACTCGTCACACACTCACATC	79	419	4	55.98	184	1	0
	GGGTCTTGTTTCTTCTCTGTGATCTTACCTACCCTGAAATAGTACGACGAACTTACTTACGACTCGTCACACACTCACATC	81	419	4	55.98	185	1	0
	GGCCAGCTTACGAAACGGTATTGACAGTGTAGTGTATTTTTATCTTGCAGCCTTCAGAATGCTCGCCTAG	70	419	4	55.98	186	1	0
	GGGTCTTGTTTCTTCTCTGTGAAAATTTACCACTAACCCAAGAACGATTGCAGCGCAGACACTCGTCACACACTCACATC	80	419	4	55.98	187	1	0
	GGCGACTATAACCTTCAGTGATAATTGTGTGCATATGAAATTTGGTTCTCGTGGTTCCATGTCATTGGA	69	419	4	55.98	188	1	0
	GGGTCTTGTTTCTTCTCTGTGCGGCTTGTAACTATAATATGATCAACTAAGTCAAAATGCGACTCGTCACACACTCACATC	81	419	4	55.98	189	1	0
	GGGTCTTGTTTCTTCTCTGTGCTATTCGATTCACAACGTTATATTCCCAAATACGGTCACTACTCGTCACACACTCACATC	81	419	4	55.98	190	1	0
	GGGTCTTGTTTCTTCTCTGTGGCGGCCCATATGTGAACGATAAACGAATGACCCTCGGAAACTCGTCACACACTCACATC	80	419	4	55.98	191	1	0
	GGGTCTTGTTTCTTCTCTGTGTGAATACGCTCCTAAATAATACGACATTGTCTCGGCCTTACTCGTCACACACTCACATC	80	419	4	55.98	192	1	0
	GGGTCTTGTTTCTTCTCTGTGTTTACGATTCAATATGACTCCAACGTACCACTAATCCTCCACTCGTCACACACTCACATC	81	419	4	55.98	193	1	0
	GGGTCTTGTTTCTTCTCTGTGCTGACTCACTATACCGACATACGATTCATCTTTACTTCACACTCGTCACACACTCACATC	81	419	4	55.98	194	1	0
	GGGTCTTGTTTCTTCTCTGTGCCCAGACTAAGAAGCACCACAAGGCTTCCTCACTATATAAACTCGTCACACACTCACATC	81	419	4	55.98	195	1	0
	GGGTCTTGTTTCTTCTCTGTGGGCCATAATACGAACTTACTCGACACTACTATATAGATTTACTCGTCACACACTCACATC	81	419	4	55.98	196	1	0
	GGGTCTTGTTTCTTCTCTGTGTAGACCAATACGACTATTAATACGACTGTACGAATTCACGACTCGTCACACACTCACATC	81	419	4	55.98	197	1	0
	GGGTCTTGTTTCTTCTCTGTGCGATTCTCGTTACGGCTTACTTTTGTCCCTAAGTTTTGGCTACTCGTCACACACTCACATC	82	419	4	55.98	198	1	0
	GGGTCTTGTTTCTTCTCTGTGCTTACTACGATTCATGAAAGACTCACGGATGGGATTTGCTACTCGTCACACACTCACATC	81	419	4	55.98	199	1	0
	GGGTCTTGTTTCTTCTCTGTGATCTACTTAGACTTACTGTTGTACTCCTCACTAACGATTCACTCGTCACACACTCACATC	81	419	4	55.98	200	1	0
	GGGTCTTGTTTCTTCTCTGTGATGATACGATCTCACTATGTATAAATGACTCAAGGATGCGACTCGTCACACACTCACATC	81	419	4	55.98	201	1	0
	GGGTCTTGTTTCTTCTCTGTGTACTGCTGACTACTCACTAAAGACTTACTAGCTGACCGACTCGTCACACACTCACATC	79	419	4	55.98	202	1	0
	GGGTCTTGTTTCTTCTCTGTGCTTACAATCTCTGCCTCACTATGTTCATTCCTGTCCTTACACTCGTCACACACTCACATC	81	419	4	55.98	203	1	0
	GGGTCTTGTTTCTTCTCTGTGGCCGCGGGAATATGAACTCACAATACATACGCACTATTATACTCGTCACACACTCACATC	81	419	4	55.98	204	1	0
	GGGTCTTGTTTCTTCTCTGTGTACGTACTTACTGCACTCACTATATTGACTTTCCCACGTCACTCGTCACACACTCACATC	81	419	4	55.98	205	1	0
	GGGTCTTGTTTCTTCTCTGTGTTATGACTTGAACTATAGCGGCTTACTATAACAACGACACACTCGTCACACACTCACATC	81	419	4	55.98	206	1	0
	GGACTCACAGCTCAGGGCCATAGGATGGAAGATTCAAATCATCAACAGCTGGCCGGTCCCATGCGAGTGCAGGTG	75	419	4	55.98	207	1	0
	GGGTCTTGTTTCTTCTCTGTGAGCACGAACTTAGTACGAACACTACATAACGGCAACCTATACTCGTCACACACTCACATC	81	419	4	55.98	208	1	0
	GGGTCTTGTTTCTTCTCTGTGTACGAATCTACTATAAATCATACGACTCTGCTTTCTGTCACTCGTCACACACTCACATC	80	419	4	55.98	209	1	0
	GGGTCTTGTTTCTTCTCTGTGTTAATACGAACTCAATGATATAACTCTCCAGTGGCACCAAACTCGTCACACACTCACATC	81	419	4	55.98	210	1	0
	GGGTCTTGTTTCTTCTCTGTGAGCTGCCATCAAAAATCGTTCCCAAACCATTCCTTTTTTTGAAACTCGTCACACACTCACATC	85	419	4	55.98	211	1	0
	GGGTCTTGTTTCTTCTCTGTGAAGTTCTCAGAAATCGACTCACAGCACTCACAAAGTTCCTACTCGTCACACACTCACATC	81	419	4	55.98	212	1	0
	GGGTCTTGTTTCTTCTCTGTGGCTTGGTTCCCTTGGCTCTTGATACAACTCACGTATGATACTCGTCACACACTCACATC	80	419	4	55.98	213	1	0
	GGGTCTTGTTTCTTCTCTGTGCCTGATACGACTGACTGAAACAGCTCACTTAGGGTTTTGAACTCGTCACACACTCACATC	81	419	4	55.98	214	1	0
	GGCGACTATAACCTTAAGCAGCTTTTTATTCATATTTCATAACGAGTGCTGTGGGTTCCATGTCATTGGA	70	419	4	55.98	215	1	0
	GGGTCTTGTTTCTTCTCTGTGCGACTCAGTATTTACCTCACTAGATTTGACCTTAGCACATGACTCGTCACACACTCACATC	82	419	4	55.98	216	1	0
	GGGTCTTGTTTCTTCTCTGTGAAACCACTATAGAATATTACGACGACTATATTCAATTGGACTCGTCACACACTCACATC	80	419	4	55.98	217	1	0
	GGGTCTTGTTTCTTCTCTGTGCTTAATTCCTCTTTCACCCAGATTCACTACGTCTTCCTGTACTCGTCACACACTCACATC	81	419	4	55.98	218	1	0
	GGGTCTTGTTTCTTCTCTGTGACCCTGAGTACGACTTACAATGTTCCTCTTACGATTGCTGACTCGTCACACACTCACATC	81	419	4	55.98	219	1	0
	GGGTCTTGTTTCTTCTCTGTGCATGTGGAAATAGTACGCTCACTATTTAAGCCCGCGTGATACTCGTCACACACTCACATC	81	419	4	55.98	220	1	0
	GGGTCTTGTTTCTTCTCTGTGTGCGTCGACCTGAAATAATATCTCACTGTATACCCGTGGTACTCGTCACACACTCACATC	81	419	4	55.98	221	1	0
	GGGTCTTGTTTCTTCTCTGTGAAACTTCGATGCTATGCGACTTGCTACTGATGTTAATGCGACTCGTCACACACTCACATC	81	419	4	55.98	222	1	0
	GGGTCTTGTTTCTTCTCTGTGTGACCAATACGACGATACAACTCATATATACGGACAAGCCACTCGTCACACACTCACATC	81	419	4	55.98	223	1	0
	GGCGACTATAACCTTCAGGTACGGTTATAATTTTAGAGGGATTCATTTGGTGCGTTCCATGTCATTGGA	69	419	4	55.98	224	1	0
	GGGTCTTGTTTCTTCTCTGTGTGACTGATTCTCACTATATAAACCTCAGCGCATCAATACGACTCGTCACACACTCACATC	81	419	4	55.98	225	1	0
	GGCGACTATAACCTTATCAAGATCAGGTTAGTAATGTAGTGTGTTGGGTTGCCGTTCCATGTCATTGGA	69	419	4	55.98	226	1	0
	GGGTCTTGTTTCTTCTCTGTGTGTTCTAGCTGCTCGGTGCGCCCCTTCCTTTATCTGCCTTACTCGTCACACACTCACATC	81	419	4	55.98	227	1	0
	GGGTCTTGTTTCTTCTCTGTGCTCACTATATACGCATGTACCCTCGACCTTATCCATCAATACTCGTCACACACTCACATC	81	419	4	55.98	228	1	0
	GGGTCTTGTTTCTTCTCTGTGGCAATAATCGATGACTAACTATATAATGAATACGACGGACACTCGTCACACACTCACATC	81	419	4	55.98	229	1	0
	GGACCTTGCCACTCAGGGCACCACTCGATTCATGTTCCAATCACCACCCTTCGGCAGGCCCATGCGAGTGCAGGTG	76	419	4	55.98	230	1	0
	GGGTCTTGTTTCTTCTCTGTGTTATTCTCACCTCTCTATATTCTGGCGGTACACTACAACTCGTCACACACTCACATC	78	419	4	55.98	231	1	0
	GGGTCTTGTTTCTTCTCTGTGTCTAATACGGCTGGCAAGCACCAATAAAAAGATATGAACTCGTCACACACTCACATC	78	419	4	55.98	232	1	0
	GGGTCTTGTTTCTTCTCTGTGTAATGGGAAATGGGCTATCGACTTTCACTATGTAAATACGACTCGTCACACACTCACATC	81	419	4	55.98	233	1	0
	GGGTCTTGTTTCTTCTCTGTGGCACTTTACGGCTCCTCCATCTCGCTATTAGTGGCCTCACACTCGTCACACACTCACATC	81	419	4	55.98	234	1	0
	GGGTCTTGTTTCTTCTCTGTGCAATACGACGTTAGACAACATGGATATACAGCTTACAACTCGTCACACACTCACATC	78	419	4	55.98	235	1	0
	GGGTCTTGTTTCTTCTCTGTGGGGTAATACTCGACACACTAATGCTGAACTGATACGGCGTACTCGTCACACACTCACATC	81	419	4	55.98	236	1	0
	GGGTCTTGTTTCTTCTCTGTGCTTTAATGACTAACTATAAGACTTGACAGGATACGACTTAACTCGTCACACACTCACATC	81	419	4	55.98	237	1	0
	GGGTCTTGTTTCTTCTCTGTGATTCTTCTTAAACGGCTCGCAAATACGGCGCTCTTAAACTCGTCACACACTCACATC	78	419	4	55.98	238	1	0
	GGGTCTTGTTTCTTCTCTTGACACGAAACACTATATAACGATTCATAAACATAAAGCACCACTCGTCACACACTCACATC	80	419	4	55.98	239	1	0
	GGGTCTTGTTTCTTCTCTGTGCGACCACTATAGACAAAAACGACTTAGCTTAATCCTTGGCACTCGTCACACACTCACATC	81	419	4	55.98	240	1	0
	GGGTCTTGTTTCTTCTCTGTGACTGACGGATGTGGAACTCACTATCAAATACGCGACGCTACTCGTCACACACTCACATC	80	419	4	55.98	241	1	0
	GGCGACTATAACCTTGGACGATTCGGAATGTTTTTTGTTATGGGGGTTTTCGGCTCCATGTCATTGGA	69	419	4	55.98	242	1	0

Table S2. Sequence data analyzed with FASTAptamer (3/5)

Name[*1]	Sequence (5' to 3')	Length[*2]	Rank[*3]	Reads[*4]	RPM[*5]	Cluster[*6]	Rank in Cluster[*7]	Edit Distance[*8]
	GGGTCTTGTTTCTTCTCTGTGACTCACTTTATCTTTCCTCTAGATCCCTTGCGTTCCCGCTACTCGTCACACACTCACATC	81	419	4	55.98	243	1	0
	GGGTCTTGTTTCTTCTCTGTGGAAAGATCCCCTTGATTCACTAAAACGACTTAATACTATTCACTCGTCACACACTCACATC	82	419	4	55.98	244	1	0
	GGGTCTTGTTTCTTCTCTGTGATTAAGACTACGATTCGCACGGCTCCTATATCGGACGCTCACTCGTCACACACTCACATC	81	419	4	55.98	245	1	0
	GGGTCTTGTTTCTTCTCTGTGGCCGCGATACGATTGACTATACTAATTACGACTAGCTCCTACTCGTCACACACTCACATC	81	419	4	55.98	246	1	0
	GGGTCTTGTTTCTTCTCTGTGGTTAATACAACTCACGATAGCCCATGAATCGACTCATAATACTCGTCACACACTCACATC	81	419	4	55.98	247	1	0
	GGGTCTTGTTTCTTCTCTGTGACCGAAATATGAACTCGCTTTAATTCGACAAACTACGACGACTCGTCACACACTCACATC	81	419	4	55.98	248	1	0
	GGGTCTTGTTTCTTCTCTGTGCTGACCTCTGCATACTCACTATGACAATAAAGTCCAAACGACTCGTCACACACTCACATC	81	419	4	55.98	249	1	0
	GGGTCTTGTTTCTTCTCTGTGCTAATACGCTCCCTTAAATGCACCCGGTGCTTCCTTGTAAACTCGTCACACACTCACATC	81	419	4	55.98	250	1	0
	GGGTCTTGTTTCTTCTCTGTGTGCACTAACGCGACACACTATATACGGCTTGATATTGACTCGTCACACACTCACATC	78	419	4	55.98	251	1	0
	GGCGACTATAACCTAGTGAACTCACATAATAAAGTAATGTGTTTTTTTACGCGGTTCCATGTCATTGGA	69	419	4	55.98	252	1	0
	GGGTCTTGTTTCTTCTCTGTGATTACGACTCACGTGATCTCACTACACGACTCCTACATCGACTCGTCACACACTCACATC	81	419	4	55.98	253	1	0
	GGGTCTTGTTTCTTCTCTGTGACCCAATGATTCACTACATTAGACTCACTTACAGCCTTGGACTCGTCACACACTCACATC	81	419	4	55.98	254	1	0
	GGGTCTTGTTTCTTCTCTGTGTATACGACTCATGTTACACTCACGTATCACCAGTGCCGGCACTCGTCACACACTCACATC	81	419	4	55.98	255	1	0
	GGGTCTTGTTTCTTCTCTGTGCCATAATAGTCTCGGGGTATTAGACACACCATGTACCTTCACTCGTCACACACTCACATC	81	419	4	55.98	256	1	0
	GGGTCTTGTTTCTTCTCTGTGTTGCTTATGATACGACATACTAAATACGGCAAGCCAAACTACTCGTCACACACTCACATC	81	419	4	55.98	257	1	0
	GGGTCTTGTTTCTTCTCTGTGGAACCTATCCCGTGTTAGATGATTGCTTGGCATGATACGACTCGTCACACACTCACATC	80	419	4	55.98	258	1	0
	GGGTCTTGTTTCTTCTCTGTGTTACCGAAACATAAACATCCTGTTCCTAACGGATAATCGCACTCGTCACACACTCACATC	81	419	4	55.98	259	1	0
	GGCGACTATAACCTAAAAGGTGAACTCACGCGATGTCAAGTAATTGTGTGCGTTCCATGTCATTGGA	67	419	4	55.98	260	1	0
	GGGTCTTGTTTCTTCTCTGTGTCATTTCGTCGTCTCGTATTCGGCCCTTATAGTATGCTCAACTCGTCACACACTCACATC	81	419	4	55.98	261	1	0
	GGGTCTTGTTTCTTCTCTGTGTCGTAGTGACCCAACTACGCTAATCCGACATTACTATTGTACTCGTCACACACTCACATC	81	419	4	55.98	262	1	0
	GGGTCTTGTTTCTTCTCTGTGCGACTTACCTCAATGACCTCTGCTTACTATAGTTAATGCGACTCGTCACACACTCACATC	81	419	4	55.98	263	1	0
	GGGTCTTGTTTCTTCTCTGTGGGCTGTACCTAAAAAGACCTGGAATAACGTAAGCCAAGAATACTCGTCACACACTCACATC	82	419	4	55.98	264	1	0
	GGGTCTTGTTTCTTCTCTGTGAACGCTCATTATACATACGTCATTATATTTTGCCAACTACTCGTCACACACTCACATC	79	419	4	55.98	265	1	0
	GGGTCTTGTTTCTTCTCTGTGTTCATCTTCCGCCCACGGGACCCTAAGACATACTCTCTACTCGTCACACACTCACATC	79	419	4	55.98	266	1	0
	GGCGACTATAACCTGCGGGTAATACTATAAAGAACTATTACTTCATCCGTCGGATTCCATGTCATTGGA	69	419	4	55.98	267	1	0
	GGGTCTTGTTTCTTCTCTGTGACTCACCAGTAAATACGGCGAATTTCCCCTCCCAAATATGACTCGTCACACACTCACATC	81	419	4	55.98	268	1	0
	GGGTCTTGTTTCTTCTCTGTGCTTTGTACTCACTTGCTATCCTCGATAATATTCACTCACGACTCGTCACACACTCACATC	81	419	4	55.98	269	1	0
	GGGTCTTGTTTCTTCTCTGTGAAATAAAGTTCCTCTGGTAAATTATCAAACACAAATCCTAACTCGTCACACACTCACATC	81	419	4	55.98	270	1	0
	GGGTCTTGTTTCTTCTCTGTGCGACTAATGACGACACTCTATAATCTGACTTACCCTGTTTACTCGTCACACACTCACATC	81	419	4	55.98	271	1	0
	GGGTCTTGTTTCTTCTCTGTGTTTCAGGCTCACCCTTTCTCCTATTGTGCCTTGCTTACGAACTCGTCACACACTCACATC	81	419	4	55.98	272	1	0
	GGGTCTTGTTTCTTCTCTGTGTAAATCCCGAGACATACGACATACTAGCACATACGAACTCACTCGTCACACACTCACATC	81	419	4	55.98	273	1	0
	GGGTCTTGTTTCTTCTCTGTGATATTCGGTACTACTGGATACGATTCATAATACACCACTACTCGTCACACACTCACATC	80	419	4	55.98	274	1	0
	GGGTCTTGTTTCTTCTCTGTGACATCTAATACGACTTTACCATCGACTCGATATAGGAACCACTCGTCACACACTCACATC	81	419	4	55.98	275	1	0
	GGGTCTTGTTTCTTCTCTGTGACAGACTTAAAAGTACACCGCTATTAATACGATTCTCCCATACTCGTCACACACTCACATC	82	419	4	55.98	276	1	0
	GGGTCTTGTTTCTTCTCTGTGCGCGTTTAATACGACTCGCGTATATTACCCAAATTCGTACACTCGTCACACACTCACATC	81	419	4	55.98	277	1	0
	GGCGACTATAACCTTTGCATAACGATAAACTATCAAGAACATTTATCATGCGGGTTCCATGTCATTGGA	69	419	4	55.98	278	1	0
	GGGTCTTGTTTCTTCTCTGTGTCGCAATCATGTCTCTCTATACTACTCACATACTCACTGTACTCGTCACACACTCACATC	81	419	4	55.98	279	1	0
	GGGTCTTGTTTCTTCTCTGTGATTCACTATTCTCCGACGGCCTACTATACATTCACTCACTACTCGTCACACACTCACATC	81	419	4	55.98	280	1	0
	GGGTCTTGTTTCTTCTCTGTGGATGCTCACTATACAGGGCTCACTATAAATGGACTGTACACTCGTCACACACTCACATC	80	419	4	55.98	281	1	0
	GGACTCACAGCTCAGGGGGGGACGACGCAGCTACGACCAAGCCGCAGATTTAGGCCCCCATGCGAGTGCAGGTG	74	419	4	55.98	282	1	0
	GGGTCTTGTTTCTTCTCTGTGTACCCAAAATACGACTTGAGGAGCGACTCATCTAAAACTGGACTCGTCACACACTCACATC	82	419	4	55.98	283	1	0
	GGGTCTTGTTTCTTCTCTGTGGCAATATCAACTCTATTCCTACGGTCTTCCCGGTCTGTTACTCGTCACACACTCACATC	80	419	4	55.98	284	1	0
	GGGTCTTGTTTCTTCTCTGTGGCAAAGACTCTTAATATGACTGGCGACTCACATCCTACAACTCGTCACACACTCACATC	80	419	4	55.98	285	1	0
	GGGTCTTGTTTCTTCTCTGTGATACGATTTCCTTCCGACTATTATACGACTTCACCCGTTCACTCGTCACACACTCACATC	81	419	4	55.98	286	1	0
	GGGTCTTGTTTCTTCTCTGTGCGAATGACTCAAATACGTACGCCATCACTATCCTAACATGACTCGTCACACACTCACATC	81	419	4	55.98	287	1	0
	GGGTCTTGTTTCTTCTCTGTGCGACAAAACAAAATACAACTTACGTTAAGCGACTTCCACTAACTCGTCACACACTCACATC	82	419	4	55.98	288	1	0
	GGGTCTTGTTTCTTCTCTGTGAACGACTCCAATGTGACACACTATTAAGACTTACTATCCCACTCGTCACACACTCACATC	81	419	4	55.98	289	1	0
	GGGTCTTGTTTCTTCTCTGTGATATTCCCGATGTCAATACGACTACCTATCGTTCCCAGATACTCGTCACACACTCACATC	81	419	4	55.98	290	1	0
	GGGTCTTGTTTCTTCTCTGTGTACGATTCCAAAATATGACTCGCGTAATACGACTTGCGCCACTCGTCACACACTCACATC	81	419	4	55.98	291	1	0
	GGGTCTTGTTTCTTCTCTGTGTTCTTGAGCGCCCTTGATACGGCTTGAGACTTACTATAAAACTCGTCACACACTCACATC	81	419	4	55.98	292	1	0
	GGGTCTTGTTTCTTCTCTGTGCCACTGCTATTAGTTTCTGTTTCGGCCCTGATCCACTATCACTCGTCACACACTCACATC	81	419	4	55.98	293	1	0
	GGGTCTTGTTTCTTCTCTGTGTGTAATTTGTTTTACTGGGGGGTTAACCTAACAAGTGCAC	61	419	4	55.98	294	1	0
	GGGTCTTGTTTCTTCTCTGTGCTTGCCTGGTATGACTCACTATCAGTATACGAATAGCTAACTCGTCACACACTCACATC	80	419	4	55.98	295	1	0
	GGGTCTTGTTTCTTCTCTGTGCTATCTCACTGTATACGACTTCTAGGCTCACTATGATACGACTCGTCACACACTCACATC	81	419	4	55.98	296	1	0
	GGGTCTTGTTTCTTCTCTGTGGCTTCATACGTACTTGCTTATGTTCGGCTCGCTTACTCTAACTCGTCACACACTCACATC	81	419	4	55.98	297	1	0
	GGGTCTTGTTTCTTCTCTGTGTTGGTTTCACACGTACCGCAAGCTCCGCAAGTCTCTCAGCACTCGTCACACACTCACATC	81	419	4	55.98	298	1	0
	GGGTCTTGTTTCTTCTCTGTGGAGCTTGGCATACTCACTATAGACGAATTCCGCTAATACTACTCGTCACACACTCACATC	81	419	4	55.98	299	1	0
	GGGTCTTGTTTCTTCTCTGTGACTCATATCGCCTCATTATATGCGTAAAATCAAGCATTGCACTCGTCACACACTCACATC	81	419	4	55.98	300	1	0
	GGGTCTTGTTTCTTCTCTGTGACAGATTGTTAATACCACTCTGACTCATTATAAGATAGCGACTCGTCACACACTCACATC	81	419	4	55.98	301	1	0
	GGGTCTTGTTTCTTCTCTGTGATACTCACTTAGTCGGCTCACTTCGCACGGTTCACTGTATACTCGTCACACACTCACATC	81	419	4	55.98	302	1	0
	GGGTCTTGTTTCTTCTCTGTGGAAGATCGGGATACGACGATCTATAATTACAACGATTCACACTCGTCACACACTCACATC	81	419	4	55.98	303	1	0
	GGGTCTTGTTTCTTCTCTGTGTGTAATTTGTTTTACTGGGGGGGTTAACCTAACAAGTGCACACTCGTCACACACTCACATC	82	2	1578	22085.99	1	2	1
	GGCGACTATAACCTTAAGTTTATGAAATTTATCAAGATCAATTTCTGCTGTGGATTCCATGTCATTGGA	69	242	6	83.98	3	2	1
	GGGTCTCGTTCTT	13	419	4	55.98	12	2	3
	GGCGACTATAACCTTTATTTGCACTTTAAAAGTAGGGAGTGCATTGTTTTGGCGTTCCATGTCATTGGA	69	152	9	125.97	26	2	1
	GGGTCTTGTTTCTTCTCTGTGTGTAATTTGTTTTAC	36	242	6	83.98	57	2	1
	GGCGACTATAACCTGCAAAACAATATTAAGAGCTTGGTTTTGCGTATTTGGGCGTTCCATGTCATTGGA	69	317	5	69.98	63	2	1
	GGGTCTTGTTTCTTCTCTGTGTGTAATTTGTTTTACTGGGGGTTAACCTAACAAGTGCACACTCGTCACACACTCACATC	80	3	655	9167.51	1	3	1
	GGGTCTCGTTCTTCTCT	17	419	4	55.98	12	3	1
	GGCGACTATAACCTTTTATTTGCACTTTAAAAGTAGGGAGTGCATTGTTTTGGCGTTCCATGTCATTGGA	70	317	5	69.98	26	3	2
	GGGTCTTGTTTCTTCTCTGTGTGTAATTTGTTT	33	242	6	83.98	57	3	4
	GGGTCTTGTTTCTTCTCTGTGTGTAATTTGTTTTACTGGGGGGTTAACCTAACAAGTGCGCACTCGTCACACACTCACATC	81	4	514	7194.04	1	4	1
	GGTCTTGTTTCTT	13	419	4	55.98	12	4	5
	GGGTCTTGTTTCTTCTCTGTGTGTAATTTGTTTACTGG	38	419	4	55.98	57	4	3
	GGGTCTTGTTTCTTCTCTGTGTGTAATTTGTTTTACTGGGGGGTTAACCTAACAAGCGCACACTCGTCACACACTCACATC	81	5	364	5094.61	1	5	1
	GGGTCTTGTTT	11	419	4	55.98	12	5	6
	GGGTCTTGTTTCTTCTCTGTGTGTATTGTTTTAC	34	419	4	55.98	57	5	3
	GAGTCTTGTTTCTTCTCTGTGTGTAATTTGTTTTACTGGGGGGTTAACCTAACAAGTGCACACTCGTCACACACTCACATC	81	6	302	4226.85	1	6	1
	GGGTCTTGTTTCTTCTCTGTGTGTAATTTGTTTACTGGG	39	419	4	55.98	57	6	4
	GGGTCTTGTTTCTTCTCTGTGTGTAATTTGTTTTACTGGGGGGTTAACCTAACAAGTGCACTCGTCACACACTCACATC	79	7	288	4030.9	1	7	2
	GGGTCTTGTTTCTTCTCTGTGTGTAATTTTGTTTTACTGGGGGGTTAACCTAACAAGTGCACACTCGTCACACACTCACATC	82	8	283	3960.92	1	8	1
	GGGTCTTGTTTCTTCTCTGTGTGTAATTTGTTTTACTGGGGGGTTAACCTAACAAAGTGCACACTCGTCACACACTCACATC	82	9	272	3806.96	1	9	1
	GGGTCTTGTTTCTTCTCTGTGTGTAATTGTTTTACTGGGGGGTTAACCTAACAAGTGCACACTCGTCACACACTCACATC	80	10	258	3611.02	1	10	1
	GGGTCTTGTTTCTTCTCTGTGTGTAATTTGTTTTACTGGGGGGTTAACCTAACAAGTACACACTCGTCACACACTCACATC	81	11	216	3023.18	1	11	1
	GGGTCTTGTTTCTTCTCTGTGTGCAATTTGTTTTACTGGGGGGTTAACCTAACAAGTGCACACTCGTCACACACTCACATC	81	12	211	2953.2	1	12	1
	GGGTCTTGTTTCTTCTCTGTGTGTAATTTGTTTTACTGGGGGGCTAACCTAACAAGTGCACACTCGTCACACACTCACATC	81	13	188	2631.28	1	13	1
	GGGTCTTGTTTCTTCTCTGTGTAATTTGTTTTACTGGGGGGTTAACCTAACAAGTGCACACTCGTCACACACTCACATC	79	14	183	2561.3	1	14	2
	GGGTCTTGTTTCTTCTCTGTGTGTGATTTGTTTTACTGGGGGGTTAACCTAACAAGTGCACACTCGTCACACACTCACATC	81	15	174	2435.34	1	15	1
	GGGTCTTGTTTCTTCTCTGTGCGTAATTTGTTTTACTGGGGGGTTAACCTAACAAGTGCACACTCGTCACACACTCACATC	81	16	133	1861.49	1	16	1
	GGGTCTTGTTTCTTCTCTGTGTGTAATTTGTTTTTACTGGGGGGTTAACCTAACAAGTGCACACTCGTCACACACTCACATC	82	17	122	1707.54	1	17	1
	GGGTCTTGTTTCTTCTCTGTGTGTAATTTGTTTTACTGGGGGGTTAACCTAACGAGTGCACACTCGTCACACACTCACATC	81	18	119	1665.55	1	18	1
	GGTCTTGTTTCTTCTCTGTGTGTAATTTGTTTTACTGGGGGGTTAACCTAACAAGTGCACACTCGTCACACACTCACATC	80	18	119	1665.55	1	19	1
	GGGTCTTGTTTCTTCTCTGTGTGTAGTTTGTTTTACTGGGGGGTTAACCTAACAAGTGCACACTCGTCACACACTCACATC	81	20	111	1553.58	1	20	1
	GGGTCTTGTTTCTTCTCTGTGTGTAATTTGTTTTACTGGGGGGGTTAACCTAACAAGTGCGCACTCGTCACACACTCACATC	82	21	90	1259.66	1	21	2
	GGGTCTTGTTTCTTCTCTGTGTGTAACTTGTTTTACTGGGGGGTTAACCTAACAAGTGCACACTCGTCACACACTCACATC	81	22	77	1077.71	1	22	1
	GGGTCTTGTTTCTTCTCTGTGTGTAATTTGTTTTACTGGGGGGTTAACCTAACAAGTGTACACTCGTCACACACTCACATC	81	23	76	1063.71	1	23	1
	GGGTCTTGTTTCTTCTCTGTGTGTAATTTGTTTTACTGGGGGGTTAACCTAACAATTGCACACTCGTCACACACTCACATC	81	24	71	993.73	1	24	1
	GGGTCTTGTTTCTTCTCTGTGTGTAATTTGTTTTACTGGGGGGTTAACCTAACAAGTGCATACTCGTCACACACTCACATC	81	25	61	853.77	1	25	1
	GGGTCTTGTTTCTTCTCTGTGTGTAATTTGTTTTACTGGGGGGTTAACCTGACAAGTGCACACTCGTCACACACTCACATC	81	26	59	825.78	1	26	1
	GGGTCTTGTTTCTTCTCTGTGTGTAATTTGTTTACTGGGGGTTAACCTAACAAGTGCACACTCGTCACACACTCACATC	79	27	53	741.8	1	27	2
	GGGTCTTGTTTCTTCTCTGTGTGTAATTTGTTTTACCGGGGGGTTAACCTAACAAGTGCACACTCGTCACACACTCACATC	81	28	51	713.81	1	28	1
	GGGTCTTGTTTCTTCTCTGTGTGTAATTTGTTTTACTGGGGGGTTAACCTAACAAGTGCACACACTCGTCACACACTCACATC	83	29	49	685.81	1	29	2
	GGGTCTTGTTTCTTCTCTGTGTGTAATTTGTTTTTACTGGGGGGGTTAACCTAACAAGTGCACACTCGTCACACACTCACATC	83	30	48	671.82	1	30	2
	GGGTCTTGTTTCTTCTCTGTGTGTAATTTGCTTTACTGGGGGGTTAACCTAACAAGTGCACACTCGTCACACACTCACATC	81	31	46	643.82	1	31	1
	GGGTCTTGTTTCTTCTCTGTGTGTAATTCGTTTTACTGGGGGGTTAACCTAACAAGTGCACACTCGTCACACACTCACATC	81	31	46	643.82	1	32	1
	GGGTCTTGTTTCTTCTCTGTGTGTAATTTGTTTTACTGGGGGGTTAACCCAACAAGTGCACACTCGTCACACACTCACATC	81	31	46	643.82	1	33	1
	GGGTCTTGTTTCTTCTCTGTGTGTAATTTGTTTTACTGGGGGGTTAACCTAACAAATGCACACTCGTCACACACTCACATC	81	34	44	615.83	1	34	1
	GGGTCTTGTTTCTTCTCTGTGTGTAATTTGTTTTACTGGGGGGTCAACCTAACAAGTGCACACTCGTCACACACTCACATC	81	34	44	615.83	1	35	1
	GGGTCTTGTTTCTTCTCTGTGTGTAATTTGTTTTGCTGGGGGGTTAACCTAACAAGTGCACACTCGTCACACACTCACATC	81	34	44	615.83	1	36	1
	GGGTCTTGTTTCTTCTCTGTGTGTAATTTGTTTTACTGGGGGGTTAACCTAGCAAGTGCACACTCGTCACACACTCACATC	81	37	43	601.84	1	37	1
	GGGTCTTGTTTCTTCTCTGTGTGTATTTGTTTTACTGGGGGGTTAACCTAACAAGTGCACACTCGTCACACACTCACATC	80	37	43	601.84	1	38	1
	GGGTCTTGTTTCTTCTCTGTGTGTTAATTTGTTTTACTGGGGGGTTAACCTAACAAGTGCACACTCGTCACACACTCACATC	82	39	42	587.84	1	39	1
	GGGTCTTGTTTCTTCTCTGTGTGTAATTTGTTTTACTGGGGGGTTAGCCTAACAAGTGCACACTCGTCACACACTCACATC	81	39	42	587.84	1	40	1
	GGGTCTTGTTTCTTCTCTGTGTGTTATTTGTTTTACTGGGGGGTTAACCTAACAAGTGCACACTCGTCACACACTCACATC	81	39	42	587.84	1	41	1
	GGGTCTTGTTTCTTCTCTGTGTGTAATTTGTTTTACTGGGGGGTTAACCTAACAAGTTCACACTCGTCACACACTCACATC	81	42	41	573.84	1	42	1
	GGGTCTTGTTTCTTCTCTGTGTGTAATTTGTTTTACTGGGGGGTTAACCTAACAGGTGCACACTCGTCACACACTCACATC	81	42	41	573.84	1	43	1
	GGGTCTTGTTTCTTCTCTGTGTGTCATTTGTTTTACTGGGGGGTTAACCTAACAAGTGCACACTCGTCACACACTCACATC	81	44	40	559.85	1	44	1
	GGGTCTTGTTTCTTCTCTGTGTGTAATTTGTCTTACTGGGGGGTTAACCTAACAAGTGCACACTCGTCACACACTCACATC	81	44	40	559.85	1	45	1
	GGGTCTTGTTTCTTCTCTGTGTGTAATTTGTTTTACTGGGGAGTTAACCTAACAAGTGCACACTCGTCACACACTCACATC	81	46	39	545.85	1	46	1
	GGGTCTTGTTTCTTCTCTGTGTGTAATTTGTTTTACTGGGGGGTTAACCTAACAAGTGCACACTCGTCACACACTCAC	78	48	37	517.86	1	47	3
	GGGTCTTGTTTCTTCTCTGTGTGTAATTTGTTTTACTGGGAGGTTAACCTAACAAGTGCACACTCGTCACACACTCACATC	81	49	35	489.87	1	48	1

Table S2. Sequence data analyzed with FASTAptamer (4/5)

Name[*1]	Sequence (5' to 3')	Length[*2]	Rank[*3]	Reads[*4]	RPM[*5]	Cluster[*6]	Rank in Cluster[*7]	Edit Distance[*8]
	AGGTCTTGTTTCTTCTCTGTGTGTAATTTGTTTTACTGGGGGGTTAACCTAACAAGTGCACACTCGTCACACACTCACATC	81	50	34	475.87	1	49	1
	GGGTCTTGTTTCTTCTCTGTGTGTAATTTGTTTTACTGGGGGGTTAACCTAACAAGTGCACACTCGTCACACACTCACAT	80	50	34	475.87	1	50	1
	AGGGTCTTGTTTCTTCTCTGTGTGTAATTTGTTTTACTGGGGGGTTAACCTAACAAGTGCACACTCGTCACACACTCACATC	82	50	34	475.87	1	51	1
	GGGTCTTGTTTCTTCTCTGTGTGTAATTTGTTTTACTGGGGGGTTAACCTAACAAGTGCTCACTCGTCACACACTCACATC	81	53	33	461.87	1	52	1
	GGGTCTTGTTTCTTCTCTGTGTGTAATTTGTTCTACTGGGGGGTTAACCTAACAAGTGCACACTCGTCACACACTCACATC	81	54	32	447.88	1	53	1
	GGGTCTTGTTTCTTCTCTGTGTGTAATTTGTTTTACTGGGGGGGTTAACCTAACGAGTGCACACTCGTCACACACTCACATC	82	56	31	433.88	1	54	2
	GGGTCTTGTTTCTTCTCTGTGTGTAATTTGTTTTACTGGGGGGTTAACCTAACAAGAGCACACTCGTCACACACTCACATC	81	57	30	419.89	1	55	1
	GGGTCTTGTTTCTTCTCTGTGTGTAATTTGTTTTACTGGGGGGTTGACCTAACAAGTGCACACTCGTCACACACTCACATC	81	57	30	419.89	1	56	1
	GGGTCTTGTTTCTTCTCTGTGTGTAATTTGTTTTACTGGGGGTTGAACCTAACAAGTGCACACTCGTCACACACTCACATC	81	59	29	405.89	1	57	2
	GGGTCTTGTTTCTTCTCTGTGTGTAATTTGTTTTACTAGGGGGTTAACCTAACAAGTGCACACTCGTCACACACTCACATC	81	59	29	405.89	1	58	1
	GGGTCTTGTTTCTTCTCTGTGTGTAATTTGTTTTACTGGAGGGTTAACCTAACAAGTGCACACTCGTCACACACTCACATC	81	61	27	377.9	1	59	1
	GGGTCTTGTTTCTTCTCTGTGTGTGTAATTTGTTTTACTGGGGGGTTAACCTAACAAGTGCACACTCGTCACACACTCACATC	83	61	27	377.9	1	60	2
	GGGTCTTGTTTCTTCTCTGTGTGTAATTTGTTTTACTGGGGGGTTAACCTAACAAGTGGCACACTCGTCACACACTCACATC	82	63	25	349.9	1	61	1
	GGGTCTTGTTTCTTCTCTGTGTGTAATTTGTTTTACGGGGGGGTTAACCTAACAAGTGCACACTCGTCACACACTCACATC	81	63	25	349.9	1	62	1
	GGGTCTTGTTTCTTCTCTGTGTGTAATTTGTTTTACTGGGGGGTTAACCTAACCAGTGCACACTCGTCACACACTCACATC	81	63	25	349.9	1	63	1
	GGGTCTTGTTTCTTCTCTGTGTGTAATTTGTTTACTGGGGGGTTAACCTAACAAGTGCACACTCGTCACACACTCACATC	80	63	25	349.9	1	64	1
	GGGTCTTGTTTCTTCTCTGTGTGTAATCTGTTTTACTGGGGGGTTAACCTAACAAGTGCACACTCGTCACACACTCACATC	81	63	25	349.9	1	65	1
	GGGTCTTGTTTCTTCTCTGTGTGTAATATTGTTTTACTGGGGGGTTAACCTAACAAGTGCACACTCGTCACACACTCACATC	82	63	25	349.9	1	66	1
	GGGTCTTGTTTCTTCTCTGTGTGTAATTTGTTTTACTGGGGGGTTAACCTAACAAGTGGACACTCGTCACACACTCACATC	81	69	24	335.91	1	67	1
	GGGTCTTGTTTCCTCTCTGTGTGTAATTTGTTTTACTGGGGGGTTAACCTAACAAGTGCACACTCGTCACACACTCACATC	81	70	23	321.91	1	68	1
	GGGTCTTGTTTCTTCTCTGTGTGTAATTTGTTTTACTGGGGGGTTAACCTAACAGTGCACACTCGTCACACACTCACATC	80	70	23	321.91	1	69	1
	GGGTCTTGTTTCTTCTCTGTGTGTAATTTGTTTTACTGGGGGATTAACCTAACAAGTGCACACTCGTCACACACTCACATC	81	70	23	321.91	1	70	1
	GGGTCTTGTTTCTTCTCTGTGTGTAATTTGTTTTACTGGGGGGTTAACCTAATAAGTGCACACTCGTCACACACTCACATC	81	74	22	307.92	1	71	1
	GGGTCTTGTTTCTTCTCTGTGTGTAATTTGTTTTACTGAGGGGTTAACCTAACAAGTGCACACTCGTCACACACTCACATC	81	75	21	293.92	1	72	1
	GGGTCTTGTTTCTTCTCTGTGTGTAATTTGTTTCACTGGGGGGTTAACCTAACAAGTGCACACTCGTCACACACTCACATC	81	76	20	279.92	1	73	1
	GGGTCTTGTTTCTTCTCTGTGTGTAATTTGTTTTACTGGGGGGTTAATCTAACAAGTGCACACTCGTCACACACTCACATC	81	76	20	279.92	1	74	1
	GGGTCTTGTTTCTTCTCTGTGTGTAATTTGTTTTACTGGGGGGTTAACCTAACAAGTGCACACTCGTCACACCTCACATC	80	79	19	265.93	1	75	1
	GGGTCTTGTTTCTTCTCTGTGTATAATTTGTTTTACTGGGGGGTTAACCTAACAAGTGCACACTCGTCACACACTCACATC	81	82	18	251.93	1	76	1
	GGGTCTTGTTTCTTCTCTGTGTGTAATTTGTTTTACTGGGGGGTTAACCTAACAAGTGCACACTCGTCCACACTCACATC	80	82	18	251.93	1	77	1
	GGGTCTTGTTTCTTCTCTGTGTGTAATTTGTTTTACTGGGGGGTTAACCTAACAAGGGCACACTCGTCACACACTCACATC	81	82	18	251.93	1	78	1
	GGGTCTTGTTTCTTCTCTGTGTGTAAATTGTTTTACTGGGGGGTTAACCTAACAAGTGCACACTCGTCACACACTCACATC	81	86	17	237.94	1	79	1
	GGGTCTTGTTTCTTCTCTGTGTGTAAATTTGTTTTACTGGGGGGTTAACCTAACAAGTGCACACTCGTCACACACTCACATC	82	86	17	237.94	1	80	1
	GGGTCTTGTTTCTTCTCTGTGTGTAATTTGTTTTACTGGGGGGTTAACCTAACCAAGTGCACACTCGTCACACACTCACATC	82	86	17	237.94	1	81	1
	GGGTCTTGTTTCTTCTCTGTGTGTAATTTGTTTTACTGGGGGGTTAACCTAACAAGTGCACACTCGTCACACACCTCACATC	82	86	17	237.94	1	82	1
	GGGTCTTGTTTCTTCTCTGTGTGTAATTTGTTTTACTGGGGGGTTAACCTAACAAGTGCCCACTCGTCACACACTCACATC	81	90	16	223.94	1	83	1
	GGGTCTTGTTTCTTCTCTGTGTGTAATTTGTTTTACTGGGGGGTTAACCTAACTAGTGCACACTCGTCACACACTCACATC	81	90	16	223.94	1	84	1
	GGGTCTTGTTTCTTCTCTGTGTGTAATTTGGTTTACTGGGGGGTTAACCTAACAAGTGCACACTCGTCACACACTCACATC	81	90	16	223.94	1	85	1
	GGGTCTTGTTTCTTCTCTGTGTGTAATTTGTTTTACTGGGGGGTTAACCTAACAAGTTGCACACTCGTCACACACTCACATC	82	90	16	223.94	1	86	1
	GGGTCTTGTTTCTTCTCTGTGTGTAATTTGTTTTACTGGGGGGTTAACCTAACAAGTGCACACTCGTCACACACTCACTC	80	96	15	209.94	1	87	1
	GGGTCTTGTTTCTTCTCTGTGTGTAATTTGTTTTACTGGGGGGTTGAACCTAACAAGTGCACACTCGTCACACACTCACATC	82	96	15	209.94	1	88	1
	GGGTCTTGTTTCTTCTCTGTGTGTACTTTGTTTTACTGGGGGGTTAACCTAACAAGTGCACACTCGTCACACACTCACATC	81	96	15	209.94	1	89	1
	GGGTCTTGTTTCTTCTCTGTGTGTATTTTGTTTTACTGGGGGGTTAACCTAACAAGTGCACACTCGTCACACACTCACATC	81	96	15	209.94	1	90	1
	GGGTCTTGTTTCTTCTCTGCGTGTAATTTGTTTTACTGGGGGGTTAACCTAACAAGTGCACACTCGTCACACACTCACATC	81	96	15	209.94	1	91	1
	GGGTCTTGCTTCTTCTCTGTGTGTAATTTGTTTTACTGGGGGGTTAACCTAACAAGTGCACACTCGTCACACACTCACATC	81	102	14	195.95	1	92	1
	GGGTCTTGTTTCTTCTCTGTGTGTAATTTTTGTTTTACTGGGGGGTTAACCTAACAAGTGCACACTCGTCACACACTCACATC	83	102	14	195.95	1	93	2
	GGGTCTTGTTTCTTCTCTGTGTGTAATTTGTTTTACTGGGGGGTTAACCTAACAAGTGCACACTCGTACACACTCACATC	80	106	13	181.95	1	94	1
	GGGCCTTGTTTCTTCTCTGTGTGTAATTTGTTTTACTGGGGGGTTAACCTAACAAGTGCACACTCGTCACACACTCACATC	81	106	13	181.95	1	95	1
	GGGTCTTGTTTCTTCTCTGTGTGTAATTTTGTTTTACTGGGGGGGTTAACCTAACAAGTGCACACTCGTCACACACTCACATC	83	106	13	181.95	1	96	2
	GGGTCTTGTTTCTTCTCTGTGTGTAATTTGTTTTACTGGGGGGTTAACCTAACAAGTGCAAACTCGTCACACACTCACATC	81	106	13	181.95	1	97	1
	GGGTCTTGTTCCTTCTCTGTGTGTAATTTGTTTTACTGGGGGGTTAACCTAACAAGTGCACACTCGTCACACACTCACATC	81	106	13	181.95	1	98	1
	GGGTCTTGTTTCTTCTCTGTGTGTAATTTGTTTTACTGGGGGGGTTAACCTAACAAGTGCACTCGTCACACACTCACATC	80	106	13	181.95	1	99	3
	GGGTCTTGTTTCTTCTCTGTGTGTGATTTGTTTTACTGGGGGGTTAACCTAACAAGCGCACACTCGTCACACACTCACATC	81	106	13	181.95	1	100	2
	GGGTCCTGTTTCTTCTCTGTGTGTAATTTGTTTTACTGGGGGGTTAACCTAACAAGTGCACACTCGTCACACACTCACATC	81	114	12	167.95	1	101	1
	GGGTCTTGTTTCTTCTCTGTGTGTAATTTGTTTTACTGGGGGGTTAACCTAACAAGGTGCACACTCGTCACACACTCACATC	82	114	12	167.95	1	102	1
	GGGTCTTGTTTCTTCTCTGTGTGTAATTTGTTTTACTGGGGGGGCTAACCTAACAAGTGCACACTCGTCACACACTCACATC	82	114	12	167.95	1	103	2
	GGGTCTTGTTTCTTCTCTGTGTGTAATTTGTTTTACTGGGGGGGTTAACCTAACAAGCGCACACTCGTCACACACTCACATC	82	114	12	167.95	1	104	2
	GGGTCTTGTTCTTCTCTGTGTGTAATTTGTTTTACTGGGGGGTTAACCTAACAAGTGCACACTCGTCACACACTCACATC	80	114	12	167.95	1	105	1
	GGGTCTTGTTTCTTCTCTGTGTGTAATTTGTTTTACTGGGGGGTTAACCTAACAAGTGCACACTCGTCACCACTCACATC	80	123	11	153.96	1	106	1
	GGGTCTTGGTTTCTTCTCTGTGTGTAATTTGTTTTACTGGGGGGTTAACCTAACAAGTGCACACTCGTCACACACTCACATC	82	123	11	153.96	1	107	1
	GGGTCTTGTCTCTTCTCTGTGTGTAATTTGTTTTACTGGGGGGTTAACCTAACAAGTGCACACTCGTCACACACTCACATC	81	123	11	153.96	1	108	1
	GGGTCTTGTTTCTTCTCTGTGTGTAATTTGTTTTACTGGGGGGTTAACCTAACAAGTGTCACACTCGTCACACACTCACATC	82	123	11	153.96	1	109	1
	GGGTCTTGTTTCTTCTCTGTGTGTAATTTGTTTTACTGGGGGGTTAACCTAAGTGCACACTCGTCACACACTCACATC	78	123	11	153.96	1	110	3
	GGGTCTTGTTTCTTCTCTGTGTGTAATTGTTTTACTGGGGGGGTTAACCTAACAAGTGCACACTCGTCACACACTCACATC	81	134	10	139.96	1	111	2
	GGGTCTTGTTTCTTCTCTGTGTAATTTGTTTTACTGGGGGGGTTAACCTAACAAGTGCACACTCGTCACACACTCACATC	80	134	10	139.96	1	112	3
	GGGTCTTGTTTCTTCTCTGTGTGTAATTTGTTTTATTGGGGGGTTAACCTAACAAGTGCACACTCGTCACACACTCACATC	81	134	10	139.96	1	113	1
	GGGTCTTGTTTCTTCTCTGTGTGTAATTTGTTTTACTGGGGGGTTAACCTAACAAGTGCACACTCGTCACACATCACATC	80	134	10	139.96	1	114	1
	GAGTCTTGTTTCTTCTCTGTGTGTAATTTGTTTTACTGGGGGGGTTAACCTAACAAGTGCACACTCGTCACACACTCACATC	82	134	10	139.96	1	115	2
	GGGTCTTGTTTCTTCTCTGTGAGTAATTTGTTTTACTGGGGGGTTAACCTAACAAGTGCACACTCGTCACACACTCACATC	81	134	10	139.96	1	116	1
	GGGTCTTGTTTCTTCTCTGTGTGTAATTTGTTTTACTGGGGGGTTAACTTAACAAGTGCACACTCGTCACACACTCACATC	81	134	10	139.96	1	117	1
	GGGTCTTGTTTTCTTCTCTGTGTGTAATTTGTTTTACTGGGGGGTTAACCTAACAAGTGCACACTCGTCACACACTCACATC	82	134	10	139.96	1	118	1
	GGGTCTTGTTTCTTCTCTGTGTGTGATTTGTTTTACTGGGGGGTTAACCTAACAAGTGCGCACTCGTCACACACTCACATC	81	134	10	139.96	1	119	2
	GGGTCTTGTTTCTTCCCTGTGTGTAATTTGTTTTACTGGGGGGTTAACCTAACAAGTGCACACTCGTCACACACTCACATC	81	134	10	139.96	1	120	1
	GGGTCTTGTTTCTTCTCTGTGTGTAATTTGTTTTACTGGGGGGTTAACCTAACAAGTACACTCGTCACACACTCACATC	79	152	9	125.97	1	121	2
	GGGTCTTGTTTCTTCTCTGTGTGTAATTTGTTTTACTGGGGGGTTAACCTAACAAGTGCACACTCGTCAACACTCACATC	80	152	9	125.97	1	122	1
	GGGTCTTGTTTCTTCTCTGTGTGTAATTTGTTTTACTGGGGGGATAACCTAACAAGTGCACACTCGTCACACACTCACATC	81	152	9	125.97	1	123	1
	GAGTCTTGTTTCTTCTCTGTGTGTAATTTGTTTTACTGGGGGGTTGACCTAACAAGTGCACACTCGTCACACACTCACATC	81	152	9	125.97	1	124	2
	GGGTCTTGTTTCTTCTCTGTGTGTAATTTATTTTACTGGGGGGTTAACCTAACAAGTGCACACTCGTCACACACTCACATC	81	152	9	125.97	1	125	1
	GGGTCTTGTTTCTTCTCTGTGTGTAATTTGTTTTACTGGGGGGTTAACCTAACATGTGCACACTCGTCACACACTCACATC	81	152	9	125.97	1	126	1
	GGGTCTTGTTTCTTCTCTGTGTGTAATTTGTTTTACTGGGGGGTTAACCTAACAAGTGCACACTCTCACACACTCACATC	80	152	9	125.97	1	127	1
	GGGTCTTGTTTCTTCTCTGTGTGTAATTTGTTTTACTGGGGGGTTAACCTAACAAGTGCAGACTCGTCACACACTCACATC	81	152	9	125.97	1	128	1
	GGGTCTTGTTTCTTCTCTGTGTGTAATTTGTTTTACTGGGGGTTAACCTAACAAGCGCACACTCGTCACACACTCACATC	80	152	9	125.97	1	129	2
	GGGTCTTGTTTCTTCTCTGTGTGTAATTTGTTTTACTGGGGGTTGAACCTAACAAGTGACACACTCGTCACACACTCACATC	82	152	9	125.97	1	130	3
	GGGTCTTGTTTCTCCTCTGTGTGTAATTTGTTTTACTGGGGGGTTAACCTAACAAGTGCACACTCGTCACACACTCACATC	81	152	9	125.97	1	131	1
	GGGTCTTTTTTCTTCTCTGTGTGTAATTTGTTTTACTGGGGGGTTAACCTAACAAGTGCACACTCGTCACACACTCACATC	81	152	9	125.97	1	132	1
	GGGTCTTGTTTCTCTCTGTGTGTAATTTGTTTTACTGGGGGGTTAACCTAACAAGTGCACACTCGTCACACACTCACATC	80	152	9	125.97	1	133	1
	GGGCTTGTTTCTTCTCTGTGTGTAATTTGTTTTACTGGGGGGTTAACCTAACAAGTGCACACTCGTCACACACTCACATC	80	173	8	111.97	1	134	1
	GGGTCTTGTTTCTTCTCTGTGTGTAATTTGTTTTACTGGGGGGGTTAACCTAACAAAGTGCACACTCGTCACACACTCACATC	83	173	8	111.97	1	135	2
	GGGTCTTGTTTCTTCTCTGTGTGTAATTTGTTTTACAGGGGGGTTAACCTAACAAGTGCACACTCGTCACACACTCACATC	81	173	8	111.97	1	136	1
	GGGTCTTGTTTCTTCTCTGTATGTAATTTGTTTTACTGGGGGGTTAACCTAACAAGTGCACACTCGTCACACACTCACATC	81	173	8	111.97	1	137	1
	GGGTCTTGTTTCTTCTCTGTGTGTAATTTGTTTTACTGGGGGTTTAACCTAACAAGTGCACACTCGTCACACACTCACATC	81	173	8	111.97	1	138	1
	GGGTCTTGTTTCTTCTCTGTGTGTAATTTGTTTTACTGGGGGGTTAACCTAACAATGCACACTCGTCACACACTCACATC	80	173	8	111.97	1	139	1
	GGGTCTTGTTTCTTCTCTGTGTGTAATTTGTTTTACTGGGGGAGTTAACCTAACAAGTGCACACTCGTCACACACTCACATC	82	173	8	111.97	1	140	1
	GGGTCTGTTTCTTCTCTGTGTGTAATTTGTTTTACTGGGGGGTTAACCTAACAAGTGCACACTCGTCACACACTCACATC	80	173	8	111.97	1	141	1
	GGGTCTTGTTTCCTTCTCTGTGTGTAATTTGTTTTACTGGGGGGTTAACCTAACAAGTGCACACTCGTCACACACTCACATC	82	173	8	111.97	1	142	1
	GGGTCTTGTTTCTTCTCTGTGTGTAATTTGTTTTACTGGGGGGTTAACCTAACAAGTGCACACTCGTCACGCACTCACATC	81	173	8	111.97	1	143	1
	GGGTCTTGTTTCTTCTCTGTGTGTAATTAGTTTTACTGGGGGGTTAACCTAACAAGTGCACACTCGTCACACACTCACATC	81	173	8	111.97	1	144	1
	GGGTCTTGTTTCTTCTCTGTGTGTAATTTGTTTTACTGGGGGGTTAACCTAACAAGTGCACATCTCGTCACACACTCACATC	82	173	8	111.97	1	145	1
	GGGTCTTGTTTCTTCTCTGTGTGTAATTTGTTTTACTGGGGGTTAACCTAACAAAGTGCACACTCGTCACACACTCACATC	81	173	8	111.97	1	146	2
	GGGTCTTGTTTTCTTCTCTGTGTGTAATTTGTTTTTACTGGGGGTTAACCTAACAAGTGCACACTCGTCACACACTCACATC	82	173	8	111.97	1	147	3
	GGGTCTTGTTTCTTCTCTGTGTGTAATTTTGTTTTACTGGGGGGTTAACCTAACAAAGTGCACACTCGTCACACACTCACATC	83	173	8	111.97	1	148	2
	GGGTCTTGTTTCTTCTCTGTGGGTAATTTGTTTTACTGGGGGGTTAACCTAACAAGTGCACACTCGTCACACACTCACATC	81	173	8	111.97	1	149	1
	GGGTCTTGTTTCTTCTCTGTGTGTAATTTGTTTTACTGGGGGGTTAACCTAACAAGTGCACACTCGTCACAACTCACATC	80	173	8	111.97	1	150	1
	GGGTCTTGTTTCTTCTCTGTGTGTAATTTGTTTTTACTGGGGGGGGTTAACCTAACAAGTGCACACTCGTCACACACTCACATC	84	173	8	111.97	1	151	3
	GGGTCTTGTTTCTTCTCTGTGTGGAATTTGTTTTACTGGGGGGTTAACCTAACAAGTGCACACTCGTCACACACTCACATC	81	173	8	111.97	1	152	1
	GGGTCTTGTTTCTTCTCTGTGTGTAATTTGTTTTACTGGGGGTTAACCTAACAAGTGCGCACTCGTCACACACTCACATC	80	204	7	97.97	1	153	2
	GGGTCTTGTTTCTTCTCTGTGTGTAATTTGTTTTACTGGGGGGTTAACCTTAACAAGTGGCACACTCGTCACACACTCACATC	83	204	7	97.97	1	154	2
	GGGTCTTGTTTCTTCTCTGTGTGTGATTTGTTTTACTGGGGGGGTTAACCTAACAAGTGCACACTCGTCACACACTCACATC	82	204	7	97.97	1	155	2
	GGGTCTTGTTTCTTCTCTGTGTGTAATTTGTTTTACTGGGGGGTTAACCTAACAAGCTGCACACTCGTCACACACTCACATC	82	204	7	97.97	1	156	1
	GGGTCTTGTTTCTTCTCTGTGTGTAATTTGTTTTACGGGGGGGGTTAACCTAACAAGTGCACACTCGTCACACACTCACATC	82	204	7	97.97	1	157	2
	GGGTCTTGTTTCTTCTCTGTGTGTAGTTTGTTTTACTGGGGGGTTAACCTAACAAGCGCACACTCGTCACACACTCACATC	81	204	7	97.97	1	158	2
	GGGTCTTGTTTCTTCTCTGTGTGTAATTTGTTTTACGTGGGGGTTAACCTAACAAGTGCACACTCGTCACACACTCACATC	81	204	7	97.97	1	159	2
	GGGTCTTGTTTCTTCTCTGTGTGTAATTTGTTTTACTGGGGGGTTAACCTACAAGTGCACACTCGTCACACACTCACATC	80	204	7	97.97	1	160	1
	GGGTCTTGTTTCTTCTCTGTGTGTAATTTGTTTTACTGGGGGGTTAACCTAACAAGTGCACGCTCGTCACACACTCACATC	81	204	7	97.97	1	161	1
	GGGTCTTGTTTCTTCTCTGTGCGTAATTTGTTTTACTGGGGGGTTAACCTAACAAGTGCGCACTCGTCACACACTCACATC	81	204	7	97.97	1	162	2
	GGGTCTTGTTTCTTCTCTGTGTGTAATTTGTTTTACTGGGGGGTTAACCTAACAAGCACACACTCGTCACACACTCACATC	81	204	7	97.97	1	163	2
	GGGTCTTGTTTCTTCTCTGTGTGTAATTTGTTTTACTGGGGGGGTTAACCTGACAAGTGCACACTCGTCACACACTCACATC	82	204	7	97.97	1	164	2
	GGGTCTTGTTTCTTCTCTGTGTGTAATTTGTTTTACTGGGGGGTTAACCTAACAAGTGCACACTCGTCACACACTCAATC	80	204	7	97.97	1	165	1
	GGGTCTTGTTTCTTCTCTGTGTGTAATTGTTTTACTGGGGGTTAACCTAACAAGTGCACACTCGTCACACACTCACATC	79	204	7	97.97	1	166	2
	GGGTCTTGTTTCTTCTCCGTGTGTAATTTGTTTTACTGGGGGGTTAACCTAACAAGTGCACACTCGTCACACACTCACATC	81	204	7	97.97	1	167	1
	GGGTTCTTGTTTCTTCTCTGTGTGTAATTTGTTTTACTGGGGGGTTAACCTAACAAGTGCACACTCGTCACACACTCACATC	82	204	7	97.97	1	168	1
	GGGTCTTGTTTCTTCTCTGTGTGTAGTTTGTTTTACTGGGGGGGTTAACCTAACAAGTGCACACTCGTCACACACTCACATC	82	204	7	97.97	1	169	2

Table S2. Sequence data analyzed with FASTAptamer (5/5)

Name[*1]	Sequence (5' to 3')	Length[*2]	Rank[*3]	Reads[*4]	RPM[*5]	Cluster[*6]	Rank in Cluster[*7]	Edit Distance[*8]
	GGGTCTTGTTTCTTCTCTGTGTGTAATTTGTTTTACTGGGGGGTTAACCTAACAAGTGCACACTCGTCACACACTACATC	80	204	7	97.97	1	170	1
	GGGTCTTGTTTCTTCTCTGTGTGTAATTTGTTTTACTGGGGGGTTAACCTAACGAAGTGCACACTCGTCACACACTCACATC	82	204	7	97.97	1	171	1
	GGGTCTTGTTTCTTCTCTGTGTGTAATTTGTTTTACTGGGGGGTTAACCTAACAAGTGCACACTCGTCACACACTCACAC	80	242	6	83.98	1	172	1
	GGGTCTTGTTTCTTCTCTGTGTGTAATTTGTTTTACTGGGGGGTTAACCTAACAAGTGCACACTCGTCGCACACTCACATC	81	242	6	83.98	1	173	1
	GGGTCTTGTTTCTTCTCTGTGTGTAATTTGTTTTACTGGGGGGTTAACCTAACAAAGTGCGCACTCGTCACACACTCACATC	82	242	6	83.98	1	174	2
	GGGTCTTGTTTCTTCTCTGTGTGACATTTGTTTTACTGGGGGGTTAACCTAACAAGTGCACACTCGTCACACACTCACATC	81	242	6	83.98	1	175	2
	GGGTCTTGTTTCTTCTCTGTGTGTAATTTGTTTTACTGGGGGGTTAACCTAACAATTGCGCACTCGTCACACACTCACATC	81	242	6	83.98	1	176	2
	GGGTCTTGTTTCTTCTCTGTGTGTAATTTGTTTTACTGGGGGGGTTAACCTAAACAAGTGCACACTCGTCACACACTCACATC	83	242	6	83.98	1	177	2
	GGGTCTTGTTTCTTCTCTGTGTGTAATTTGTTTTACTGGGGGGTTAACCTAACAAGTACGCACTCGTCACACACTCACATC	81	242	6	83.98	1	178	2
	GGGTCTTGTTTCTTCTCTGTGTGTAATTTGTTTTACTGGGGGGTAACCTAACAAGTGCACACTCGTCACACACTCACATC	80	242	6	83.98	1	179	1
	GGGTCTTGTTTCTTCTCTGTGTGTAATTTGTTTTACTGGGGGGTTTAACCTAACAAGTGCACACTCGTCACACACTCACATC	82	242	6	83.98	1	180	1
	GGGTCTTGTTTCTTCTCTGTGTGTAATTTGTTTTACTGGGGGGTTAACCTAACAAGTACACACTCGTCACACACTCACA	79	242	6	83.98	1	181	3
	GGTCTTGTTTCTTCTCTGTGTGTAATTTGTTTTACTGGGGGGGTTAACCTAACAAGTGCACACTCGTCACACACTCACATC	81	242	6	83.98	1	182	2
	GGGTCTTGTTTCTTCTCTGTGTGTAATTTGTTTTACTGGGGGTTAACCTAACAAGTGCACTCGTCACACACTCACATC	78	242	6	83.98	1	183	3
	GAGTCTTGTTTCTTCTCTGTGTGTAATTTGTTTTACTGGGGGGTTAACCTAACAAGTGCGCACTCGTCACACACTCACATC	81	242	6	83.98	1	184	2
	GGGTCTTGTTTCTTCTCTGTGTGTAATTTGTTTTACTGGGGGGTTAACCTAACAAATGCGCACTCGTCACACACTCACATC	81	242	6	83.98	1	185	2
	GGGTCTTGTTTCTTCTCTGTGTGTAATCTGTTTTACTGGGGGGGTTAACCTAACAAGTGCACACTCGTCACACACTCACATC	82	242	6	83.98	1	186	2
	GGGTCTTGTTTCTTCTCTGTGTGTAATTTGTTTTACTGGGGGGTTAACTAACAAGTGCACACTCGTCACACACTCACATC	80	242	6	83.98	1	187	1
	GGGTCTTGTTTCTTCTCTGTGTGCAATTTGTTTTACTGGGGGTTAACCTAACAAGTGCACACTCGTCACACACTCACATC	80	242	6	83.98	1	188	2
	GGGTCTTGTTTCTTCTCTGTGGTGTAATTTGTTTTACTGGGGGGTTAACCTAACAAGTGCACACTCGTCACACACTCACATC	82	242	6	83.98	1	189	1
	GGGTCTTGTTTCTTCTCTGTGTGTAATTTGTTTTACTGGGGGGTTAACCTAAAAGTGCACACTCGTCACACACTCACATC	80	242	6	83.98	1	190	1
	GGGTCTTGTTTCTTCTCTGTGTGTAATTTTGTTTTACTGGGGGTTAACCTAACAAGTGCACACTCGTCACACACTCACATC	81	242	6	83.98	1	191	2
	GGGTCTTGTTTCTTCTCTGTGTGTAATTTGTCTTACTGGGGGGGTTAACCTAACAAGTGCACACTCGTCACACACTCACATC	82	242	6	83.98	1	192	2
	GGGTCTTGTTTCTTCTCTGTGTGAAATTTGTTTTACTGGGGGGTTAACCTAACAAGTGCACACTCGTCACACACTCACATC	81	242	6	83.98	1	193	1
	GGGTCTTGTTTCTTCTCTGTGTGTAATTTGTTTTACTGGGGGGTTAACCTAACAACTGCACACTCGTCACACACTCACATC	81	242	6	83.98	1	194	1
	GGGTCTTGTTTCTTCTCTGTGTGTAATTTGTTTTACTTGGGGGGTTGAACCTAACAAGTGACACACTCGTCACACACTCACATC	84	242	6	83.98	1	195	3
	GGGTCTTGTTTCTTCTCTGTGTGTAATTTGTTTTACTGGGGGGGTTGAACCTAACAAGTGACACACTCGTCACACACTCACATC	83	242	6	83.98	1	196	2
	GGGTCTTGTTTCTTCTCTGTGTGTAATTTGTTTTACTGGGGGGTTAACCTAACAAGTGCACACTCGTCACACACCACATC	80	242	6	83.98	1	197	1
	GGGTCTTGTTTCTTCTCTGTGTGTAATTTGTTTTACTGGGGGGTTAACCTAACAAGTGCACGCACTCGTCACACACTCACATC	83	242	6	83.98	1	198	2
	GGGTCTTGTTTCTTCTCTGTGTGTAATTGTTTTACTGGGGGGTTAACCTAACAAGCGCACACTCGTCACACACTCACATC	80	317	5	69.98	1	199	2
	GGTGCTTGTTTCTTCTCTGTGTGTAATTTGTTTTACTGGGGGGTTAACCTAACAAGTGCACACTCGTCACACACTCACATC	81	317	5	69.98	1	200	2
	GGGTCTTGTTTCTTCTCTGTGTGTAATTTGTTTTACTGGGGGGTTAACCTAACAAGTGCACACTCGTCACACTACACATC	80	317	5	69.98	1	201	3
	GAGTCTTGTTTCTTCTCTGTGTGTAATTTGTTTTACTGGGGGTTAACCTAACAAGTGCACACTCGTCACACACTCACATC	80	317	5	69.98	1	202	2
	GGGTCGTTGTTTCTTCTCTGTGTGTAATTTGTTTTACTGGGGGGTTAACCTAACAAGTGCACACTCGTCACACACTCACATC	82	317	5	69.98	1	203	1
	GGGTCTTGTTTCTTCTCTGTGTGTAATTTGTTTTACTGGGGGGTTACCTAACAAGTGCACACTCGTCACACACTCACATC	80	317	5	69.98	1	204	1
	GGGTCTTGTTTCTTCCTGTGTGTAATTTGTTTTACTGGGGGGTTAACCTAACAAGTGCACACTCGTCACACACTCACATC	80	317	5	69.98	1	205	1
	GGGTCTTGTTTCTTCTCTGTGTGTAATTTGTTTTACTGGGGGGTTAACCTAACAATGTGCACACTCGTCACACACTCACATC	82	317	5	69.98	1	206	1
	GGGTCTTGTTTCTTCTCTGTGTGTAATTTGTTCTACTGGGGGGGTTAACCTAACAAGTGCACACTCGTCACACACTCACATC	82	317	5	69.98	1	207	2
	AGGGTCTTGTTTCTTCTCTGTGTGTAATTTGTTTTACTGGGGGGGTTAACCTAACAAGTGCACACTCGTCACACACTCACATC	83	317	5	69.98	1	208	2
	GGGTCTTGTTTCTTCTCTGTGTGTAATTTGTTTTACTGGGGGGTTAACCTAACAAATTGCACACTCGTCACACACTCACATC	82	317	5	69.98	1	209	2
	GGGTCTTGTTTCTTCTCTGTGTGTAATTTTGTTTTACTGGGGGGTTAACCTAACAAGTGCACTCGTCACACACTCACATC	80	317	5	69.98	1	210	3
	GGGTCTTGTTTCTTCTCTGTGTGTAATTTGTTTTACTGGGGGGTTAACCTAACAAGTGCACACTCGTCACACGCTCACATC	81	317	5	69.98	1	211	1
	GGGTCTTGTTTCTTCTCTGTGTGTAATTTGTTTTACTGGGGGGTTAACCTAACAAGTGCACACCTCGTCACACACTCACATC	82	317	5	69.98	1	212	1
	GAGTCTTGTTTCTTCTCTGTGTGTAATTTGTTTTACTGGGGGGTTAACCTAACAAGCGCACACTCGTCACACACTCACATC	81	317	5	69.98	1	213	2
	GGGTCTTGTTTCTTCTCTGTGTGTAATTTGTTTTACTGGGGGGTTAACCATACAAGTGCACACTCGTCACACACTCACATC	81	317	5	69.98	1	214	2
	GGGTCTTGTTTCTTCTCTGTGTGTGATTTGTTTTACTGGGGGGTTGACCTAACAAGTGCACACTCGTCACACACTCACATC	81	317	5	69.98	1	215	2
	GGGTCTTGTTTCTTCTCTGTGTGTAATTTGTTTTACTGGGGGGTTAACCTAACAAGTGCACACTCGTCACACACTCAACATC	82	317	5	69.98	1	216	1
	GGGTCTTGTTTCTTCTCTGTGTGTAATTTGTTTTACTGGGGGGTTCACCTAACAAGTGCACACTCGTCACACACTCACATC	81	317	5	69.98	1	217	1
	GGGTCTTGTTTCTTCTCTGTGTGTAATTTGTTTTACTGGGGGGCTAACCTAACAAGTACACACTCGTCACACACTCACATC	81	317	5	69.98	1	218	2
	GGTCTTGTTTCTTCTCTGTGTGTAATTTGTTTTACTGGGGGGTTAACCTAACAAGTGTACACTCGTCACACACTCACATC	80	317	5	69.98	1	219	2
	GGGTCTTGTTTCTTCTCTGTGTGTAATTTGTTTTTACTGGGGGTTAACCTAACAAGTGCACACTCGTCACACACTCACATC	81	317	5	69.98	1	220	2
	GGGGTCTTGTTTCTTCTCTGTGTGTAATTTGTTTTACTGGGGGGTTAACCTAACAAGTGCACACTCGTCACACACTCACATC	82	317	5	69.98	1	221	1
	GGGTCTTGTTTCTTCTCTGTGTGTAATTTGTTTTACTGGGGGGTTAACCTAACAAGTGCACACTCGTCACACACTCCATC	80	317	5	69.98	1	222	1
	GGGTCTTGTTTCTTCTCTGTGTGTAATTTGTTTTACTGGGGGGTTAACCTAACTAAGTGCACACTCGTCACACACTCACATC	82	317	5	69.98	1	223	1
	GGGTCTTGTTTCTTCTCTGTGTGTAATTTGTTTTACTGGGGGGTTAACCTAACAAGTCACACTCGTCACACACTCACATC	80	317	5	69.98	1	224	1
	GGTCTTGTTTCTTCTCTGTGTGTAATTTGTTTTACTGGGGGTTAACCTAACAAGTGCACACTCGTCACACACTCACATC	79	317	5	69.98	1	225	2
	GGGTCTTGTTTCTTCTCTGTGTGTAATTTGTTTTACTGGGGGGGGTTAACCTAACAAGTGCACACTCGTCACACACTCACATC	83	317	5	69.98	1	226	2
	GGGTCTTGTTTCTTCTCTGTGTGTAATGTTGTCTTACTGGGGGGTTAACCTAACAAGTGCACACTCGTCACACACTCACATC	82	317	5	69.98	1	227	2
	GGGTCTTGTTTCTTCTCTGTGTGTAATATGTTTTACTGGGGGGTTAACCTAACAAGTGCACACTCGTCACACACTCACATC	81	317	5	69.98	1	228	1
	GGGTCTTGTTGCTTCTCTGTGTGTAATTTGTTTTACTGGGGGGTTAACCTAACAAGTGCACACTCGTCACACACTCACATC	81	317	5	69.98	1	229	1
	GGGTCTTGTTTCTTCTCTGTGTGTAATTTGTTTTACTGGGGGTTAACCTAACAATTGCACACTCGTCACACACTCACATC	80	317	5	69.98	1	230	2
	GGGTCTTGTTTCTTCTCTGTGTGTAATTTGTTTTACTGGGGGGTAAACCTAACAAGTGCACACTCGTCACACACTCACATC	81	317	5	69.98	1	231	1
	GGGTCTTGTTTCTTCTCTGTGTGTAATTTGTTTTACTGGGGGCTAACCTAACAAGTGCACACTCGTCACACACTCACATC	80	317	5	69.98	1	232	2
	GGGTCTTGTTTCTTCCTCTGTGTGTAATTTGTTTTACTGGGGGGTTAACCTAACAAGTGCACACTCGTCACACACTCACATC	82	317	5	69.98	1	233	1
	GGGTCTTGTTTCTTCTCTGTGTGTAATTTGTTTTACTGGGGGGTTAACCTAACAAGTGCACACTCGTCCACACACTCACATC	82	317	5	69.98	1	234	1
	GAGTCTTGTTTCTTCTCTGTGTGTAATTTGTTTTGACTGGGGGTTAACCTAACAAGTGCACACTCGTCACACACTCACATC	81	419	4	55.98	1	235	3
	GGGTCTTGTTTCTTCTCTGTGTGTGCTTTGTTTTACTGGGGGGTTAACCTAACAAGTGCACACTCGTCACACACTCACATC	81	419	4	55.98	1	236	2
	GGGTCTTGTTTCTTCTCTGTGTGTAATTGTTTACTGGGGGTTAACCTAACAAGTGCACACTCGTCACACACTCACATC	78	419	4	55.98	1	237	3
	GGGTCTTGTTTCTTCTCTGTGTGTAATTTGTTTTACTGGGGGGTTAACCTAACAAGTGCACACTCGTCACACTACTACATC	81	419	4	55.98	1	238	2
	GGGTCTTGTTTCTTCTCTGTGTGTAATTTGTTTTACTGGGGGGTTAACCTAACAAAGTACACACTCGTCACACACTCACATC	82	419	4	55.98	1	239	2
	GGGTCTTGTTTCTTCTCTGTGTGTAATTTGATTTACTGGGGGGTTAACCTAACAAGTGCACACTCGTCACACACTCACATC	81	419	4	55.98	1	240	1
	GGGTCTTGTTTCTTCTCTGTGCGTAATTTGTTTTACTGGGGGGTTAACCTAACAAGTGCACTCGTCACACACTCACATC	79	419	4	55.98	1	241	3
	GGGTCTTGTTTCTTCTCTGTGTGTAATTTGTTTTTTGGGGGGTTAACCTAACAAGTGCACACTCGTCACACACTCACATC	80	419	4	55.98	1	242	2
	GGGTCTTGTTTCTTCTCAGTGTGTAATTTGTTTTACTGGGGGGTTAACCTAACAAGTGCACACTCGTCACACACTCACATC	81	419	4	55.98	1	243	1
	GGGTCTTGTTTCTTCTCTGTGTGTAATTTGTTTTACTGGGGGGTTAACCTAACAAGCGCGCACTCGTCACACACTCACATC	81	419	4	55.98	1	244	2
	GGGTCTTGTTTCTTCTCTATGTGTAATTTGTTTTACTGGGGGGTTAACCTAACAAGTGCACACTCGTCACACACTCACATC	81	419	4	55.98	1	245	1
	GGGTCTTGTTTCTTCTCTGTGTGCAATTTGTTTTACTGGGGGGTTAACCTAACAAGCGCACACTCGTCACACACTCACATC	81	419	4	55.98	1	246	2
	GGGTCTTGTTTCTTCTCTGTGTGATTTGTTTTACTGGGGGGTTAACCTAACAAGTGCACACTCGTCACACACTCACATC	79	419	4	55.98	1	247	2
	GGGTCTTGTTTCTTCTCTGTGTGTGGTTTGTTTTACTGGGGGGTTAACCTAACAAGTGCACACTCGTCACACACTCACATC	81	419	4	55.98	1	248	2
	GGGTCTTGTTTCTTCTCTGTGTGTAATTTGTTTTACTGGGGGGCTAACCTAACAAGTGCGCACTCGTCACACACTCACATC	81	419	4	55.98	1	249	2
	GGGTCTTGTTTCTTCTCTGTGTGTAATTTGTTTTACTGGGGGGTTAACCTATCCTGTGCGCACTCGTCACACACTCACATC	81	419	4	55.98	1	250	4
	GGGTCTTGTTTCTTCTCTGTGTGTAATTTGTTTTACTGGGGGGGTCAACCTAACAAGTGCACACTCGTCACACACTCACATC	82	419	4	55.98	1	251	2
	GGGTCTTGTTTCTTCTCTTGTGTGTAATTTGTTTTACTGGGGGGTTAACCTAACAAGTGCACACTCGTCACACACTCACATC	82	419	4	55.98	1	252	1
	GGGTCTTGTTTCTTCTCTGTGTGTAATTTGTTTTACTGGGGGGTTAACCTGACAAGTGCGCACTCGTCACACACTCACATC	81	419	4	55.98	1	253	2
	GGGTCTTGTTTCTTCTCTGTGTGTAATTTGTTTTACTGGGGGTTAACCTAACAGGTGCACACTCGTCACACACTCACATC	80	419	4	55.98	1	254	2
	GGGTCTTGTTTCTTCTCTGTGTGTAATTTGTTTTACTGGGGGGTTAACCTGACCGGTGCACACTCGTCACACACTCACATC	81	419	4	55.98	1	255	3
	GGGTCTTGTTTCTTTCTGTGTGTAATTTGTTTTACTGGGGGGTTAACCTAACAAGTGCACACTCGTCACACACTCACATC	80	419	4	55.98	1	256	1
	GGGTCTTGTTTCTTCTCTGTGTGTAATTTGTTTTACTGGGGGCTTAACCTAACAAGTGCACACTCGTCACACACTCACATC	81	419	4	55.98	1	257	1
	GGGTCTTGTTTCTTCTCTGTGTGTAATTTGTTTTACTGGGGGGTTAACCTAACAGTAGCACACTCGTCACACACTCACATC	81	419	4	55.98	1	258	2
	GGGTCTTGTTTCTTCTCTGTGTGTGATTTGTTTTACTGGGGGGTTAACCTAACAAAGTGCACACTCGTCACACACTCACATC	82	419	4	55.98	1	259	2
	GGGTCTTGTTTCTTCTCGTGTGTAATTTGTTTTACTGGGGGGTTAACCTAACAAGTGCACACTCGTCACACACTCACATC	80	419	4	55.98	1	260	1
	GGGTCTTGTTTCTTCTCTGTGTGTAATTTGTTTTACTGGGGAGGTTAACCTAACAAGTGCACACTCGTCACACACTCACATC	82	419	4	55.98	1	261	1
	GGGTCTTGTTTCTTCTCTGTGTGTAATTTGTTTTACTGGGGGGTTAATCCTAACAAGTGGCACACTCGTCACACACTCACATC	83	419	4	55.98	1	262	2
	GGGTCTTGTTTCTTCTCTGTGTGTAATTTGTTTTTACTGGGGGGTTAACCTAACAAGCGCACACTCGTCACACACTCACATC	82	419	4	55.98	1	263	2
	GGGTCTTGTTTCTTCTCTGTGTGTAACTTGTTTTACTGGGGGGGTTAACCTAACAAGTGCACACTCGTCACACACTCACATC	82	419	4	55.98	1	264	2
	AGGGTCTTGTTTCTTCTCTGTGTGTAATTTTGTTTTTACTGGGGGGGTTAACCTAACAAGTGCACACTCGTCACACACTCACATC	85	419	4	55.98	1	265	4
	GGGTCTTGTTTCTTCTCTGTGTGTAATTTGTTTTTTACTGGGGGGTTAACCTAACAAGTGCACACTCGTCACACACTCACATC	83	419	4	55.98	1	266	2
	GGGTCTTGTTTCTTCTCTGTGTGTAATTTGTGTTACTGGGGGGTTAACCTAACAAGTGCACACTCGTCACACACTCACATC	81	419	4	55.98	1	267	1
	GGGTCTTGTTTCTTCTCTGTGTAGTTTGTTTTACTGGGGGGTTAACCTAACAAGTGCACACTCGTCACACACTCACATC	79	419	4	55.98	1	268	3
	GGGTCTCGTTTCTTCTCTGTGTGTAATTTGTTTTACTGGGGGGTTAACCTAACAAGTGCACACTCGTCACACACTCACATC	81	419	4	55.98	1	269	1
	GGGTCTTGTTTCTTCTCTGTGTGTAATTTGTTTTACTGGGGGGGTTAACCTAACAAGTACACACTCGTCACACACTCACATC	82	419	4	55.98	1	270	2
	GGGTCTTGTTTCTTCTCTGTGTGTAATTTGTTTTACTGGGGGGTTAACCTAAAGTGCACACTCGTCACACACTCACATC	79	419	4	55.98	1	271	2
	GGGTCTTGTTTCTTCTCTGTGTGTAATTTGTTTTACTGGGGGGTTAACCTAACAGGTGCGCACTCGTCACACACTCACATC	81	419	4	55.98	1	272	2
	GGGTCTTGTTTCTTCTCTGTGTGTAATTTGTTTTACTGGGGGGTTAACCTAACAAGTGCACACTCGTCACACACTCGCATC	81	419	4	55.98	1	273	1
	GGGTCTTGTTTCTTCTCTGTGTGTAATTTGTTATACTGGGGGGTTAACCTAACAAGTGCACACTCGTCACACACTCACATC	81	419	4	55.98	1	274	1
	GGGTCTTGTTTCTTCTCTGTGTGTAATTTGTTTTACTGGGGGGTTAACCTAAACAAGTGCACACTCGTCACACACTCACATC	82	419	4	55.98	1	275	1

*1. Name was given only to the sequence with the highest RPM value within all sequences .
*2. Length is the nucleotide bases of each sequence.
*3. Rank is the order of sequences after sorting by reads.
*4. Reads is the raw abundance of each sequence.
*5. RPM is read per million, which is the value normalized by the total population size.
*6. Cluster is the number of each cluster. The cluster is a subset of closely related sequences.
*7. Rank in cluster is the order of sequences after sorting by reads within the cluster.
*8. Edit distance is the total number of insertions, deletions, or substitutions required to transform one sequence into the sequence with the highest RPM in the cluster (also referred to as "seed sequence").

Note S1. DENV-1 genome sequence from NCBI reference sequence NC_001477.1 and the targeted sequences of the siRNAs.

AGTTGTTAGTCTACGTGGACCGACAAGAACAGTTTCGAATCGGAAGCTTGCTTAACGTAGTTCTAACAGTTTTTTAT
TAGAGAGCAGATCTCTGATGAACAACCAACGGAAAAAGACGGGTCGACCGTCTTTCAATATGCTGAAACGCGCGAGA
AACCGCGTGTCAACTGTTTCACAGTTGGCGAAGAGATTCTCAAAAGGATTGCTTTCAGGCCAAGGACCCATGAAATT
GGTGATGGCTTTTATAGCATTCCTAAGATTTCTAGCCATACCTCCAACAGCAGGAATTTTGGCTAGATGGGGCTCAT
TCAAGAAGAATGGAGCGATCAAAGTGTTACGGGGTTTCAAGAAAGAAATCTCAAACATGTTGAACATAATGAACAGG
AGGAAAAGATCTGTGACCATGCTCCTCATGCTGCTGCCCACAGCCCTGGCGTTCCATCTGACCACCCGAGGGGGAGA
GCCGCACATGATAGTTAGCAAGCAGGAAAGAGGAAAATCACTTTTGTTTAAGACCTCTGCAGGTGTCAACATGTGCA
CCCTTATTGCAATGGATTTGGGAGAGTTATGTGAGGACACAATGACCTACAAATGCCCCCGGATCACTGAGACGGAA
CCAGATGACGTTGACTGTTGGTGCAATGCCACGGAGACATGGGTGACCTATGGAACATGTTCTCAAACTGGTGAACA
CCGACGAGACAAACGTTCCGTCGCACTGGCACCACACGTAGGGCTTGGTCTAGAAACAAGAACCGAAACGTGGATGT
CCTCTGAAGGCGCTTGGAAACAAATACAAAAAGTGGAGACCTGGGCTCTGAGACACCCAGGATTCACGGTGATAGCC
CTTTTTCTAGCACATGCCATAGGAACATCCATCACCCAGAAAGGGATCATTTTTATTTTGCTGATGCTGGTAACTCC
ATCCATGGCCATGCGGTGCGTGGGAATAGGCAACAGAGACTTCGTGGAAGGACTGTCAGGAGCTACGTGGGTGGATG
TGGTACTGGAGCATGGAAGTTGCGTCACTACCATGGCAAAAGACAAACCAACACTGGACATTGAACTCTTGAAGACG
GAGGTCACAAACCCTGCCGTCCTGCGCAAACTGTGCATTGAAGCTAAAATATCAAACACCACCACCGATTCGAGATG
TCCAACACAAGGAGAAGCCACGCTGGTGGAAGAACAGGACACGAACTTTGTGTGTCGACGAACGTTCGTGGACAGAG
GCTGGGGCAATGGTTGTGGGCTATTCGGAAAAGGTAGCTTAATAACGTGTGCTAAGTTTAAGTGTGTGACAAAACTG
GAAGGAAAGATAGTCCAATATGAAAACTTAAAATATTCAGTGATAGTCACCGTACACACTGGAGACCAGCACCAAGT
TGGAAATGAGACCACAGAACATGGAACAACTGCAACCATAACACCTCAAGCTCCCACGTCGGAAATACAGCTGACAG
ACTACGGAGCTCTAACATTGGATTGTTCACCTAGAACAGGGCTAGACTTTAATGAGATGGTGTTGTTGACAATGAAA
AAAAAATCATGGCTCGTCCACAAACAATGGTTTCTAGACTTACCACTGCCTTGGACCTCGGGGGCTTCAACATCCCA
AGAGACTTGGAATAGACAAGACTTGCTGGTCACATTTAAGACAGCTCATGCAAAAAAGCAGGAAGTAGTCGTACTAG
GATCACAAGAAGGAGCAATGCACACTGCGTTGACTGGAGCGACAGAAATCCAAACGTCTGGAACGACAACAATTTTT
GCAGGACACCTGAAATGCAGATTAAAAATGGATAAACTGATTTTAAAAGGGATGTCATATGTAATGTGCACAGGGTC
ATTCAAGTTAGAGAAGGAAGTGGCTGAGACCCAGCATGGAACTGTTCTAGTGCAGGTTAAATACGAAGGAACAGATG
CACCATGCAAGATCCCCTTCTCGTCCCAAGATGAGAAGGGAGTAACCCAGAATGGGAGATTGATAACAGCCAACCCC
ATAGTCACTGACAAAGAAAAACCAGTCAACATTGAAGCGGAGCCACCTTTTGGTGAGAGCTACATTGTGGTAGGAGC
AGGTGAAAAAGCTTTGAAACTAAGCTGGTTCAAGAAGGGAAGCAGTATAGGGAAAATGTTTGAAGCAACTGCCCGTG
GAGCACGAAGGATGGCCATCCTGGGAGACACTGCATGGGACTTCGGTTCTATAGGAGGGGTGTTCACGTCTGTGGGA
AAACTGATACACCAGATTTTTGGGACTGCGTATGGAGTTTTGTTCAGCGGTGTTTCTTGGACCATGAAGATAGGAAT
AGGGATTCTGCTGACATGGCTAGGATTAAACTCAAGGAGCACGTCCCTTTCAATGACGTGTATCGCAGTTGGCATGG
TCACACTGTACCTAGGAGTCATGGTTCAGGCGGACTCGGGATGTGTAATCAACTGGAAAGGCAGAGAACTCAAATGT
GGAAGCGGCATTTTTGTCACCAATGAAGTCCACACCTGGACAGAGCAATATAAATTCCAGGCCGACTCCCCTAAGAG
ACTATCAGCGGCCATTGGGAAGGCATGGGAGGAGGGTGTGTGTGGAATTCGATCAGCCACTCGTCTCGAGAACATCA
TGTGGAAGCAAATATCAAATGAATTAAACCACATCTTACTTGAAAATGACATGAAATTTACAGTGGTCGTAGGAGAC

GTTAGTGGAATCTTGGCCCAAGGAAAGAAAATGATTAGGCCACAACCCATGGAACACAAATACTCGTGGAAAAGCTG
GGGAAAAGCCAAAATCATAGGAGCAGATGTACAGAATACCACCTTCATCATCGACGGCCCAAACACCCCAGAATGCC
CTGATAACCAAAGAGCATGGAACATTTGGGAAGTTGAAGACTATGGATTTGGAATTTTCACGACAAACATATGGTTG
AAATTGCGTGACTCCTACACTCAAGTGTGTGACCACCGGCTAATGTCAGCTGCCATCAAGGATAGCAAAGCAGTCCA
TGCTGACATGGGGTACTGGATAGAAAGTGAAAAGAACGAGACTTGGAAGTTGGCAAGAGCCTCCTTCATAGAAGTTA
AGACATGCATCTGGCCAAAATCCCACACTCTATGGAGCAATGGAGTCCTGGAAAGTGAGATGATAATCCCAAAGATA
TATGGAGGACCAATATCTCAGCACAACTACAGACCAGGATATTTCACACAAACAGCAGGGCCGTGGCACTTGGGCAA
GTTAGAACTAGATTTTGATTTATGTGAAGGTACCACTGTTGTTGTGGATGAACATTGTGGAAATCGAGGACCATCTC
TTAGAACCACAACAGTCACAGGAAAGACAATCCATGAATGGTGCTGTAGATCTTGCACGTTACCCCCCCTACGTTTC
AAAGGAGAAGACGGGTGCTGGTACGGCATGGAAATCAGACCAGTCAAGGAGAAGGAAGAGAACCTAGTTAAGTCAAT
GGTCTCTGCAGGGTCAGGAGAAGTGGACAGTTTTTCACTAGGACTGCTATGCATATCAATAATGATCGAAGAGGTAA
TGAGATCCAGATGGAGCAGAAAAATGCTGATGACTGGAACATTGGCTGTGTTCCTCCTTCTCACAATGGGACAATTG
ACATGGAATGATCTGATCAGGCTATGTATCATGGTTGGAGCCAACGCTTCAGACAAGATGGGGATGGGAACAACGTA
CCTAGCTTTGATGGCCACTTTCAGAATGAGACCAATGTTCGCAGTCGGGCTACTGTTTCGCAGATTAACATCTAGAG
AAGTTCTTCTTCTTACAGTTGGATTGAGTCTGGTGGCATCTGTAGAACTACCAAATTCCTTAGAGGAGCTAGGGGAT
GGACTTGCAATGGGCATCATGATGTTGAAATTACTGACTGATTTTCAGTCACATCAGCTATGGGCTACCTTGCTGTC
TTTAACATTTGTCAAAACAACTTTTTCATTGCACTATGCATGGAAGACAATGGCTATGATACTGTCAATTGTATCTC
TCTTCCCTTTATGCCTGTCCACGACTTCTCAAAAAACAACATGGCTTCCGGTGTTGCTGGGATCTCTTGGATGCAAA
CCACTAACCATGTTTCTTATAACAGAAAACAAAATCTGGGGAAGGAAAAGCTGGCCTCTCAATGAAGGAATTATGGC
TGTTGGAATAGTTAGCATTCTTCTAAGTTCACTTCTCAAGAATGATGTGCCACTAGCTGGCCCACTAATAGCTGGAG
GCATGCTAATAGCATGTTATGTCATATCTGGAAGCTCGGCCGATTTATCACTGGAGAAAGCGGCTGAGGTCTCCTGG
GAAGAAGAAGCAGAACACTCTGGTGCCTCACACAACATACTAGTGGAGGTCCAAGATGATGGAACCATGAAGATAAA
GGATGAAGAGAGAGATGACACACTCACCATTCTCCTCAAAGCAACTCTGCTAGCAATCTCAGGGGTATACCCAATGT
CAATACCGGCGACCCTCTTTGTGTGGTATTTTTGGCAGAAAAAGAAACAGAGATCAGGAGTGCTATGGGACACACCC
AGCCCTCCAGAAGTGGAAAGAGCAGTCCTTGATGATGGCATTTATAGAATTCTCCAAAGAGGATTGTTGGGCAGGTC
TCAAGTAGGAGTAGGAGTTTTTCAAGAAGGCGTGTTCCACACAATGTGGCACGTCACCAGGGGAGCTGTCCTCATGT
ACCAAGGGAAGAGACTGGAACCAAGTTGGGCCAGTGTCAAAAAAGACTTGATCTCATATGGAGGAGGTTGGAGGTTT
CAAGGATCCTGGAACGCGGGAGAAGAAGTGCAGGTGATTGCTGTTGAACCGGGGAAGAACCCCAAAAATGTACAGAC
AGCGCCGGGTACCTTCAAGACCCCTGAAGGCGAAGTTGGAGCCATAGCTCTAGACTTTAAACCCGGCACATCTGGAT
CTCCTATCGTGAACAGAGAGGGAAAAATAGTAGGTCTTTATGGAAATGGAGTGGTGACAACAAGTGGTACCTACGTC
AGTGCCATAGCTCAAGCTAAAGCATCACAAGAAGGGCCTCTACCAGAGATTGAGGACGAGGTGTTTAGGAAAAGAAA
CTTAACAATAATGGACCTACATCCAGGATCGGGAAAAACAAGAAGATACCTTCCAGCCATAGTCCGTGAGGCCATAA
AAAGAAAGCTGCGCACGCTAGTCTTAGCTCCCACAAGAGTTGTCGCTTCTGAAATGGCAGAGGCGCTCAAGGGAATG
CCAATAAGGTATCAGACAACAGCAGTGAAGAGTGAACACACGGGAAAGGAGATAGTTGACCTTATGTGTCACGCCAC
TTTCACTATGCGTCTCCTGTCTCCTGTGAGAGTTCCCAATTATAATATGATTATCATGGATGAAGCACATTTTACCG
ATCCAGCCAGCATAGCAGCCAGAGGGTATATCTCAACCCGAGTGGGTATGGGTGAAGCAGCTGCGATTTTCATGACA
GCCACTCCCCCCGGATCGGTGGAGGCCTTTCCACAGAGCAATGCAGTTATCCAAGATGAGGAAAGAGACATTCCTGA
AAGATCATGGAACTCAGGCTATGACTGGATCACTGATTTCCCAGGTAAAACAGTCTGGTTTGTTCCAAGCATCAAAT

CAGGAAATGACATTGCCAACTGTTTAAGAAAGAATGGGAAACGGGTGGTCCAATTGAGCAGAAAAACTTTTGACACT
GAGTACCAGAAAACAAAAAATAACGACTGGGACTATGTTGTCACAACAGACATATCCGAAATGGGAGCAAACTTCCG
AGCCGACAGGGTAATAGACCCGAGGCGGTGCCTGAAACCGGTAATACTAAAAGATGGCCCAGAGCGTGTCATTCTAG
CCGGACCGATGCCAGTGACTGTGGCTAGCGCCGCCCAGAGGAGAGGAAGAATTGGAAGGAACCAAAATAAGGAAGGC
GATCAGTATATTTACATGGGACAGCCTCTAAACAATGATGAGGACCACGCCCATTGGACAGAAGCAAAAATGCTCCT
TGACAACATAAACACACCAGAAGGGATTATCCCAGCCCTCTTTGAGCCGGAGAGAGAAAAGAGTGCAGCAATAGACG
GGGAATACAGACTACGGGGTGAAGCGAGGAAAACGTTCGTGGAGCTCATGAGAAGAGGAGATCTACCTGTCTGGCTA
TCCTACAAAGTTGCCTCAGAAGGCTTCCAGTACTCCGACAGAAGGTGGTGCTTTGATGGGGAAAGGAACAACCAGGT
GTTGGAGGAGAACATGGACGTGGAGATCTGGACAAAAGAAGGAGAAAGAAAGAAACTACGACCCCGCTGGCTGGATG
CCAGAACATACTCTGACCCACTGGCTCTGCGCGAATTCAAAGAGTTCGCAGCAGGAAGAAGAAGCGTCTCAGGTGAC
CTAATATTAGAAATAGGGAAACTTCCACAACATTTAACGCAAAGGGCCCAGAACGCCTTGGACAATCTGGTTATGTT
GCACAACTCTGAACAAGGAGGAAAAGCCTATAGACACGCCATGGAAGAACTACCAGACACCATAGAAACGTTAATGC
TCCTAGCTTTGATAGCTGTGCTGACTGGTGGAGTGACGTTGTTCTTCCTATCAGGAAGGGGTCTAGGAAAAACATCC
ATTGGCCTACTCTGCGTGATTGCCTCAAGTGCACTGTTATGGATGGCCAGTGTGGAACCCCATTGGATAGCGGCCTC
TATCATACTGGAGTTCTTTCTGATGGTGTTGCTTATTCCAGAGCCGGACAGACAGCGCACTCCACAAGACAACCAGC
TAGCATACGTGGTGATAGGTCTGTTATTCATGATATTGACAGTGGCAGCCAATGAGATGGGATTACTGGAAACCACA
AAGAAGGACCTGGGGATTGGTCATGCAGCTGCTGAAAACCACCATCATGCTGCAATGCTGGACGTAGACCTACATCC
AGCTTCAGCCTGGACTCTCTATGCAGTGGCCACAACAATTATCACTCCCATGATGAGACACACAATTGAAAACACAA
CGGCAAATATTTCCCTGACAGCTATTGCAAACCAGGCAGCTATATTGATGGGACTTGACAAGGGATGGCCAATATCA
AAGATGGACATAGGAGTTCCACTTCTCGCCTTGGGGTGCTATTCTCAGGTGAACCCGCTGACGCTGACAGCGGCGGT
ATTGATGCTAGTGGCTCATTATGCCATAATTGGACCCGGACTGCAAGCAAAAGCTACTAGAGAAGCTCAAAAAAGGA
CAGCAGCCGGAATAATGAAAAACCCAACTGTCGACGGGATCGTTGCAATAGATTTGGACCCTGTGGTTTACGATGCA
AAATTTGAAAAACAGCTAGGCCAAATAATGTTGTTGATACTTTGCACATCACAGATCCTCCTGATGCGGACCACATG
GGCCTTGTGTGAATCCATCACACTAGCCACTGGACCTCTGACTACGCTTTGGGAGGGATCTCCAGGAAAATTCTGGA
ACACCACGATAGCGGTGTCCATGGCAAACATTTTTAGGGGAAGTTATCTAGCAGGAGCAGGTCTGGCCTTTTCATTA
ATGAAATCTCTAGGAGGAGGTAGGAGAGGCACGGGAGCCCAAGGGGAAACACTGGGAGAAAAATGGAAAAGACAGCT
AAACCAATTGAGCAAGTCAGAATTCAACACTTACAAAAGGAGTGGGATTATAGAGGTGGATAGATCTGAAGCCAAAG
AGGGGTTAAAAAGAGGAGAAACGACTAAACACGCAGTGTCGAGAGGAACGGCCAAACTGAGGTGGTTTGTGGAGAGG
AACCTTGTGAAACCAGAAGGGAAAGTCATAGACCTCGGTTGTGGAAGAGGTGGCTGGTCATATTATTGCGCTGGGCT
GAAGAAAGTCACAGAAGTGAAAGGATACACGAAAGGAGGACCTGGACATGAGGAACCAATCCCAATGGCAACCTATG
GATGGAACCTAGTAAAGCTATACTCCGGGAAAGATGTATTCTTTACACCACCTGAGAAATGTGACACCCTCTTGTGT
GATATTGGTGAGTCCTCTCCGAACCCAACTATAGAAGAAGGAAGAACGTTACGTGTTCTAAAGATGGTGGAACCATG
GCTCAGAGGAAACCAATTTTGCATAAAAATTCTAAATCCCTATATGCCGAGTGTGGTAGAAACTTTGGAGCAAATGC
AAAGAAAACATGGAGGAATGCTAGTGCGAAATCCACTCTCAAGAAACTCCACTCATGAAATGTACTGGGTTTCATGT
GGAACAGGAAACATTGTGTCAGCAGTAAACATGACATCTAGAATGCTGCTAAATCGATTCACAATGGCTCACAGGAA
GCCAACATATGAAAGAGACGTGGACTTAGGCGCTGGAACAAGACATGTGGCAGTAGAACCAGAGGTGGCCAACCTAG
ATATCATTGGCCAGAGGATAGAGAATATAAAAAATGAACACAAATCAACATGGCATTATGATGAGGACAATCCATAC
AAAACATGGGCCTATCATGGATCATATGAGGTCAAGCCATCAGGATCAGCCTCATCCATGGTCAATGGTGTGGTGAG

ACTGCTAACCAAACCATGGGATGTCATTCCCATGGTCACACAAATAGCCATGACTGACACCACACCCTTTGGACAAC
AGAGGGTGTTTAAAGAGAAAGTTGACACGCGTACACCAAAAGCGAAACGAGGCACAGCACAAATTATGGAGGTGACA
GCCAGGTGGTTATGGGGTTTTCTCTCTAGAAACAAAAAACCCAGAATCTGCACAAGAGAGGAGTTCACAAGAAAAGT
CAGGTCAAACGCAGCTATTGGAGCAGTGTTCGTTGATGAAAATCAATGGAACTCAGCAAAAGAGGCAGTGGAAGATG
AACGGTTCTGGGACCTTGTGCACAGAGAGAGGGAGCTTCATAAACAAGGAAAATGTGCCACGTGTGTCTACAACATG
ATGGGAAAGAGAGAGAAAAAATTAGGAGAGTTCGGAAAGGCAAAAGGAAGTCGCGCAATATGGTACATGTGGTTGGG
AGCGCGCTTTTTAGAGTTTGAAGCCCTTGGTTTCATGAATGAAGATCACTGGTTCAGCAGAGAGAATTCACTCAGTG
GAGTGGAAGGAGAAGGACTCCACAAACTTGGATACATACTCAGAGACATATCAAAGATTCCAGGGGGAAATATGTAT
GCAGATGACACAGCCGGATGGGACACAAGAATAACAGAGGATGATCTTCAGAATGAGGCCAAAATCACTGACATCAT
GGAACCTGAACATGCCCTATTGGCCACGTCAATCTTTAAGCTAACCTACCAAAACAAGGTAGTAAGGGTGCAGAGAC
CAGCGAAAAATGGAACCGTGATGGATGTCATATCCAGACGTGACCAGAGAGGAAGTGGACAGGTTGGAACCTATGGC
TTAAACACCTTCACCAACATGGAGGCCCAACTAATAAGACAAATGGAGTCTGAGGGAATCTTTTCACCCAGCGAATT
GGAAACCCCAAATCTAGCCGAAAGAGTCCTCGACTGGTTGAAAAAACATGGCACCGAGAGGCTGAAAAGAATGGCAA
TCAGTGGAGATGACTGTGTGGTGAAACCAATCGATGACAGATTTGCAACAGCCTTAACAGCTTTGAATGACATGGGA
AAGGTAAGAAAAGACATACCGCAATGGGAACCTTCAAAAGGATGGAATGATTGGCAACAAGTGCCTTTCTGTTCACA
CCATTTCCACCAGCTGATTATGAAGGATGGGAGGGAGATAGTGGTGCCATGCCGCAACCAAGATGAACTTGTAGGTA
GGGCCAGAGTATCACAAGGCGCCGGATGGAGCTTGAGAGAAACTGCATGCCTAGGCAAGTCATATGCACAAATGTGG
CAGCTGATGTACTTCCACAGGAGAGACTTGAGATTAGCGGCTAATGCTATCTGTTCAGCCGTTCCAGTTGATTGGGT
CCCAACCAGCCGCACCACCTGGTCGATCCATGCCCACCATCAATGGATGACAACAGAAGACATGTTGTCAGTGTGGA
ATAGGGTTTGGATAGAGGAAAACCCATGGATGGAGGACAAGACTCATGTGTCCAGTTGGGAAGACGTTCCATACCTA
GGAAAAAGGGAAGATCAATGGTGTGGTTCCCTAATAGGCTTAACAGCACGAGCCACCTGGGCCACCAACATACAAGT
GGCCATAAACCAAGTGAGAAGGCTCATTGGGAATGAGAATTATCTAGACTTCATGACATCAATGAAGAGATTCAAAA
ACGAGAGTGATCCCGAAGGGGCACTCTGGTAAGCCAACTCATTCACAAAATAAAGGAAAATAAAAAATCAAACAAGG
CAAGAAGTCAGGCCGGATTAAGCCATAGCACGGTAAGAGCTATGCTGCCTGTGAGCCCCGTCCAAGGACGTAAAATG
AAGTCAGGCCGAAAGCCACGGTTCGAGCAAGCCGTGCTGCCTGTAGCTCCATCGTGGGGATGTAAAAACCCGGGAGG
CTGCAAACCATGGAAGCTGTACGCATGGGGTAGCAGACTAGTGGTTAGAGGAGACCCCTCCCAAGACACAACGCAGC
AGCGGGGCCCAACACCAGGGGAAGCTGTACCCTGGTGGTAAGGACTAGAGGTTAGAGGAGACCCCCCGCACAACAAC
AAACAGCATATTGACGCTGGGAGAGACCAGAGATCCTGCTGTCTCTACAGCATCATTCCAGGCACAGAACGCCAAAA
AATGGAATGGTGCTGTTGAATCAACAGGTTCT

*Sequences highlighted in gray were artificially synthesized as oligonucleotide and inserted into the 3’UTR of the *Renilla luciferase* gene in a psiCHECK2 vector for the reporter assay.

*Sequences surrounded by squares indicate the start and stop codons of the ORF in DENV-1.

*Sequences with underlines were the targeted sequences of six siRNAs.
